# *PALB2*-mutated human mammary cells display a broad spectrum of morphological and functional abnormalities induced by increased TGFβ signaling

**DOI:** 10.1007/s00018-024-05183-6

**Published:** 2024-04-10

**Authors:** Hanna Tuppurainen, Niina Laurila, Marjut Nätynki, Leila Eshraghi, Anna Tervasmäki, Louisa Erichsen, Claus Storgaard Sørensen, Katri Pylkäs, Robert Winqvist, Hellevi Peltoketo

**Affiliations:** 1 Laboratory of Cancer Genetics and Tumor Biology, Translational Medicine Research Unit, Biocenter Oulu and Faculty of Medicine, Medical Research Center Oulu, University of Oulu, Oulu, Finland; 2 Present Address: Garvan Institute of Medical Research, Sydney, Australia; 3 Biotech Research and Innovation Centre (BRIC), University of Copenhagen, Copenhagen, Denmark; 4 Northern Finland Laboratory Centre, Oulu, Finland

**Keywords:** Hereditary breast cancer susceptibility modeling, *PALB2*-edited cell lines, Three-dimensional cell growth and migration, Transcriptome profiling, TGFβ response, KRT14-positive cells

## Abstract

Heterozygous mutations in any of three major genes, *BRCA1*, *BRCA2* and *PALB2*, are associated with high-risk hereditary breast cancer susceptibility frequently seen as familial disease clustering. PALB2 is a key interaction partner and regulator of several vital cellular activities of BRCA1 and BRCA2, and is thus required for DNA damage repair and alleviation of replicative and oxidative stress. Little is however known about how PALB2-deficiency affects cell function beyond that, especially in the three-dimensional setting, and also about its role during early steps of malignancy development. To answer these questions, we have generated biologically relevant MCF10A mammary epithelial cell lines with mutations that are comparable to certain clinically important *PALB2* defects. We show in a non-cancerous background how both mono- and biallelically *PALB2*-mutated cells exhibit gross spontaneous DNA damage and mitotic aberrations. Furthermore, PALB2-deficiency disturbs three-dimensional spheroid morphology, increases the migrational capacity and invasiveness of the cells, and broadly alters their transcriptome profiles. TGFβ signaling and *KRT14* expression are enhanced in *PALB2*-mutated cells and their inhibition and knock down, respectively, lead to partial restoration of cell functions. KRT14-positive cells are also more abundant with DNA damage than KRT14-negative cells. The obtained results indicate comprehensive cellular changes upon *PALB2* mutations, even in the presence of half dosage of wild type PALB2 and demonstrate how *PALB2* mutations may predispose their carriers to malignancy.

**Supplementary Information:**

The online version contains supplementary material available at 10.1007/s00018-024-05183-6.

## Introduction

Pathogenic heritable gene variants are strongly involved in the etiology of breast carcinoma and estimated to cause as much as five to ten percent of this very common malignancy [1]. Based on its mutation frequency and disease penetrance, *PALB2* is considered as the third major high-risk breast cancer gene alongside *BRCA1* and *BRCA2* [1, 2], all of which being key factors for the maintenance of genome stability*.* A recent large multinational study demonstrated that monoallelic pathogenic *PALB2* germline variants predispose to female and male breast cancers with relative risks above 7, and by the age of 80 years 53% (CI 95%: 44–63%) of the female mutation carriers are predicted to develop the malignancy [3]. Carriers of monoallelic germline *PALB2* mutations are also more likely to develop pancreatic and ovarian cancer than non-carriers [3]. Furthermore, biallelic pathogenic germline mutations in *PALB2* cause type N Fanconi anemia that is manifested as childhood cancers [4, 5].

PALB2 is a multifunctional protein that coordinates the DNA damage response (DDR) operations of BRCA1 and BRCA2 to enable homologous recombination and maintain genomic integrity [6]. In addition to its vital role in resolving interstrand crosslinks and double-strand DNA breaks, PALB2 participates in the regulation of DNA replication, cell cycle function, as well as oxidative stress responses, chromatin modulation, and transcriptional elongation via several cooperating partners [7–10]. Therefore, lack of sufficient amount of intact PALB2 may have widespread and cumulative cellular consequences, also beyond functions primarily related to compromised DNA repair.

The role of PALB2 in DDR and chromatin binding has been elegantly described previously. However, the knowledge of further cell biological outcomes of inadequate PALB2 action, and particularly of those occurring in a non-cancerous and three-dimensional (3D) cellular context, remains incomplete. To investigate the variety and magnitude of cellular defects in greater detail, and in a biologically appropriate model system, human MCF10A mammary epithelial cells [11, 12] were modified by CRISPR/Cas9 gene editing to create bi- or monoallelically *PALB2*-mutated cell lines. Together with the functional and cell biological assessment of the isogenic *PALB2*-edited and control cells, we also carried out transcriptome profiling of 3D spheroids grown from these cell lines.

Our results demonstrate the appearance of multiple notable functional as well as morphological abnormalities in both bi- and monoallelically *PALB2*-mutated cell lines, the defects typically being more severe in the biallelic ones. The characteristics of defective DNA damage response and mitosis, and consequently p53 (gene alias *TP53*) activation were demonstrated in all PALB2-compromised cell lines *i.e*., in the cells that either expressed faulty protein variants or/and a decreased amount of the intact protein. TGFβ signaling pathway was also enhanced in *PALB2*-mutated cell lines. Importantly, the PALB2 deficiency also led to non-organized 3D growth of the spheroids and it increased the migratory capacity and invasiveness of the cells, even in monoallelically *PALB2*-mutated cells. The altered phenotypes were also associated with robust alterations in the transcriptome profiles of *PALB2*-mutated spheroids, pointing towards malignant transformation of the cells. The migratory phenotype could partially be reversed by inhibition of TGFβ receptor function and *KRT14* knock down (KD). These novel results further emphasize the vital role of PALB2 in maintaining the cellular balance against various biological stress factors. A comprehensive understanding of the key cellular functions of PALB2 and the disruption of these functions in mutation carrier individuals, may eventually provide new clues and means for developing more effective ways of cancer therapy, based on the underlying specific cancer predisposing germline gene defect.

## Materials and methods

### Generation and culturing of the isogenic MCF10A cell lines

MCF10A cells (ATCC^®^ #CRL-10317™, RRID:CVCL_0598, Lot SLBC5066) were purchased from Sigma-Aldrich and their authenticity has been reconfirmed (Supplementary Fig. 1). Mutations were introduced into the fourth and fifth exon of *PALB2* in parental cells by gene-editing following the protocol of Ran and co-workers [13] as described previously [14]. The cell lines targeting the different exons with corresponding isogenic controls were generated independently.

The gene-editing tools were delivered into cells with all-in-one plasmids pSpCas9(BB)-2A-GFP (PX458, RRID:Addgene_48138) and pSpCas9n(BB)-2A-GFP (PX461, RRID:Addgene_48140) that were gifts form Dr. Feng Zhang, using Nucleofector™ II system (Amaxa™), transfection kit L (Lonza #VACA-1005) and programs X-001 or X-005. Transfections were followed by fluorescence activated cell sorting, clonal expansion of cells and mutation screening. Clones with intact *PALB2* were selected for control cell lines; #Ctrl-45 and #Ctrl-2.52 for the cell lines with modified fourth and fifth exon, respectively. The enzymes, guideRNAs and primers used in the processes are listed, and more details are given in Supplementary Methods.

The cell lines were maintained as in [15], and they were regularly tested for mycoplasma contamination using MycoAlert^®^ Plus kit (Lonza #LT07-705). Spheroids were grown on top of growth factor-reduced basement membrane extracts (GFR-BME, Corning^®^ Matrigel^®^ #354230, Trevigen^®^ Cultrex^®^ #3533-010-02) according to the original MCF10A protocols [15, 16]. Passage-matched, isogenic cell lines were used for each experiment. Proliferation rates of the cell lines were determined using IncuCyte^®^ S3 live cell analysis system (Sartorius).

### Transcriptome sequencing and data analysis

Spheroids were grown as triplicates on top of GFR-BME (Trevigen^®^ Cultrex^®^ #3533-010-02**)** on 35 mm cell culture µ-dishes with a glass coverslip (Ibidi #81158) for eight days. They were collected with Cultrex^®^ 3D culture cell harvesting kit (Trevigen^®^ #3448-020-K) and RNA was extracted from them using RNeasy Micro RNA isolation kits (Qiagen #74004). The quality and quantity of total RNA was assessed with Qubit™ RNA Broad Range kit (Invitrogen™ #Q10210) and Agilent Bioanalyzer 2100 with RNA 6000 Nano kit (Agilent Technologies #5067-1511) and RNA integrity number of the samples ranged from 9.9 to 10.0. Libraries were prepared from 100 ng of RNA using TruSeq^®^ Stranded Total RNA LT Gold kit (Illumina #20020598). The quantity and quality of the libraries were assessed using Bioanalyzer 2100 with DNA 1000 Kit (Agilent Technologies #5067-1504), Qubit™ dsDNA Broad Range kit (Invitrogen™ #Q32853) and qPCR KAPA™ Library quantification kit (KAPABiosystems #KK4824). The libraries were sequenced using High Output kit v2 (Illumina #20024907) and Illumina NextSeq550 platform in pair-ended 76 + 76 cycle mode, followed by FASTQ file generation and FASTQC quality control within BaseSpace^®^ computing environment (Illumina, RRID:SCR_011881). The minimum number of reads per sample was 52 M with average %Q30 values of 94.5%.

The FASTQ files were aligned to the *Homo sapiens* reference genome GRCh38/hg38 using TopHat2 (v2.1.1, RRID:SCR_013035) together with Bowtie2 (v2.2.9, RRID:SCR_016368), and the differential expression analysis to create a list of differentially expressed genes (DEGs) (*q*-value ≤ 0.05) in *PALB2* mutant cell lines was performed by DESeq2 (v1.1.0, RRID:SCR_015687) within Chipster CSC20 analysis software (CSC – IT Center for Science Ltd., RRID:SCR_010939). Each mutant cell line was compared to its own control. The results were confirmed by repeating the analysis using BaseSpace and Bioconductor/R (v3.3, RRID:SCR_006442) (details in Supplementary Methods). Principal component and heatmap analyses of the DEGs were created using DESeq2 in BaseSpace. Venn diagrams were drawn using the web tool at http://bioinformatics.psb.ugent.be/webtools/Venn/. Transcriptome sequencing results were verified using qRT-PCR as described in Supplementary Methods.

The lists of DEGs (Supplementary Table 1) weighted with their log2-fold change (L2fc) values (L2fc < − 0.5 or > 0.5) were analyzed with the Ingenuity Pathway Analysis (IPA) package (Qiagen, RRID:SCR_008653). To monitor the false discovery rate, Benjamini–Hochberg (B–H) corrected, right-tailed, Fisher’s exact test* p*-values < 0.05 were considered significant, as well as activation z-scores < − 0.2 or > 2, providing predictions about upstream or downstream processes. For visualization of the connections between the DEGs and biological processes involved, STRING (Search Tool for Retrieval of Interacting Genes/Proteins) database (v11.0, RRID:SCR_005223) was applied including DEGs with L2fc < − 0.8 or > 0.8. For the minimum required interaction score, high confidence (0.700) was applied with all seven independent interaction sources.

### Western blot analysis and immunocytochemistry

For Western blot and standard immunocytochemistry assays cells were grown as monolayer as described in [15]. To test DDR, cells were grown in the growth media supplemented with 0.05–2 µM etoposide (Pfizer #391870) for 5 h–3 days as indicated. For experimental series containing 3.3–50 pM TGFβ (Abcam #50038) and/or 1 µM LY2109761 (Selleckchem #S2704) -treatments cells were grown in the growth media with reduced EGF concentration (2 ng/ml) for 24 or 48 h as indicated. The final concentration of DMSO vehicle in the experimental media for LY2109761 treatment was 0.0025%. Spheroids were grown on top of GFR-BME-coated chamber slides for 8 days and stained as described [15, 16]. Proteins were quantified in Western blot analyses with total protein normalization. Protocols, antibodies and stains have been provided in detail in Supplementary Methods. Validation of PALB2 antibodies has been demonstrated in Supplementary Fig. 2a.

### Inhibition analysis

MCF10A cell lines were plated onto 96-well plates in 2 ng/ml EGF growth medium in triplicate. On the following day a concentration gradient (0, 1, 5, 10, 15, 20, 25, 30, 35 and 40 µM) of LY2109761 dissolved into DMSO was added the final concentration of the vehicle being 0.1% on cells. The half of the medium was bidaily replaced with one containing fresh inhibitor. Six days after plating, the cells were fixed, permeabilized and stained with a one-step method [0.25% PFA, 0.075% saponin (Sigma Aldrich # 47036-50G-F) and 100 nM Sytox (ThermoFisher Scientific #S7020) in 1xDPBS containing Mg^2+^ and Ca2^+^] adapted from [17]. The stained cells were imaged and counted with the IncuCyte^®^ S3 system and the experiment was repeated three times. When the effect of the TGFβRI/II inhibitor on spheroids was studied, 3D overlay media containing either 1 µM LY2109761 (Selleckchem #S2704) or 0.0025% DMSO vehicle was changed every other day, and spheroids were imaged six days after seeding the cells.

### Knocking down gene expression by short interfering RNAs (siRNAs)

Endoribonuclease-prepared siRNAs (esiRNAs) targeting *PALB2* (#EHU048861), *KRT14* (#EHU104641), and *EGFP* (#EHUEGFP) as a negative control, were purchased from Sigma-Aldrich. They were tested and transfected into cells at 2.5–10 nM concentration, 24 h after seeding the cells on 12-, 24- or 48-well plates, using of INTERFERin^®^ or MISSION^®^ siRNA transfection reagent (Polyplus #101000036, Sigma-Aldrich #S1452) in serum-free MCF10A media following the manufacturers’ protocol. 48 h after the transfection the cells were either lysed for Western blot analyses or detached and quantified using Life Technologies Countess II automatic cell counter (RRID:SCR_020236) to measure their viability. The quantified cells were used for RNA extraction, Transwell^®^ migration assays and/or setting up 3D cell culture plates. esiRNA KD efficiency was assessed by qRT-PCR and Western blot analyses as described in Supplementary Methods. The spheroids were re-transfected 4 days after 3D set up using normal MCF10A 3D overlay media containing 2% horse serum [15] as serum has been found to promote siRNA transfection into 3D-cultured cells [18]. The spheroids were imaged on day 5 or 6 *i.e.*, 7 or 8 days after the first transfection.

### Migration and invasion assays

One hundred thousand non-treated or 50 000 esiRNA-treated cells in MCF10A assay media [15] were seeded onto Transwell^®^ migration inserts (Corning^®^ #3464) or GFR-BME-coated BioCoat™ Matrigel^®^ invasion inserts (Corning^®^ #354483), following the manufacturer’s instructions. Invasion assays with reconstituted basement membrane method were carried out following the protocol by Hall and Brooks [19], using 1 mg Matrigel^®^/ml (Corning^®^ #354230). The cells were incubated for 20 h with complete MCF10A media in lower chambers as chemoattractant. To test the effect of TGFβRI/II inhibitor on migration, both assay and complete media were supplemented with either 1 µM LY2109761 (Selleckchem #S2704) or 0.0025% DMSO vehicle. Migrated or invaded cells were fixed with 70% ethanol and nuclei were stained with Hoechst^®^ 33342 (Thermo Scientific #10150888) before imaging.

### Imaging and image analysis

Immunofluorescence and DAPI/Hoechst -fluorescence images were captured using Zeiss LSM 780 laser scanning confocal microscope (Carl Zeiss) with Plan-Apochromat 40×/1.4 DIC or 20×/0.8 DIC objective and Zeiss Zen 2011 Black software (RRID:SCR_018163). Mitotic aberrations, migrated and invaded cells, KRT14-positive and KRT14-negative cells as well as part of γH2AX foci were counted utilizing Fiji ImageJ software (RRID:SCR_002285). Most DNA repair foci were quantified using the automated AKLIDES^®^ cell damage system (Medipan GmbH) at Universitätklinikum Hamburg-Eppendorf (Germany). Bright-field images of non-treated and DMSO/LY2109761-treated spheroids were captured using Zeiss Cell Observer spinning disc confocal microscope (Carl Zeiss) with Plan NeoFluar 10×/0.30 Ph1 objective and Zen 2012 Blue software (RRID:SCR_013672). Bright-field images of monolayer cells and esiRNA-treated spheroids were captured with VisiCam TC 20 mounted into Nicon Eclipse TE200 microscope. Spheroid images were analyzed with ImageJ.

### DNA fiber assay

Cells grown overnight were handled for analysis as described previously [20]. The cells were first pulse-labeled with 5′-chlorodeoxyuridine (CldU) and then with 5′-iododeoxyuridine (IdU), both for 30 min, followed by treatment with 4 mM hydroxy urea (HU) for 3 h. Replication tracks were imaged using a Deltavision workstation.

### Statistics

Statistical tests were performed using GraphPad Prism 8.4.3 software (RRID:SCR_002798) and *p*-values < 0.05 were considered significant. Data was first analyzed with D'Agostino-Pearson and Shapiro–Wilk normality tests and if passed, two-sided one-way ANOVA and Tukey’s multiple comparison test were used (Fig. 5a). If data did not pass the normality test, it was analyzed with Kruskal–Wallis test with Dunn’s multiple comparison post-test (Figs. 2b, 4e, 6a and b) or pairwise, two-tailed Mann–Whitney U tests (Figs. 6i, 7e and h). Differences between the cell lines in proliferation and LY210971 response (Figs. 4h and 6g) were tested using ordinary one-way Brown-Forsythe and Welch ANOVA for the areas under the curves. Statistical significance for the differences between proportions were determined with χ^2^ test using absolute numbers, followed by pairwise, two-tailed Fisher’s exact tests (Figs. 2c, d, 4b, c and 5b). The latter was also applied to test the differences between proportions in control and LY2109761- or esiRNA-KRT14 treated samples within each cell line (Figs. 6h, 7f and g). #Ctrl-45 and #Ctrl-2.52 were considered as one group in each statistical test where *PALB2*-mutated cells were compared to controls. The significance of the *p*-value derived from the tests is shown in each figure. Absolute numbers and ANOVA, Kruskal–Wallis or χ^2^ test results and the detailed statistical results are provided in Supplementary Table 2.

## Results

### The gene-edited MCF10A cell lines represent bi- and monoallelic *PALB2* defects

**Fig. 1:**
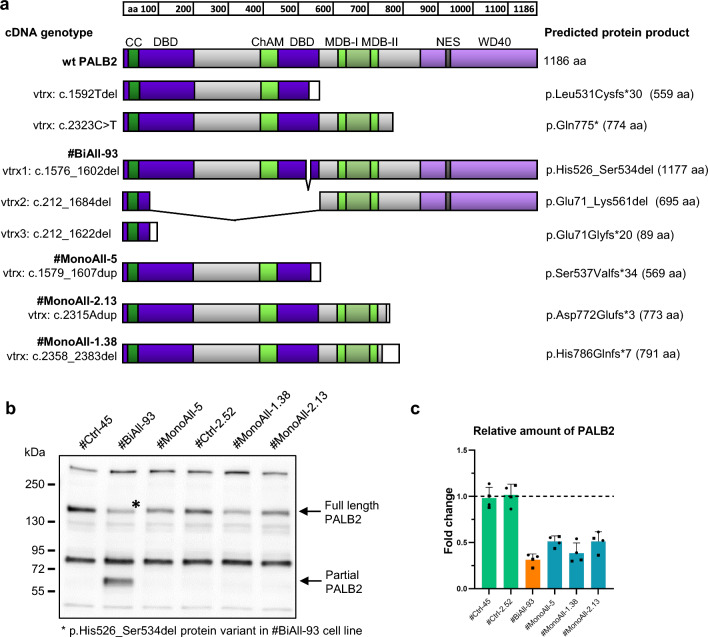
Non-cancerous human MCF10A mammary epithelial cells were modified by CRISPR/Cas9 gene editing to create bi- or monoallelically PALB2-compromised cell lines. **a** A schematic presentation of wt PALB2 protein and the expected protein products of two common pathogenic *PALB2* truncation founder variants, c.1592Tdel and c.2323C > T, and their comparison to gene-edited bi- and monoallelic *PALB2*-mutated cell lines based on cDNA sequencing. The #BiAll-93 cell line expressed altogether three different PALB2 transcripts, two of which showing in-frame deletions (c.1576_1602del, being almost full-length; the other, c.212_1684del, skipped exon four). The third transcript was a short out-of-frame variant (c.212_1622del). No wt PALB2 transcript was seen. #MonoAll-5, #MonoAll-2.13 and #MonoAll-1.38 had each a single mutation (c.1579_1607dup, c.2315Adup and c.2358_2383del, respectively), leading to a premature stop codon. The functional domains of PALB2 are marked with indicative colors on top of the wt protein. Aa, amino acids; CC, coiled-coil domain; DBD, DNA binding domain; ChAM, chromosome associated motif; MDB, MRG15 binding domain; NES, nuclear export signal; WD40, WD repeat domain; white domain, unspecific amino acids; vtrx, variant transcript. **b** A representative figure of Western blot analyses of PALB2 protein products using the M11 antibody in gene-edited *PALB2*-mutated and their control cell lines. Total protein staining of the membrane is given in Supplementary Fig. 2b. #BiAll-93 cells expressed a near-complete p.His526_Ser534del protein variant (*) and another partial protein product probably representing the p.Glu71_Lys561del variant or its derivative. A protein product from the third transcript variant in #BiAll-93 cells was not detected. The antibody recognized no truncated protein species in the monoallelically mutated cell lines. **c** Quantification of relative amount of full-length or almost full-length (in case of #BiAll-93) PALB2 protein based on Western blot analyses (*n* = 4) with M11 (circle) and E9RW2 (square) antibodies. The symbols depict replicates, bars mean values (± SD) and dashed line the mean of the two control cell lines

Mono- and biallelic *PALB2*-mutated MCF10A cell lines were generated using CRISPR/Cas9 gene-editing with guide-RNAs targeted to the fourth and fifth exons of *PALB2*. These procedures resulted in gene modification in regions adjacent to L531 and Q775 truncation and loss of PALB2 function mutations (Fig. 1a), the major clinically important pathogenic founder variants in the Finnish and Canadian population, respectively [21, 22]. The control cell lines with intact *PALB2* in both alleles were obtained alongside the same gene-editing procedures.

Only few hypomorphic biallelic *PALB2*-mutated cell lines were obtained as most of them did not survive the clonal expansion. Typically, the survived biallelically mutated cell lines, such as #BiAll-93, had a short in-frame deletion in at least one of the two *PALB2* alleles, thus giving rise to an expressed protein product with intact N- and C-terminal sequences and likely partial functionality (Fig. 1a, b and Supplementary Fig. 2a–c). In agreement with previously obtained data [5, 23], this indicated that cell lines totally lacking PALB2 function would not be viable in non-cancerous conditions. Altogether, #BiAll-93 cells expressed three *PALB2* transcripts of which the first one produced the near-complete p.His526_Ser534del protein variant, the second one with predicted protein product p.Glu71_Lys561del variant, and the third out-of-frame transcript without detectable protein product (Fig. 1a, b and Supplementary Fig. 2b–d). Complete lack of intact *PALB2* was thus comparable to Fanconi anemia cells.

The monoallelic PALB2-deficient cell lines, such as the selected #MonoAll-5, #MonoAll-2.13 and #MonoAll-1.38, typically harbored truncating mutations (Fig. 1a). Mutated #MonoAll-5 *PALB2* was closest to clinical c.1392Tdel variant: the predicted protein products were p.Ser537Valfs*34 and p.Leu531Cysfs*30, respectively, and even 30 of the out-of-frame amino acid residues in the end were the same. Mutated #MonoAll-2.13 *PALB2* again was very similar to the c.2323C > T variant: the predicted protein products being p.Asp772Glufs*3 and p.Gln775*. It should be noted that the wild type transcript was the dominant species in all of the three monoallelically *PALB2*-mutated cell lines. Only remnants of the mutated cDNA was detected, and no truncated protein with the anticipated length was seen (Fig. 1a, b and Supplementary Fig. 2b–c, e). The generated monoallelically deficient cell lines exhibited half dosage of the wild type PALB2 protein or less (Fig. 1c) and thus simulated the pre-cancerous mammary epithelial cells of carriers of commonly occurring and clinically relevant truncating *PALB2* mutations.

### Spheroids derived from *PALB2*-compromised cells are disarranged and their transcriptome profiles diverge from that of control cell spheroids

**Fig. 2:**
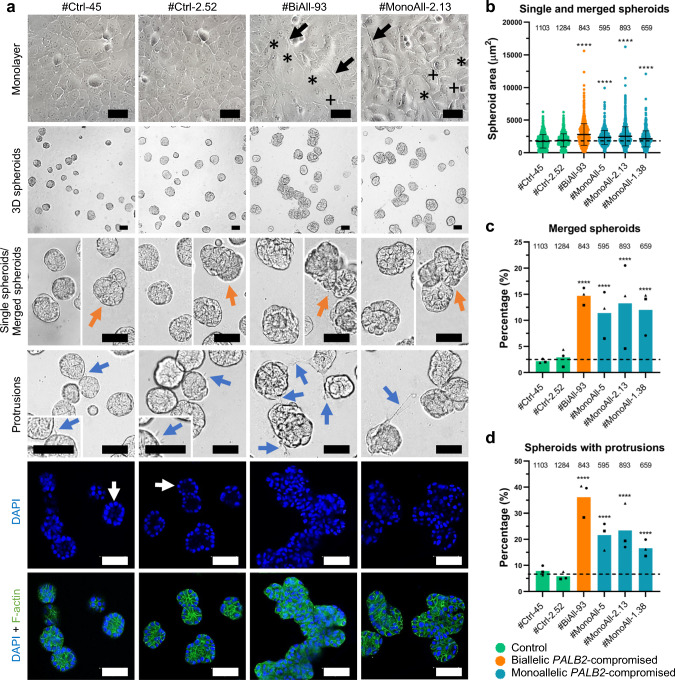
PALB2-compromised cells present disturbed monolayer morphology and spheroid formation. **a** Representative light (low- and high-magnification) and fluorescence microscopy images of control cell lines #Ctrl-45 and #Ctrl-2.52, and bi- and monoallelic *PALB2*-mutated cell lines #BiAll-93 and #MonoAll-2.13. In monolayer culture (1st row), control cell lines presented the typical cuboidal appearance of MCF10A cells, while a large number of *PALB2*-mutated cells had vacuolized cytosol (plus symbol), cytosol protrusions (black arrow) and/or senescence-like large and flat appearance with single or several nuclei (asterisks). In 3D culture (2nd to 6th row), control cells formed regular round spheroids consisting of a single cell layer (blue DAPI staining, white arrows). In contrast, PALB2-compromised spheroids were larger in size, more abundantly merged (orange arrows) and had more distinctive protrusions (blue arrows) than control cell-derived spheroids. Fluorescence images of equatorial cross sections of spheroids stained with DAPI (blue) and Alexa Fluor 488 Phalloidin (green) show nuclei and cell boundaries, respectively. Scale bars, 50 µm. **b** Quantification of cross-sectional areas (µm^2^) of single and merged spheroids in control cell lines, #BiAll-93 and three monoallelic PALB2-deficient cell lines demonstrated that the *PALB2*-mutated spheroids were larger than the control ones. The dots in the scatter dot plots represent individual spheroids. Horizontal lines designate mean values (± SD). **c** and** d** Merged spheroids (**c**) and spheroids with protrusions (**d**) are abundant in *PALB2*-mutated samples. The bars represent the proportion (%) of merged spheroids and spheroids with protrusions as a mean of three independent plates. Circle, plate 1; square, plate 2; triangle, plate 3. **a‒d** Spheroids were grown as triplicates and at least six images from different parts of each plate were randomly captured for data collection. **b-d** The total number of analyzed spheroids is given on top of each bar or plot and dashed lines show the mean of the two control cell lines. Statistical details are given in Supplementary Table 2a. SD, standard deviation; ****p < 0.0001

PALB2-compromised cell lines were characterized by an increased number of stressed cells having vacuolized cytosol and senescence-like flat appearance (Fig. 2a and Supplementary Fig. 3a). Indicative of senescence, the large and high-granularity cells accumulated beta-galactosidase (Supplementary Fig. 3b), and they were frequently multinuclear (Fig. 2a, Supplementary Fig. 3a). The spheroids derived from the *PALB2*-mutated cells pointed to malignant transformation of the cells: they formed multilayer non-organized structures that easily assembled, and several of the PALB2-compromised spheroids had distinctive protrusions towards each other and the surrounding matrix (Fig. 2a–d and Supplementary Fig. 3a, c–e), while the control cells formed round and polarized, acinus-like spheroids. To unveil mechanisms behind these morphological irregularities and other functional aberrations in PALB2-compromised cells, transcriptome profiling of #BiAll-93 and #MonoAll-2.13 cells and their controls was carried out from total RNA extracted from the spheroid samples.

**Fig. 3:**
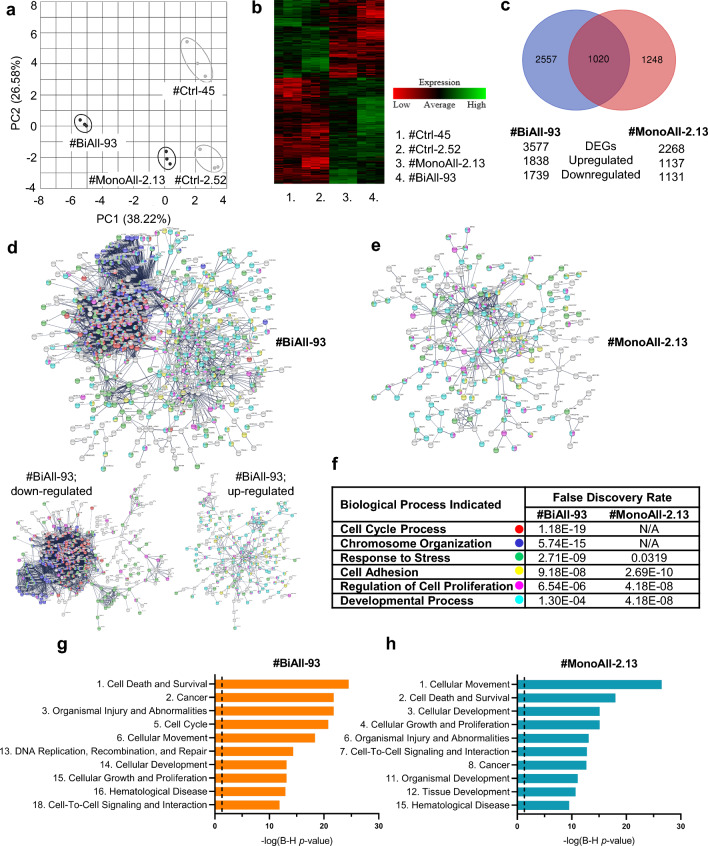
The gene expression profiles of *PALB2*-mutated spheroids have widely changed, and they associate with multiple cancer-related functions. **a** Principal component analysis of transcriptome data processed with DeSeq2 differential gene expression analysis showing deviation of the *PALB2*-mutated cell lines from the control ones. **b** Heat map plot of genes with a significant *q*-value (< 0.05) for the gene expression difference between *PALB2*-mutated and control cell lines.** c** Venn diagram of differentially expressed genes (DEGs) (*q* < 0.05) in #BiAll-93 and #MonoAll-2.13 spheroids compared to their isogenic control cell lines. **d** and** e** Clustering of the filtered DEGs (|L2fc |> 0.8) in #BiAll-93 cells (**d**), and with down- and up-regulated DEGs separately, as well as #MonoAll-2.13 cells (**e**) by STRING analysis package. Protein products have been colored with symbols according to the biological processes they are related to: cell cycle processes (red), chromosome organization (blue), response to stress (green), cell adhesion (yellow), regulation of cell population proliferation (magenta), and developmental processes (turqoise). Clustered genes that do not belong to any of the above-mentioned biological processes have a white symbol. Disconnected nodes have been hidden in the network. Down- and up-regulated DEGs have been scaled down in comparison to the combined analysis. **f** Four out of six important biological processes that have changed in #BiAll-93 are also altered in #MonoAll-2.13. The three most significant and independent processes according to STRING analysis have been listed separately for #BiAll-93 and #MonoAll-2.13 cell lines. N/A, non applicable. **g** and** h** Selected top diseases and functions related to DEGs in #BiAll-93 (**g**) and #MonoAll-2.13 (**h**) spheroids, according to Ingenuity Pathway Analysis (IPA). The complete lists are shown in Supplementary Table 5. **f–h** False discovery rate *p*-values have been corrected using the Benjamini–Hochberg procedure. Dashed line in **g** and **h** indicates the threshold for a significant B–H *p*-value < 0.05 [− log(B–H *p*-value) > 1.3]

Principal component and heatmap analyses of the obtained transcriptome sequencing data demonstrated that both types of *PALB2*-mutated cell lines clustered apart from the control samples, and #BiAll-93 more prominently than #MonoAll-2.13 (Fig. 3a, b and Supplementary Fig. 4a, b). Lists of differentially expressed genes (DEGs) were generated by comparing the transcriptomes of #BiAll-93 and #MonoAll-2.13 to their concurrently produced control cell lines #Ctrl-45 and #Ctrl-2.52, respectively, and as assumed, the number of DEGs was notably higher in #BiAll-93 than in #MonoAll-2.13 (Fig. 3c and Supplementary Table 1a and b). The expression patterns of the selected genes in the two other monoallelic *PALB2*-deficient cell lines, #MonoAll-5 and #MonoAll-1.38, also largely followed that of #MonoAll-2.13 (Supplementary Table 3). This further reflected the similarity between the different monoallelic *PALB2*-deficient cell lines.

### The most apparent transcriptome alterations in biallelically *PALB2*-mutated cells are associated with DDR, but the DEGs shared by all *PALB2*-mutated cells also indicate transformations beyond DDR

The DEG lists of #BiAll-93 and #MonoAll-2.13 were first analyzed with the STRING software package to visualize which cellular networks the genes belonged to. The protein–protein interaction enrichment was highly significant (*p*-values < 1.0 × 10^–16^) for both #BiAll-93 and #MonoAll-2.13 (Supplementary Table 4a, b), and most of the proteins grouping together were linked to six distinct biological functions (Fig. 3d–f). #BiAll-93 and #MonoAll-2.13 cells shared a cluster, representing the combination of four Gene Ontology (GO) terms that were associated to stress response, cell adhesion and proliferation, and processes of cellular and organismal development. The most significant protein networks formed by #BiAll-93 DEGs only were associated with regulation of cell division and maintenance of genome integrity, and these altered functions were mostly affiliated with down-regulated genes (Fig. 3d).

Secondly, the DEG lists weighed with the calculated expression differences (*i.e.*, L2fc values) were entered into the Ingenuity Pathway Analysis (IPA) application to identify what downstream effects the *PALB2*-mutated cell line DEGs were associated with. “*Cancer*” and related categories such as “*Cell death and survival*”, “*Cellular movement*”, “*Cellular growth and proliferation*”, as well as “*Cell-to-cell signaling and interactions*” were among the top altered functions identified in both bi- and monoallelically *PALB2*-deficient cell lines (Fig. 3g, h and Supplementary Table 5a–c). The DEGs in #BiAll-93 and #MonoAll-2.13 were also associated with the development and dysfunction of several organs, in keeping with the data from mouse that homozygous loss of *Palb2* is embryonically lethal [23]. Again, cell cycle processes including DNA replication, recombination, and repair including hereditary breast cancer signaling, were on top of the lists of diseases and functions and canonical pathways associated with the #BiAll-93 DEGs only (Fig. 3g, Table 1 and Supplementary Table 5, 6). It is also noteworthy that signaling in other epithelial cancers, such as in pancreatic, colorectal and ovarian carcinomas, were among those with the highest, activating z-scores on the list of top DEG-associated canonical pathways (Table 1, Supplementary Table 6a). Comparison of the significant top canonical pathways [− log(B–H *p*-value) > 1.3, or activity z-score < − 2 or > 2] of #BiAll-93 and #MonoAll-2.13 resulted in 15 shared pathways (Supplementary Table 6), further suggesting that both monoallelic and biallelic *PALB2*-mutated cell lines had gained similar oncogenic and also other altered properties.

**Table 1:** Selected relevant top canonical pathways associated with DEGs in #BiAll-93 and #MonoAll-2.13 cells with − log(B–H *p*-value) > 1.3 or *z*-score ≤ − 2.0 or ≥ 2.0

	#BiAll-93		#MonoAll-2.13	
Ingenuity canonical pathways	– log(B–H *p*-value^a^)	z-score	– log(B–H *p*-value)	z-score
Cell cycle control of chromosomal replication	6.95E+00	N/A^b^	N/A2^c^	
Cell cycle: G2/M DNA damage checkpoint regulation	5.02E+00	0.943	N/A2	
Mitotic roles of Polo-like kinase	4.76E+00	− 1.604	N/A2	
Role of CHK proteins in cell cycle checkpoint control	4.27E+00	1.069	N/A2	
Role of BRCA1 in DNA damage response	4.26E+00	− 2.668	N/A2	
Hereditary breast cancer signaling	3.99E+00	N/A	N/A2	
ATM signaling	3.38E+00	− 1.091	N/A2	
NER pathway	2.57E+00	− 1.147	N/A2	
P53 signaling	2.49E+00	0	1.37E+00	0.632
Molecular mechanisms of cancer	2.38E+00	N/A	1.29E+00	N/A
GADD45 signaling	2.20E+00	N/A	N/A2	
DNA damage-induced 14-3-3σ signaling	2.20E+00	N/A	N/A2	
Cyclins and cell cycle regulation	1.99E+00	− 2.840	N/A2	
Mismatch repair in eukaryotes	1.99E+00	N/A	N/A2	
Colorectal cancer metastasis signaling	1.44E+00	2.475	N/A2	
Pancreatic adenocarcinoma signaling	1.05E+00	3.051	N/A2	
GP6 signaling pathway	7.42E−01	2.065	5.49E+00	1.732
Ovarian cancer signaling	6.14E−01	2.111	1.35E+00	0.302

Complete data is presented in Supplementary Table 6

^a^B–H *p*-value, Benjamini–Hochberg-adjusted *p*-value

^b^N/A, z-score is zero and prediction of activation or inactivation cannot be done

^c^N/A2, neither − log(B–H *p*-value) was > 1.3, nor z-score < -2 or > 2

### *PALB2*-compromised cell lines demonstrate defective DNA damage response and p53 activation, both of which also predicted by the alterations observed in the #BiAll-93 transcriptome profile

It is well documented that several primary processes in the DDR are fast and post-translationally regulated, but also slower transcriptional responses are mediating damage information within a cell and participate in maintaining a biologically sufficient response [24].

**Fig. 4:**
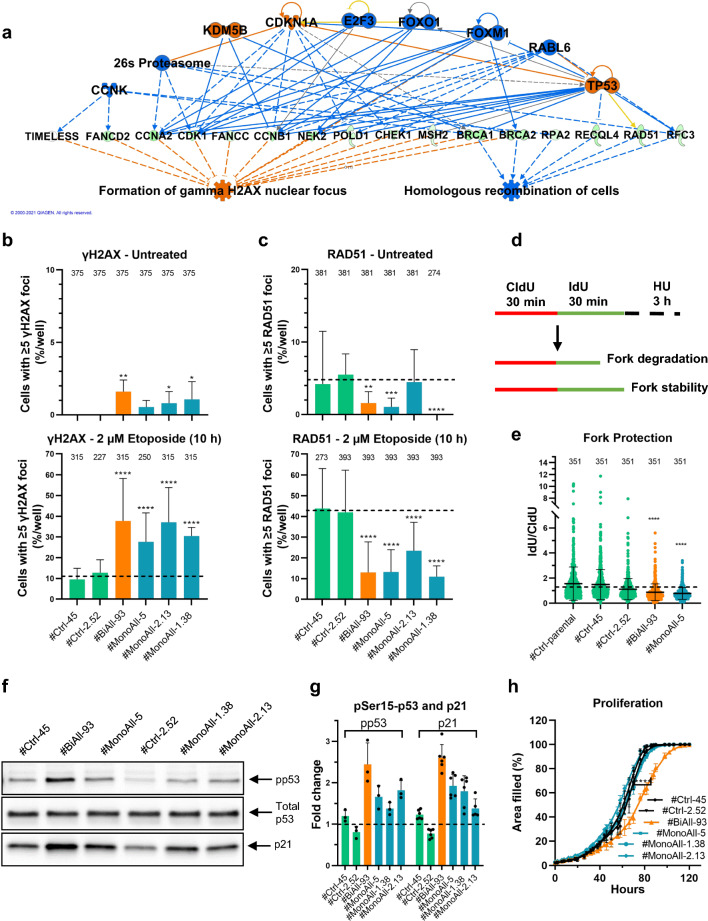
DNA damage has increased, and its repair decreased in PALB2-compromised cell lines, along with p53 activation. **a** IPA regulator effect network with the highest consistency score (13.250) for the biallelic *PALB2* mutant cell line showing predicted upstream regulators and phenotypic and functional outcomes related to the associated DEGs. All DEGs with L2fc ≤ − 0.5 or ≥ 0.5 with their L2fc-values have been used in the analysis, and those connected with the network are shown in the diagram. Orange and blue symbols depict activation and inactivation, respectively, green symbols decreased transcription, orange and blue lines signaling leading to activation and inhibition, respectively, and yellow and grey lines indicate that the findings are inconsistent with state of downstream molecule or that the effect is not predicted. **b** Proportion of cells with ≥ five foci of DNA damage marker γH2AX per analyzed well (*n* = 3) in control and PALB2-compromised cells without (upper graph) and with (lower graph) 10 h 2 µM etoposide treatment. **c** Percentage of cells with ≥ five foci of homologous recombination marker RAD51 per analyzed well (*n* = 3) in control and PALB2-compromised cells without (upper graph) and with (lower graph) 10 h 2 µM etoposide treatment. **d** Experimental setup to probe replication fork stability and protection. **e** Fork protection in control and selected PALB2-compromised cell lines presented as IdU/CldU ratio after 4 mM hydroxyurea (HU) treatment. Corresponding data for parental MCF10A cells (#Ctrl-parental) is also shown. **b**, **c**, **e** The analyses were performed three times. The bars and horizontal lines in scatter dot plots designate the mean values (± SD). The total number of counted nuclei (**b**, **c**) and measured fibers from triplicates (**e**) is shown on top of each bar or plot. Dashed lines represent the mean of #Ctrl-45 and #Ctrl-2-52. SD, standard deviation; CldU, 5′-chlorodeoxyuridine; IdU, 5′-iododeoxyuridine. **f** Representative images showing Ser15-phosphorylation of p53 (pp53) and amount of p21 in PALB2-compromised cells. The whole membrane pieces and their total protein staining are shown in Supplementary Fig. 6a. **g** Quantification of pSer15-p53 and p21 based on Western blot membranes. The symbols depict biological replicates (*n* = 3–6). **h** Proliferation capacity of *PALB2*-mutated and control cell lines measured by the well area filled by the cells. A symbol and vertical line demonstrate mean and standard deviation of four replicates and the asterisks mark the observed difference between the average of control cells and #BiAll-93 cells. Statistical details are given in Supplementary Table 2b, c. *p < 0.05, ***p < 0.001, ****p < 0.0001

To elucidate how the inadequate PALB2 function and consequent cellular phenotypes were linked together, IPA was used to predict potential upstream regulators of the identified DEGs and they were integrated with the downstream cellular outcomes to generate predictive regulatory networks. Indeed, underlining the importance of PALB2 in DDR, the regulatory network with the highest consistency score for #BiAll-93 predicted increased formation of γH2AX foci, a marker of DNA damage and decreased homologous recombination (Fig. 4a and Supplementary Table 7). In agreement, gain of γH2AX nuclear foci formation and reduction in RAD51 recombinase staining were detected both before and after DNA-damaging etoposide treatment. Both the number of cells with five or more γH2AX or RAD51 foci (Fig. 4b–c) and also their total foci number (Supplementary Fig. 5a–f) had significantly changed in #BiAll-93 and most of the monoallelically *PALB2*-mutated samples. Increased genotoxic stress was also demonstrated, with increased foci formation of the p53 binding protein 1 (53BP1) (Supplementary Fig. 5 g, h) that accumulates at damaged chromatin and recruits other double-strand break repair proteins at the site [25], and with defective replication fork protection. The 5′-iododeoxyuridine/5′-chlorodeoxyuridine (IdU/CldU) ratio was reduced in PALB2-compromised cells after hydroxy urea treatment in fiber assays, indicating faulty fork protection (Fig. 4d, e). The congruence of the results with the known functions of PALB2 thus further validated the gene-edited cell lines for the studies of PALB2-deficiency. The above results also emphasized that the level of spontaneous and induced DNA damage had increased and their repair decreased in the monoallelic *PALB2-*defective cell lines too, though the defects were mostly less substantial and less evidently recognized by IPA than in the biallelic cell line (Fig. 4b, c and Supplementary Fig. 5).

P53 is one of the best characterized factors mediating DDR signal via transcription [24, 26]. Consistent with the increased DNA damage in PALB2-compromised cells, IPA identified p53 as plausible upstream regulator of the DEGs in #BiAll-93 cells in the above-mentioned (Fig. 4a) and other regulatory networks with a very high bias-corrected activation z-score of 6.79 (Supplementary Table 8a). Activation of p53 in PALB2-compromised cells was substantiated with enhanced phosphorylation of p53 at Ser15 (Fig. 4f, g and Supplementary Fig. 6a), which is a major site of phosphorylation after DNA damage [27]. Consequently, the amount of p21 (gene alias *CDKN1A*), a target of p53 had increased most in #BiAll-93 cells (Fig. 4f, g). Although the increase was only up to 2.5-fold in the PALB2-compromised cells, in #BiAll-93 cells it roughly corresponded to that observed in control cells after treatment with a continuous low-level dosage of etoposide (Supplementary Fig. 5i) and such treatment was sufficient to cause DNA damage to the cells (Supplementary Fig. 5j, k). In line with p53 activation and function, downstream targets, such as the mitosis-promoting *FOXM1*, *CCNB1* and *CCNB2,* were also repressed in #BiAll-93 cells (Supplementary Table 1a) and congruently, the proliferation of #BiAll-93 cells had slowed down (Fig. 4h). An increase in cellular senescence is a general consequence of p53 activation, and this was also recognized to occur by IPA (Supplementary Table 7) and was readily observed in the *PALB2*-mutated cells (Fig. 2a and Supplementary Fig. 3a, b). While senescence is one of the characteristics of Fanconi anemia cells [28] additional alterations in the transcriptome profile of #BiAll-93 suggested a connection to hematopoietic abnormalities that are often seen in this disease (Supplementary Table 7).

### Various cell division errors appear abundantly in *PALB2*-compromised cell lines

**Fig. 5:**
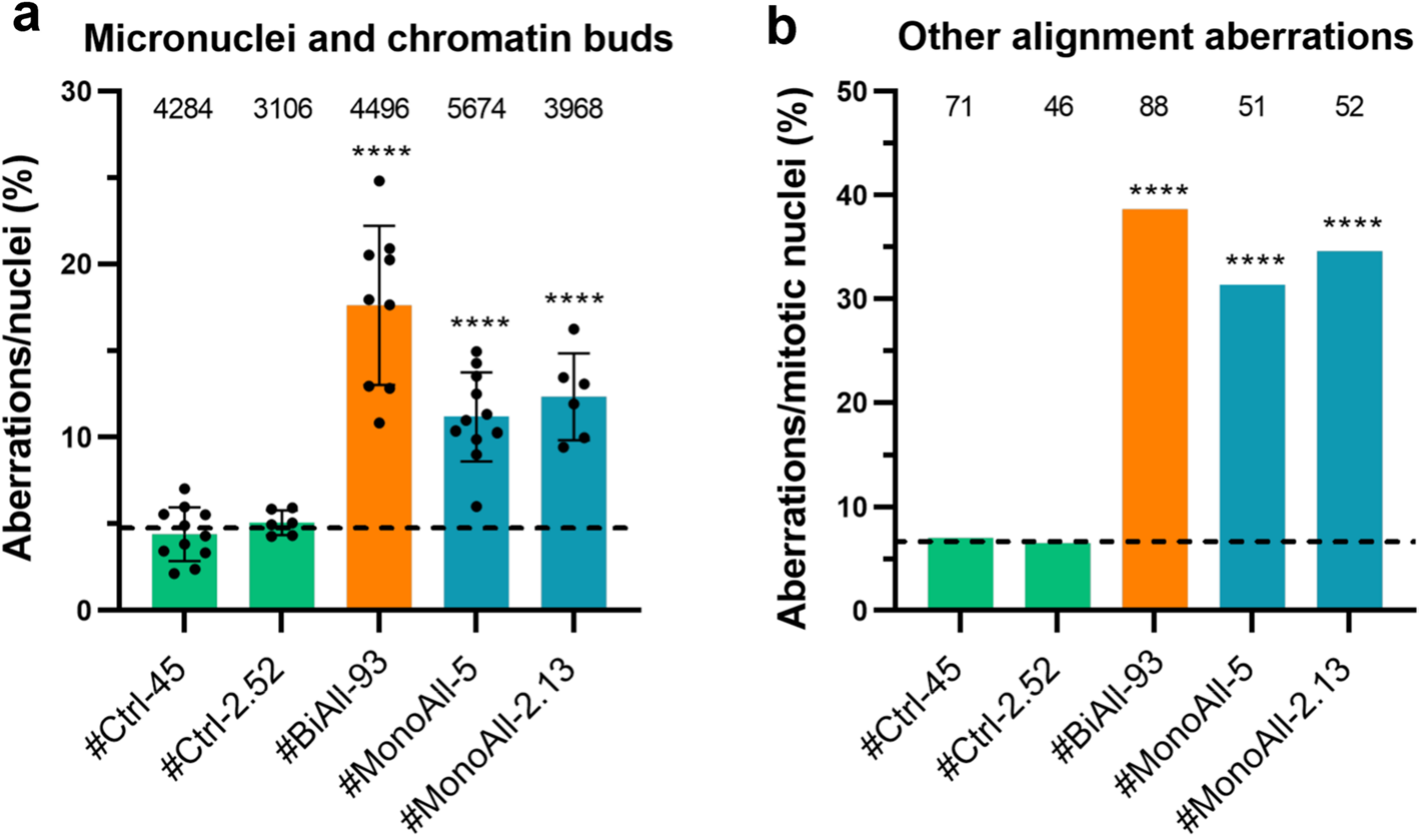
The level of chromosomal aberrations is increased in *PALB2*-compromized cells. **a** Bar chart showing percentage of micronuclei and chromatin buds per all nuclei in control and PALB2-compromised cells. The circle symbols represent experimental repeats (*n* = 6–11) and the total number of counted nuclei pooled from the repeats are shown on top of each bar. The bars designate the mean values (± SD). Dashed lines represent the mean of #Ctrl-45 and #Ctrl-2-52. **b** Number of pooled misalignment features excluding micronuclei and chromatin buds, *i.e.* chromatin bridges, lagging chromosomes, and asymmetric cell divisions, in ratio to number of mitotic nuclei. Aberrations were combined from 3 (#Ctrl-2.52 and #MonoAll-2.13), 7 (#BiAll-93), or 9 (#Ctrl-45 and #MonoAll-5) experimental replicates, and the total count of mitotic nuclei in the replicates has been given on top of each bar. Statistical details are given in Supplementary Table 2d. ****p < 0.0001

The IPA network with the second highest consistency score for #BiAll-93 predicted a decrease in chromosome alignment and chromosomal congression (Supplementary Fig. 7a). The DEGs on which the prediction was based included several down-regulated members of the kinesin superfamily and mitosis regulators that are essential for centromere separation and maturation, mitosis spindle assembly, and maintaining of mitotic checkpoints (Supplementary Table 4c). Again, both biallelic and monoallelic *PALB2*-mutated cells presented an increased number of features of misalignment and DNA misamplification: micronuclei, chromatin buds, lagging chromosomes, anaphase and nucleoplasmic bridges, and asymmetric cell divisions (Fig. 5 and Supplementary Fig. 7b). The number of micronuclei and chromatin buds was substantial already in early-passage *PALB2*-mutated cells and did not increase further in the aged cell lines (Supplementary Fig. 7c), indicating that accumulation of additional mutations was not necessary to trigger their formation.

### Aberrations in *PALB2*-mutated cells include an increase in migratory capacity

**Fig. 6:**
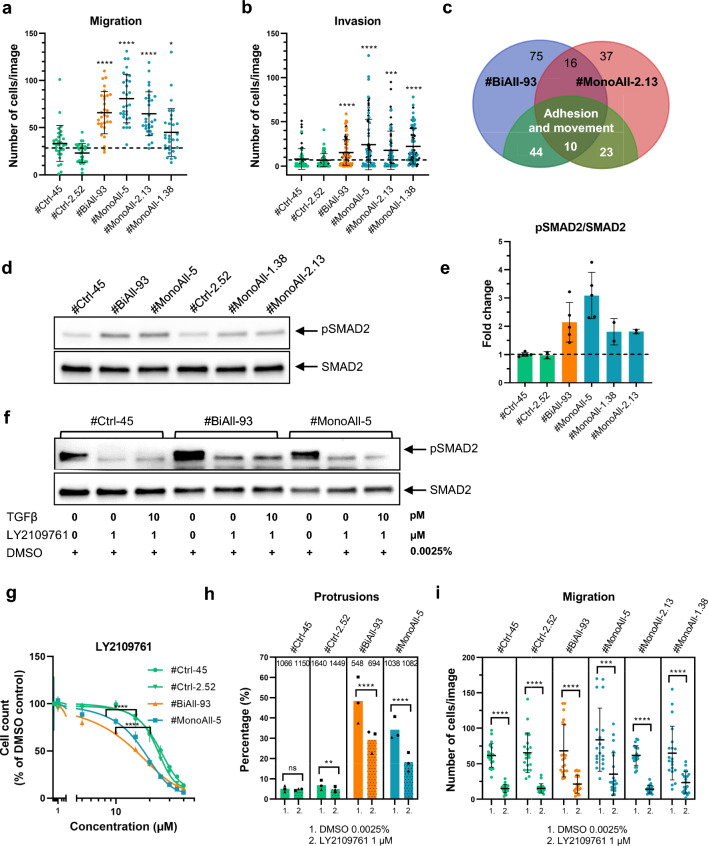
*PALB2*-mutated cells have enhanced ability to migrate and invade, and that is dependent on enhanced TGFβ signaling. **a** and **b** Scatter dot plots showing migration (**a**) and invasion (**b**) of control and *PALB2*-mutated cells through Transwell^®^ membranes. Each dot symbolizes the number of migrated or invaded cells per one image (area = 0.18 mm.^2^). Ten images from three (**a**) or eight (**b**) independent replicates (*n* = 10 × 3 or 10 × 8) per cell line were randomly captured from different parts of the membranes with fixed migrated or invaded cells. In **b** solid color-coded circles and black diamonds demonstrate results obtained from Transwell^®^ inserts with reconstituted Matrigel^®^ and BioCoat™ Matrigel^®^ invasion inserts, respectively. Representative images of migration and invasion membranes are shown in Supplementary Fig. 8. **c** Venn diagram of the most differentially expressed genes (DEGs, L2fc ≤ − 2.0 or ≥ 2.0) in *PALB2*-mutated cell lines #BiAll-93 (blue) and #MonoAll-2.13 (red) and the most differentially expressed genes related to cell adhesion, migration and interaction (green). **d** A representative image of amount of Ser465/467-phosphorylated SMAD2 (pSMAD2) and total SMAD2 in *PALB2*-mutated cell lines and (**e)** quantification of their ratio from Western blots. The dot symbols depict biological replicates (*n* = 2–5). **f** Inhibition of pSMAD2 by TGFβ receptor I/II inhibitor LY2109761 (long exposure) after 24 h of treatment. The whole membrane pieces and their total protein staining are shown in Supplementary Fig. 6b. **g** Sensitivity of control and *PALB2*-mutated cell lines to LY2109761. The symbols depict the average of triplicates ± SD. **h** Proportion (%) of spheroids with protrusions in 3D-cultured control and PALB2-compromised cells treated with 0.0025% DMSO as vehicle control (plain bars) or 1 µM LY2109761 (dotted bars). Spheroids were grown as triplicates and ten images from different parts of each plate were randomly captured for data collection. The bars represent the mean of the triplicates and the total number of analyzed spheroids is given on top of each bar. Circle, plate 1; square, plate 2; triangle, plate 3. **i** Number of migrated cells per image in control and PALB2-compromised cells treated with 0.0025% DMSO or 1 µM LY2109761. Ten images from two replicates (*n* = 10 × 2) per cell line and treatment were randomly captured. Migration ability of the samples with DMSO vehicle is not comparable to the samples without DMSO in **a**, since DMSO as such tends to increase migratory capacity of control cell lines but decreases it in *PALB2*-mutated cell lines (Supplementary Fig. 10d–e). **h** and **i** Representative images are shown in Supplementary Fig. 10a and b. Horizontal constant lines (**a**, **b**, **i**) or bars (**e**, **i**) designate the mean values (± SD) and dashed lines (**a**, **b**, **e)** show the mean of two control cell lines. Statistical details are given in Supplementary Table 2e, f. SD, standard deviation; ns, not significant; *p < 0.05, **p < 0.01, ***p < 0.001, and ****p < 0.0001

The appearance of PALB2-compromised spheroids and results from the transcriptome data analysis above were indicative of misregulation of the adhesive and migratory capacity of the cells. Experimental data confirmed that the number of migrated PALB2-compromised cells through Transwell^®^ membranes was significantly higher than that of the control cells (Fig. 6a and Supplementary Fig. 8a). All *PALB2* mutant cells also invaded through the extracellular matrix more efficiently than the control cells (Fig. 6b and Supplementary Fig. 8b).

The term “*Cell adhesion*” in STRING analysis, and terms “*Cellular movement”* and “*Cell-to-cell signaling and interaction*” in the IPA results, were associated to both #BiAll-93 and #MonoAll-2.13 DEG lists with low adjusted *p*-values (Supplementary Table 4, 5). It is noteworthy that the DEGs related to these three categories covered 37% and 38% of the most down- and up-regulated genes (L2fc < − 2 or > 2) in #BiAll-93 and #MonoAll-2.13 cells, respectively (Fig. 6c, Supplementary Table 1c).

### TGFβ signaling has been enhanced in *PALB2*-compromised cells and its inhibition partially normalizes the morphology and migratory capacity of the cells

IPA predicted altogether 243 and 143 putative upstream regulators (B–H corrected *p*-value ≤ 0.01, or activity z-score ≤ − 2 or ≥ 2) for DEGs in #BiAll-93 and #MonoAll-2.13 cells, respectively (Supplementary Table 8a, b). In addition to p53, the most significant predicted regulators included transcription factors/chromatin modulators and cytokines (Supplementary Table 8). Highly significant adjusted *p*-values indicated that TGFβ and tumor necrosis factor (TNF) were among the most likely upstream regulators of DEGs in both #BiAll-93 and #MonoAll-2.13 cells (Supplementary Table 8). TGFB1, TNF and their modulator and downstream effector nuclear factor kappa B (NFκB) were also central factors in the causal networks of functions, and in particular in those associated with cellular movement (Supplementary Fig. 9).

Here we focused on TGFβ signaling, since DDR is known to affect TGFβ pathways and vice versa [29, 30]. Phosphorylation of SMAD2, a main mediator of canonical TGFβ signaling was indeed increased two to three-fold in all PALB2-compromised cell lines (Fig. 6d, e) and that was counteracted by adding TGFβRI/II inhibitor, LY2109761 (Fig. 6f). The PALB2-compromised cell lines were also more sensitive to high concentration of this inhibitor than the control cell lines (Fig. 6g), pointing to their altered TGFβ response. Similarly, the exposure to a TGFβR inhibitor has been shown to decrease the survival of the MCF10A cells, into which DNA damage was induced with ionizing radiation [31]. Most importantly, inhibition of TGFβ-signaling noteworthily reduced protrusions in PALB2-compromised spheroids and also migration of the cells (Fig. 6h, i and Supplementary Fig. 10a, b), though not agglomeration of the spheroids (Supplementary Fig. 10c). Both the proportion of the *PALB2*-mutated spheroids with the protrusions and number of the mutated migrated cells decreased approximately 40–60% after LY2109761 treatment. The increased migratory capacity of *PALB2*-mutated cells was hence caused, at least partially by the enhanced TGFβ signaling.

### TGFβ alleviates DNA damage and cell division errors in #BiAll-93 cells

To investigate combined effects of DNA damage and TGFβ signaling on PALB2-compromised and their control cells, we next treated them with low-concentration etoposide and excessive (50 pM) TGFβ. 0.2 µM etoposide treatment for 48 h alone increased the number of γH2AX foci and cell division errors in #Ctrl-45 and #MonoAll-5 cells but could not significantly raise them further in #BiAll-93 cells (Supplementary Fig. 11). TGFβ could not lessen the influence of etoposide, and its effect on basal DNA damage in #Ctrl-45 and #MonoAll-5 cell lines was also insignificant. Instead, excessive TGFβ treatment reduced the number of γH2AX foci and chromosomal aberrations in #BiAll-93 cells. The number of all and larger than 0.4 µm^2^ foci decreased on average by 26% and 49% respectively (Supplementary Fig. 11a, b), suggesting that TGFβ had alleviated severe DNA damage, in particular. However, such TGFβ treatment was not able to compensate the defects caused by insufficient PALB2 function, but the number of γH2AX foci and cell division errors remained relatively high.

### *KRT14* is a target gene of TGFβ, its amount correlates with DNA damage, and its knock down partially restores *PALB2*-mutated cells

**Fig. 7:**
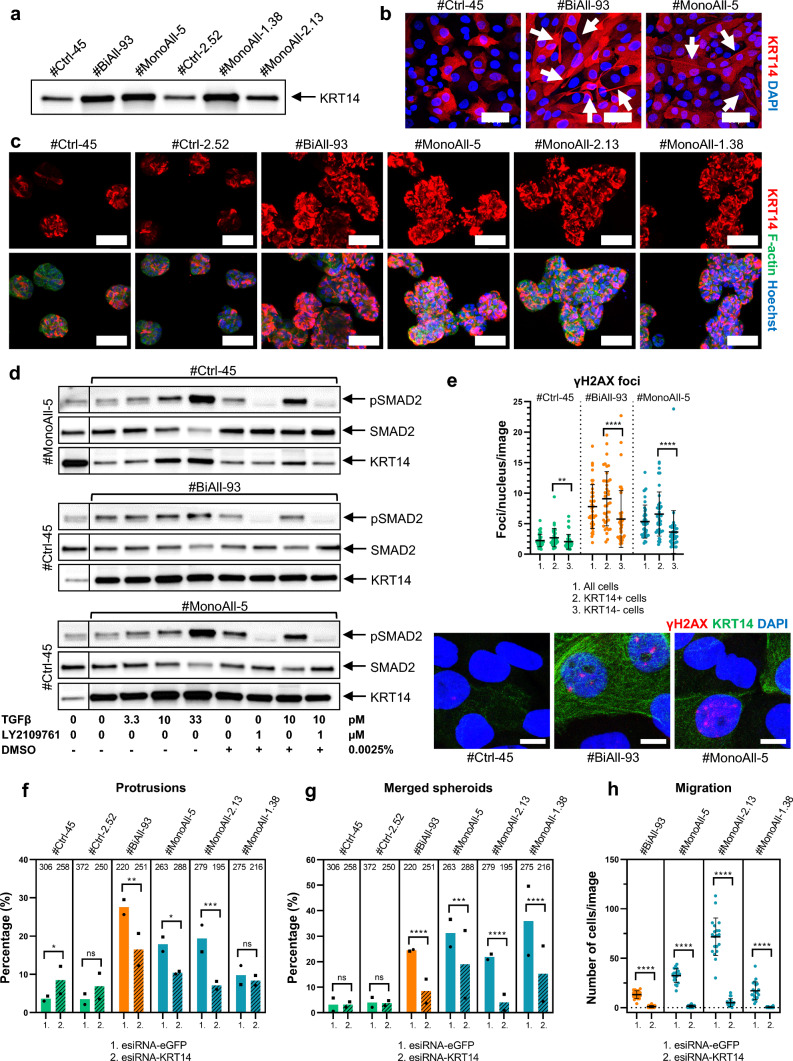
*KRT14* is upregulated in PALB2-compromised cells, its amount correlates with DNA damage, and its knockdown partially restores abnormal spheroid and migratory phenotypes.** a** A representative Western blot image showing KRT14 in *PALB2*-mutated and control cell lines.** b** Representative monolayer images of KRT14 (red) immunostaining in a control, and biallelic and monoallelic PALB2-compromised cell line. Intensive KRT14 staining is seen in cytoplasmic projections (arrows). Nuclei have been stained with DAPI (blue). **c** Representative maximum intensity projection images showing KRT14 (red) immunostaining alone (top row) and combined with phalloidin F-actin (green) and Hoechst nuclei (blue) staining (bottom row) in control and PALB2-compromised spheroids. Projection images were created out of Z-stack slices captured across the spheroids. **d** Up-regulation and inhibition of pSMAD2 (pSer465/467) and KRT14 by TGFβ and LY2109761, respectively in #Ctrl-45, BiAll-93 and MonoAll-5 cell lines after 24-h treatments. For comparison the first sample in a row is either from #MonoAll-5 or #Ctrl-45 cells as indicated. **e** Number of γH2AX foci in nuclei of KRT14-positive and KRT14-negative cells in different cell lines with representative figures below. Ten images, each including four z-stack slices, were randomly captured from four separate wells per cell line (*n* = 10 × 4). Each dot represents an image, and the horizontal lines designate mean values (± SD). **f** and **g** Proportion (%) of spheroids with protrusions (**f**) and merged spheroids (**g**) in 3D-cultured control and PALB2-compromised cells treated with 10 nM control eGFP-esiRNA (plain bars) or KRT14-esiRNA (striped bars). Spheroids were grown as duplicates and ten images from different parts of each plate were randomly captured at five or six days. The bars represent the mean of the duplicates and the total number of analyzed spheroids is given on top of each bar. Circle, plate 1 (5 days); square, plate 2 (6 days). **h** Migration of control and PALB2-compromised cells treated with 10 nM control eGFP-esiRNA or KRT14-esiRNA. Each dot symbolizes the number of migrated cells per one image (area = 0.18 mm^2^). Ten images from two replicates (*n* = 10 × 2) per cell line and treatment were randomly captured from different parts of the Transwell^®^ membranes with fixed migrated cells. Horizontal lines designate the mean values (± SD). **a**, **d** The whole membrane fragments and total protein staining are seen in Supplementary Fig. 6c. Representative images for **e–h** are shown in Supplementary Fig. 12d, 13a and e, respectively. Scale bars (**b, c**) 50 µm and (**e**) 10 µm. Statistical details are given in Supplementary Table 2 g and h. SD, standard deviation; ns, not significant; *p < 0.05, **p < 0.01, ***p < 0.001, ****p < 0.0001

*KRT14* was among the cell adhesion and motility-associated genes, the expression of which being significantly enhanced in both biallelically and monoallelically *PALB2*-mutated cell lines (Fig. 7a and Supplementary Table 1c and 3). In 2D-cultured *PALB2*-mutated cells KRT14 was noticeably seen in the cytoplasmic projections and in particular, *PALB2*-mutated spheroids were rich in KRT14 (Fig. 7b, c and Supplementary Fig. 12a). Interestingly, the gain of *KRT14* expression was higher in the spheroids than in monolayer cells (Supplementary Fig. 12b) demonstrating again that the extracellular environment modulates cellular function. The amount of KRT14 and phosphorylation of SMAD2 increased parallelly by TGFβ treatment, indicating that *KRT14* was a target gene of TGFβ signaling in the MCF10A cells (Fig. 7d, Supplementary Fig. 6). The KRT14 gain induced by TGFβ was the most obvious in #Ctrl-45 cells, while the expression was already so high in the mutant cell lines that it could not be elevated in the same ratio. Notably, nuclear γH2AX foci were more abundant in KRT14-positive than in KRT14-negative cells, particularly in PALB2-compromized cell lines (Fig. 7e, Supplementary Fig. 12d, e) that also contained more KRT14-positive cells (Supplementary Fig. 12f). Thus, there was a distinct positive correlation between DNA damage and *KRT14* expression.

Knocking down *KRT14* by esiRNA treatment significantly reduced the aberrations of PALB2-deficient spheroids; the number of protrusions (Fig. 7f and Supplementary Fig. 13a) and to some extent the size of the spheroids fell (Supplementary Fig. 13b). *KRT14* KD also decreased the agglomeration of the spheroids (Fig. 7g), while LY2109761 was unable to do that (Supplementary Fig. 10c). LY2109761 could also block only the effect of the additional TGFβ signaling and had less influence on the basal high expression of *KRT14* in PALB2-compromised cells (Fig. 7d, Supplementary Fig. 12c). Thus, while TGFβ could significantly upregulate *KRT14* expression, the gene also displayed apparent TGFβ-independent function.

In monolayer culture, MCF10A-derived cell lines were highly dependent on the amount of KRT14 and even its partial KD strongly reduced the viability of the cells (Supplementary Fig. 13c, d). The control cells with lowish *KRT14* expression were particularly distressed, making investigation of their migratory capacity after *KRT14* KD treatment challenging. The mutated cells, however, survived the treatment better and the remained cells had indeed reduced capability to migrate (Fig. 7h and Supplementary Fig. 13e), further indicating that *KRT14* was augmenting the migratory capacity of the *PALB2*-mutated cells.

## Discussion

Pathogenic *PALB2* germline variants frequently predispose their female carriers to breast carcinoma with a high disease penetrance. The tumors arising from *PALB2* mutations are typically luminal ones, but also the proportion of triple-negative breast cancers is higher among *PALB2* mutation carriers than in women with sporadic breast cancer [32, 33]. Approximately two-thirds of the germline *PALB2* mutation-associated tumors have somatically lost the function of the remaining wild type allele, and the resulting inactivation of both alleles has been found to be a requirement for homologous recombination deficiency (HRD) [34, 35], though in a studied cohort of *PALB2* mutation-associated tumors, HRD occurred similarly in both mono- and biallelic cases [2]. The requirement of appropriate PALB2 dosage and function has been well-recognized in the safeguarding of genomic stability and alleviation of replicative and oxidative stress, but the understanding of its input particularly in other important aspects of disease biology still remains obscure and incomplete. Here we have concentrated our efforts on the assessment of early cellular events triggered by either bi- or monoallelic *PALB2*-defects, corresponding to clinically important pathogenic variants, and explored how these events could lead to malignant transformation of the cells, and particularly on identifying novel outcomes disrupting vital functions beyond DNA damage response and repair.

Widely used patient-derived lymphoblastoid cells have offered a sound platform to study *PALB2* mutations in their own heterogenic backgrounds and have been an appropriate model for studying cell type-independent phenomena such as DDR [10, 36]. Human non-cancerous MCF10A cells again enabled the generation of isogenic cell lines and were more suitable for studying the overall initial consequences of PALB2 deficiency and in a mammary epithelial context. MCF10A cells have some characteristics of both luminal and basal epithelial cells, including the expression of *KRT8/18* and *KRT5/14* keratins, and they have been suggested to be the progenitor type of mammary epithelial cells [11]. Such cells are also considered as the candidate cells-of-origin in breast cancer [37]. In addition, MCF10A spheroids have been demonstrated to simulate the natural 3D structure of the mammalian duct and therefore to be an appropriate model to uncover the diversity and heterogeneity of premalignant in situ lesions within them [15, 38]. Finally, to avoid conclusions based on possible off-target mutations, we used various guide-RNAs during the gene-editing processes. As a result, we achieved parallel *PALB2*-mutated cell lines that mostly acted similarly, thus verifying the substance of the detected phenotypes. Most of the detected aberrations also showed gene dosage-sensitivity *i.e*. they were more severe in the #BiAll-93 than in the monoallelically mutated cell lines. Put together, we have generated a biologically relevant model for studying the cellular effects of permanent *i.e*., non-transient *PALB2* aberrations by gene editing the MCF10A cells.

As was expected, PALB2-compromised cells demonstrated typical features of cells with defective DNA damage response and homologous recombination. Like the cells with malfunctional BRCA1 and BRCA2 [39] and shown before [5, 7, 40], PALB2-compromised cells displayed accumulation of DNA breaks as well as decreased RAD51 foci formation, and consequently activation of p53. Such p53 activation, however, could neither completely arrest the proliferation of hypomorphic #BiAll-93 cells nor protect monoallelic *PALB2*-mutated cells from DNA damage and its repercussions. Increased endogenous DNA damage has also been demonstrated in hypomorphic *Palb2*^*CC6*^ mice predisposed to ionizing radiation, and continued cellular proliferation was observed despite p53 activation in them [40]. Like in the monoallelically *PALB2*-mutated MCF10A cells, parallel p53 activation and chromosomal aberrations have also been observed in luminal progenitor cells from healthy heterozygous *BRCA2* mutation carriers [41]. Together with previous results [10, 42], these findings further suggest that haploinsufficiency of *PALB2* or *BRCA2* can be a potential initiating event for malignant changes in these heterozygous cell models, and possibly also in cancers associated with such disease predisposition.

Unresolved DNA lesions cause intrinsic replication stress and vice versa [43], and they can lead to various mitotic segregation errors [44]. The phenomenon is linked to the incomplete function of several DNA replication and repair factors, including BRCA2 [41, 45] and PALB2 that is necessitated both in the repair of double-strand breaks and recruitment of Polη (gene alias *POLH*) at blocked replication forks [46]. Similarly to the PALB2-compromised cells here, increased replication stress has been observed in lymphoblastoid cells derived from heterozygous *PALB2* germline mutation carriers [10], thus being another example of aberrant DDR caused by *PALB2* haploinsufficiency. Previously it has also been shown that induced DNA damage or mild replication stress increased the formation of micronuclei and chromosomal aberrations in siRNA *PALB2* KD cells [47] and in cells expressing the MRG15 domain mutant PALB2 [48]. These chromosomal anomalies were obvious in both bi- and monoallelic *PALB2*-mutated cells at early cellular passages, and even in the absence of genotoxic agents. Altogether, the deficient amount of intact PALB2 can lead to intrinsic replication stress and mitotic aberrations.

Besides the obvious defects in DDR and replication, the PALB2-compromised cell lines demonstrated marked structural and functional aberrations such as protrusions in the spheroids and increased migratory capacity. These aberrations were, at least partially due to enhanced TGFβ signaling and increased KRT14 levels in the mutated cells. TGFβ is a pleiotropic cytokine and has wide context-specific, combined and diverse effects on cellular death, cytoskeletal function and migratory capacity [49]. TGFβ signaling is also connected with DNA damage and repair, and tumorigenesis at several levels. The complexity of the relationships is displayed by the data that TGFβ can both protect against and induce genomic instability. It can either increase or hinder expression of DDR gene expression, thus affecting the balance between error-free and error-prone DNA repair [29, 50–52]. The excessive amount of TGFβ could to some extent decrease the amount of DNA aberrations in the biallelically *PALB2*-mutated cells but could not compensate the effect of defective PALB2.

DDR factors such as BRCA1 and FANCA can lessen expression of TGFβ pathway components and thus protect cells from hyperactive signaling [51, 53], but DNA damage can also lead to gain of TGFβ signaling via stabilization of TGFβ receptor II [54]. Different from BRCA1 and FANCA, PALB2 is not considered to be a typical transcription factor [8, 48] and therefore constant DNA damage and its consequences may participate in enhancing TGFβ signaling in the *PALB2*-mutated cells. It is also worth noticing that reactive oxygen species (ROS) can release TGFβ1 from its latent form thus increasing the signaling [55]. PALB2 regulates ROS levels via KEAP1 interaction and inadequate PALB2 function can raise the cellular amount of ROS [9]. Therefore, there is also a potential direct link between *PALB2* mutations and TGFβ signaling.

TGFβ administration increased *KRT14* expression both in control and *PALB2*-mutated cells, *KRT14* thus being one of the numerous target genes of TGFβ. In agreement with the enhanced TGFβ signaling the gene was also endogenously highly upregulated in the *PALB2*-mutated cells. According to the transcriptome sequencing data, MCF10A cell lines expressed both the ligand gene *TGFβ1* as well as the receptor genes I-III and downstream *SMAD* genes (Supplementary Table 1d). The cell culture conditions, most notably GFR-BME could furthermore provide some extra TGFβ for the spheroids that would explain the higher *KRT14* expression in comparison to cells cultured as a monolayer. The control cells with lowish *KRT14* expression were more vulnerable to *KRT14* KD than the *PALB2*-mutated cells. The ability of *PALB2*-mutated cells and spheroids to agglomerate could also protect them against the *KRT14* KD, since cluster formation can considerably increase cancer cell survival [56, 57].

Knocking down *KRT14* expression reduced the protrusion formation in the spheroids, their assembly, as well as migration of the *PALB2*-mutated cells, which support the role of KRT14 in the observed migratory phenotype. KRT14 is an intermediate filament protein interacting with desmosome and hemidesmosome complexes, thus being able to affect intercellular and cell-basement membrane junctions [57]. It is abundantly appearing in the leading edge of invasive tumor cells and KRT14^+^ cells are suggested to have an essential role in the collective cancer cell dissemination [56, 57]. In mouse mammary cancer models KRT14 is needed for both invasion and distant metastasis [56, 57] and *KRT14* overexpression is also associated with an increased invasive potential of human epithelial carcinomas, such as breast and ovarian malignancies [57–59]. It is noteworthy that *BRCA1* and *FANCD2* mutation carriers commonly have KRT14-positive breast tumors [60, 61] and among sporadic breast cancer patients those with KRT14-positive tumors have markedly poorer prognosis than the patients with KRT14-negative tumors [61]. We show here that TGFβ can directly up-regulate *KRT14* expression in epithelial cells, and additionally TGFβ can affect the gene expression via stromal cells [59]. Correspondingly, KRT14-positive cells may mediate collective invasion both via cancer cell-intrinsic and stromal-dependent mechanisms [59]. Abundant DNA damage in *PALB2*-mutated KRT14-positive cells supports the hypothesis of mechanistic links between DNA damage, TGFβ signaling and *KRT14* expression though does not prove their causality. Recently it has been observed that *KRT14* expression can also be enhanced via selected hyper-methylation of H3K27 [62]. This may explain why the TGFβ inhibitor could not effectively reduce KRT14, and that *KRT14* KD limited the aggregation of the *PALB2*-mutated spheroids while LY2109761 did not.

At the early phase of malignancy TGFβ acts to suppress cell proliferation and stimulate apoptosis, but at the later stages it can promote tumorigenesis via induction of epithelial-mesenchymal transition (EMT) and resistance to anti-cancer drugs [63, 64]. Due to the variety of EMT programs, there are no exact morphological and molecular hallmarks to define EMT [65]. Nevertheless, *PALB2*-mutated cells did not show the typical EMT morphological features such as elongated cell shape. Expression of the master EMT regulator gene *TWIST2* had doubled in the #BiAll-93 transcriptome, but the expression of other selected key markers, such as *CDH1, CDH2, VIM, FN1, SNAI2,* and *ZEB1/2*, had not significantly changed in the *PALB2*-mutated cells, or their expression rather indicated transformation towards the epithelial cell type (Supplementary Table 1). Correspondingly, IPA of the whole transcriptome data did not indicate that EMT would take place in the *PALB2*-mutated cell lines. Thus, in the current context, despite enhanced TGFβ signaling *PALB2* mutations did not lead to the typical features of EMT.

PALB2-compromised cells exhibited several morphological changes, including invasive protrusions, and increased migratory capacity, pointing to a broader functional diversity of PALB2 than was previously anticipated, and beyond its well-known role in safeguarding genomic integrity. In the context of MCF10A cells, monoallelic *PALB2*-mutated cells replicated several phenotypes of the biallelic ones, albeit to a somewhat lesser extent, pointing to the importance of preserving normal wild type PALB2 dosage and functional abilities. Moreover, the severity of the phenotypes observed in PALB2-compromised cells indicated that further mutations, in addition to those already present in *PALB2* and the parental MCF10A cells, were not mandatory for the establishment of premalignant traits.

Altogether, the current findings from studies both from our group and by others accentuate the great importance of intact PALB2 function in the successful response to various exogenous and endogenous stresses, and show that one copy of *PALB2* is not enough to protect cells from these stresses. Further understanding of these mechanisms may eventually pave the way for improved personalized cancer therapy of *PALB2* mutation carriers, and possibly also to carriers of functionally related cancer susceptibility gene defects in *e.g*., BRCA1 and BRCA2.

### Supplementary Information

Below is the link to the electronic supplementary material.

Supplementary file1 (PDF 4629 KB)

Supplementary file2 (XLSX 252 KB)

Supplementary file3 (XLSX 53 KB)

Supplementary file4 (XLSX 606 KB)

Supplementary file5 (XLSX 79 KB)

Supplementary file6 (XLSX 27 KB)

Supplementary file7 (XLSX 15 KB)

Supplementary file8 (XLSX 64 KB)

## Data Availability

The data that support this study are available from the corresponding authors upon reasonable request. The transcriptome sequencing data generated for this publication have been deposited in NCBI Sequence Read Archive (SRA, RRID:SCR_004891) with BioProject (RRID:SCR_004801) ID PRJNA771273 (metadata available at https://dataview.ncbi.nlm.nih.gov/object/PRJNA771273?reviewer=m4h0jt1phelg8iaqdeka7geqsh).

## Abbreviations

3D: Three dimensional
B–H: Benjamini–Hochberg
DDR: DNA damage response
esiRNA: Endoribonuclease-prepared short interfering RNA
GFR-BME: Growth factor-reduced basement membrane extracts
HRD: Homologous recombination deficiency
HU: Hydroxy urea
IPA: Ingenuity pathway analysis
KD: Knock down
NFκB: Nuclear factor kappa B
ROS: Reactive oxygen species
STRING: Search tool for retrieval of interacting genes/proteins
TGFβ: Transforming growth factor beta
TGFβR: TGFβ receptor
TNFα: Tumor necrosis factor alpha

## References

[1] Slavin TP, Maxwell KN, Lilyquist J (2017). The contribution of pathogenic variants in breast cancer susceptibility genes to familial breast cancer risk. NPJ Breast Cancer.

[2] Lee JEA, Li N, Rowley SM (2018). Molecular analysis of PALB2-associated breast cancers. J Pathol.

[3] Yang X, Leslie G, Doroszuk A (2020). Cancer risks associated with germline PALB2 pathogenic variants: an international study of 524 families. J Clin Oncol.

[4] Reid S, Schindler D, Hanenberg H (2007). Biallelic mutations in PALB2 cause *Fanconi anemia* subtype FA-N and predispose to childhood cancer. Nat Genet.

[5] Xia B, Dorsman JC, Ameziane N (2007). *Fanconi anemia* is associated with a defect in the BRCA2 partner PALB2. Nat Genet.

[6] Zhang F, Ma J, Wu J (2009). PALB2 links BRCA1 and BRCA2 in the DNA-damage response. Curr Biol.

[7] Ducy M, Sesma-Sanz L, Guitton-Sert L (2019). The tumor suppressor PALB2: inside out. Trends Biochem Sci.

[8] Gardini A, Baillat D, Cesaroni M, Shiekhattar R (2014). Genome-wide analysis reveals a role for BRCA1 and PALB2 in transcriptional co-activation. EMBO J.

[9] Ma J, Cai H, Wu T (2012). PALB2 interacts with KEAP1 to promote NRF2 nuclear accumulation and function. Mol Cell Biol.

[10] Nikkilä J, Parplys AC, Pylkäs K (2013). Heterozygous mutations in PALB2 cause DNA replication and damage response defects. Nat Commun.

[11] Neve RM, Chin K, Fridlyand J (2006). A collection of breast cancer cell lines for the study of functionally distinct cancer subtypes. Cancer Cell.

[12] Soule HD, Maloney TM, Wolman SR (1990). Isolation and characterization of a spontaneously immortalized human breast epithelial cell line, MCF-10. Cancer Res.

[13] Ran FA, Hsu PD, Wright J (2013). Genome engineering using the CRISPR-Cas9 system. Nat Protoc.

[14] Tervasmäki A, Mantere T, Eshraghi L (2019). Tumor suppressor MCPH1 regulates gene expression profiles related to malignant conversion and chromosomal assembly. Int J Cancer.

[15] Debnath J, Muthuswamy SK, Brugge JS (2003). Morphogenesis and oncogenesis of MCF-10A mammary epithelial acini grown in three-dimensional basement membrane cultures. Methods.

[16] Wang L, Brugge JS, Janes KA (2011). Intersection of FOXO- and RUNX1-mediated gene expression programs in single breast epithelial cells during morphogenesis and tumor progression. Proc Natl Acad Sci U S A.

[17] Single A, Beetham H, Telford BJ (2015). A comparison of real-time and endpoint cell viability assays for improved synthetic lethal drug validation. J Biomol Screen.

[18] Morgan RG, Chambers AC, Legge DN (2018). Optimized delivery of siRNA into 3D tumor spheroid cultures in situ. Sci Rep 2018.

[19] Hall DMS, Brooks SA (2014). In vitro invasion assay using matrigel™: a reconstituted basement membrane preparation. Methods Mol Biol.

[20] Taglialatela A, Alvarez S, Leuzzi G (2017). Restoration of replication fork stability in BRCA1- and BRCA2-deficient cells by inactivation of SNF2-family fork remodelers. Mol Cell.

[21] Erkko H, Xia B, Nikkilä J (2007). A recurrent mutation in PALB2 in Finnish cancer families. Nature.

[22] Foulkes WD, Ghadirian P, Akbari MR (2007). Identification of a novel truncating PALB2 mutation and analysis of its contribution to early-onset breast cancer in French–Canadian women. Breast Cancer Res.

[23] Rantakari P, Nikkila J, Jokela H (2010). Inactivation of Palb2 gene leads to mesoderm differentiation defect and early embryonic lethality in mice. Hum Mol Genet.

[24] Ciccia A, Elledge SJ (2010). The DNA damage response: making it safe to play with knives. Mol Cell.

[25] Panier S, Boulton SJ (2014). Double-strand break repair: 53BP1 comes into focus. Nat Rev Mol Cell Biol.

[26] Williams AB, Schumacher B (2016). p53 in the DNA-damage-repair process. Cold Spring Harb Perspect Med.

[27] Meek DW (2009). Tumour suppression by p53: a role for the DNA damage response?. Nat Rev Cancer.

[28] Helbling-Leclerc A, Garcin C, Rosselli F (2021). Beyond DNA repair and chromosome instability-*Fanconi anaemia* as a cellular senescence-associated syndrome. Cell Death Differ.

[29] Liu Q, Lopez K, Murnane J (2019). Misrepair in context: TGFβ regulation of DNA repair. Front Oncol.

[30] Barcellos-Hoff MH (2022). The radiobiology of TGFβ. Semin Cancer Biol.

[31] Bouquet F, Pal A, Pilones KA (2011). TGFβ1 inhibition increases the radiosensitivity of breast cancer cells in vitro and promotes tumor control by radiation in vivo. Clin Cancer Res.

[32] Zhou J, Wang H, Fu F (2020). Spectrum of PALB2 germline mutations and characteristics of PALB2-related breast cancer: screening of 16,501 unselected patients with breast cancer and 5890 controls by next-generation sequencing. Cancer.

[33] Antoniou AC, Foulkes WD, Tischkowitz M, Group PI (2015). Breast cancer risk in women with PALB2 mutations in different populations. Lancet Oncol.

[34] Li A, Geyer FC, Blecua P (2019). Homologous recombination DNA repair defects in PALB2-associated breast cancers. NPJ Breast Cancer.

[35] Staaf J, Glodzik D, Bosch A (2019). Whole-genome sequencing of triple-negative breast cancers in a population-based clinical study. Nat Med.

[36] Wark L, Novak D, Sabbaghian N (2013). Heterozygous mutations in the PALB2 hereditary breast cancer predisposition gene impact on the three-dimensional nuclear organization of patient-derived cell lines. Genes Chromosomes Cancer.

[37] Tharmapalan P, Mahendralingam M, Berman HK, Khokha R (2019). Mammary stem cells and progenitors: targeting the roots of breast cancer for prevention. EMBO J.

[38] Pereira EJ, Burns JS, Lee CY (2020). Sporadic activation of an oxidative stress-dependent NRF2-p53 signaling network in breast epithelial spheroids and premalignancies. Sci Signal.

[39] Roy R, Chun J, Powell SN (2011). BRCA1 and BRCA2: different roles in a common pathway of genome protection. Nat Rev Cancer.

[40] Mahdi AH, Huo Y, Tan Y (2018). Evidence of intertissue differences in the DNA damage response and the pro-oncogenic role of NF-kappaB in mice with disengaged BRCA1-PALB2 interaction. Cancer Res.

[41] Karaayvaz-Yildirim M, Silberman RE, Langenbucher A (2020). Aneuploidy and a deregulated DNA damage response suggest haploinsufficiency in breast tissues of BRCA2 mutation carriers. Sci Adv.

[42] Obermeier K, Sachsenweger J, Friedl TW (2016). Heterozygous PALB2 c.1592delT mutation channels DNA double-strand break repair into error-prone pathways in breast cancer patients. Oncogene.

[43] Zeman MK, Cimprich KA (2014). Causes and consequences of replication stress. Nat Cell Biol.

[44] Wilhelm T, Olziersky AM, Harry D (2019). Mild replication stress causes chromosome mis-segregation via premature centriole disengagement. Nat Commun.

[45] Heijink AM, Talens F, Jae LT (2019). BRCA2 deficiency instigates cGAS-mediated inflammatory signaling and confers sensitivity to tumor necrosis factor-alpha-mediated cytotoxicity. Nat Commun.

[46] Buisson R, Niraj J, Pauty J (2014). Breast cancer proteins PALB2 and BRCA2 stimulate polymerase eta in recombination-associated DNA synthesis at blocked replication forks. Cell Rep.

[47] Murphy AK, Fitzgerald M, Ro T (2014). Phosphorylated RPA recruits PALB2 to stalled DNA replication forks to facilitate fork recovery. J Cell Biol.

[48] Bleuyard JY, Fournier M, Nakato R (2017). MRG15-mediated tethering of PALB2 to unperturbed chromatin protects active genes from genotoxic stress. Proc Natl Acad Sci U S A.

[49] Luo K (2017). Signaling cross talk between TGF-β/Smad and other signaling pathways. Cold Spring Harb Perspect Biol.

[50] Liu L, Zhou W, Cheng CT (2014). TGFβ induces “BRCAness” and sensitivity to PARP inhibition in breast cancer by regulating DNA-repair genes. Mol Cancer Res.

[51] Zhang H, Kozono DE, O’Connor KW (2016). TGF-β inhibition rescues hematopoietic stem cell defects and bone marrow failure in *Fanconi anemia*. Cell Stem Cell.

[52] Satterwhite DJ, Matsunami N, White RL (2000). TGF-β1 inhibits BRCA1 expression through a pathway that requires pRb. Biochem Biophys Res Commun.

[53] Bai F, Wang C, Liu X (2022). Loss of function of BRCA1 promotes EMT in mammary tumors through activation of TGFβR2 signaling pathway. Cell Death Dis.

[54] Li Y, Liu Y, Chiang YJ (2019). DNA damage activates TGF-β signaling via ATM-c-Cbl-mediated stabilization of the type II receptor TβRII. Cell Rep.

[55] Jobling MF, Mott JD, Finnegan MT (2006). Isoform-specific activation of latent transforming growth factor β (LTGF-β) by reactive oxygen species. Radiat Res.

[56] Cheung KJ, Ewald AJ (2016). A collective route to metastasis: seeding by tumor cell clusters. Science.

[57] Cheung KJ, Padmanaban V, Silvestri V (2016). Polyclonal breast cancer metastases arise from collective dissemination of keratin 14-expressing tumor cell clusters. Proc Natl Acad Sci U S A.

[58] Bilandzic M, Rainczuk A, Green E (2019). Keratin-14 (KRT14) positive leader cells mediate mesothelial clearance and invasion by ovarian cancer cells. Cancers (Basel).

[59] Hanley CJ, Henriet E, Sirka OK (2020). Tumor-resident stromal cells promote breast cancer invasion through regulation of the basal phenotype. Mol Cancer Res.

[60] Lakhani SR, Reis-Filho JS, Fulford L (2005). Prediction of BRCA1 status in patients with breast cancer using estrogen receptor and basal phenotype. Clin Cancer Res.

[61] De Silva RS, Platt-Higgins A, Winstanley JHR (2011). Statistical association of basal cell keratins with metastasis-inducing proteins in a prognostically unfavorable group of sporadic breast cancers. Am J Pathol.

[62] Verma A, Singh A, Singh MP (2022). EZH2-H3K27me3 mediated KRT14 upregulation promotes TNBC peritoneal metastasis. Nat Commun.

[63] Heldin CH, Moustakas A (2016). Signaling receptors for TGF-β family members. Cold Spring Harb Perspect Biol.

[64] Zhang M, Zhang YY, Chen Y (2021). TGF-β signaling and resistance to cancer therapy. Front Cell Dev Biol.

[65] Katsuno Y, Derynck R (2021). Epithelial plasticity, epithelial-mesenchymal transition, and the TGF-β family. Dev Cell.

