# Supplementary material for: PALB2-mutated human mammary cells display a broad spectrum of morphological and functional abnormalities induced by increased TGFβ signaling
Source: Cell Mol Life Sci. 2024 Apr 10;81(1):173. doi: 10.1007/s00018-024-05183-6 (PMC11006627; doi:10.1007/s00018-024-05183-6)
Supplement: Supplementary file 1 — Supplementary file1 (PDF 4629 KB) [file 18_2024_5183_MOESM1_ESM.pdf]

Cellular and Molecular Life Sciences

PALB2-mutated human mammary cells display a broad spectrum of morphological and functional abnormalities induced by increased TGFβ signaling

Hanna Tuppurainen, Niina Laurila, Marjut Nätynki, Leila Eshraghi, Anna Tervasmäki, Louisa Erichsen, Claus Storgaard Sørensen, Katri Pylkäs, Robert Winqvist & Hellevi Peltoketo

Corresponding authors:

Hellevi Peltoketo
Laboratory of Cancer Genetics and Tumor Biology, Translational Medicine Research Unit, Biocenter Oulu and Faculty of Medicine, MRC Oulu, University of Oulu
Aapistie 5A, 90220 Oulu, FINLAND
hellevi.peltoketo@oulu.fi

Robert Winqvist
Laboratory of Cancer Genetics and Tumor Biology, Translational Medicine Research Unit, Biocenter Oulu and Faculty of Medicine, MRC Oulu, University of Oulu
Aapistie 5A, 90220 Oulu, FINLAND
robert.winqvist@oulu.fi

This PDF file includes:

Supplementary Methods
Supplementary References
Supplementary Figures 1 to 13
Supplementary Table 3

Other supplementary materials for this manuscript include the following spreadsheet excel-files:

Supplementary Tables 1, 2 and 4 to 8

Supplementary Methods

Guide RNAs and experimental conditions used in CRISPR/Cas9 gene-editing

Guide RNAs used for gene-editing

gRNA	Target DNA sequence 5' - 3'	Target in *PALB2*	Oligo pairs used for subcloning into all-in-one plasmids	Sequence 5' - 3'
PALB2-Ex4-A	CTAGCCTGTCGATTGTTAACAGG	Exon 4	PALB2Ex4-AFrw	CACCGCTAGCCTGTCGATTGTTAAC
			PALB2Ex4-ARev	AAACGTTAACAATCGACAGGCTAGC
PALB2-Ex4-B	GGTTCACAATGATCTGATGCTGG	Exon 4	PALB2Ex4-BFrw	CACCGGTTCACAATGATCTGATGC
			PALB2Ex4-BRev	AAACGCATCAGATCATTGTGAACC
PALB2-Ex5-A	GTCGAATTGTTTAGTATCACTGG	Exon 5	PALB2-Ex5-AFrw	CACCGTCGAATTGTTTAGTATCAC
			PALB2-Ex5-ARev	AAACGTGATACTAAACAATTCGAC
PALB2-Ex5-C	CATACCACCCTGCAAGTGTCAGG	Exon 5	PALB2-Ex5-CFrw	CACCGCATACCACCCTGCAAGTGTC
			PALB2-Ex5-CRev	AAACGACACTTGCAGGGTGGTATGC

Experimental setup

sgRNA	Cas9 protein	Target in *PALB2*	Achieved clones characterized in more detail
PALB2Ex4-A & PALB2Ex4-B	spCas9N	Exon 4	#BiAll-93, #MonoAll-5, #Ctrl-45
PALB2-Ex5-A	spCas9	Exon 5	#MonoAll-2.13, #Ctrl-2.52
PALB2-Ex5-A & PALB2-Ex5-C	spCas9N	Exon 5	#MonoAll-1.38

Clonal expansion, mutation screening and *PALB2* transcript identification of CRISPR/Cas9-edited cell lines

24-48 h after transfection with all-in-one plasmids the cells were sorted according to GFP signal with BD FACSAriaIIIu (Becton Dickinson). One to five cell colonies were expanded in the presence of 20%, twice-filtered conditioned medium to support survival of few-cell colonies. When targeting the fifth exon of *PALB2*, half of the few-cell colonies were also expanded in the presence of 10 µM p160ROCK inhibitor Y-27632 (Cayman Chemical Company #129830-38-2) to further sustain their growth [1]. Of the analyzed cell clones, #Ctrl-2.52 and #MonoAll-2.13 had the ROCK inhibitor support until the 24-well plate stage, after which the expansion was continued with standard MCF10A growth media. Clonal expansions were followed by extraction of genomic DNA with QuickExtract™ solution (Epicentre #QE09050) and PCR amplification. Standard PCR was carried out using LA Taq™ polymerase (TaKaRa #RR02A) with GC buffer II or *Taq* DNA polymerase (Qiagen #201205). The colonies were screened using heteroduplex analysis with standard agarose gel electrophoresis or Agilent 2100 Bioanalyzer (Agilent Technologies) with DNA 1000 Kit (Agilent Technologies #5067-1504). Mixed clones with indication of indel were further sorted for single-cell colonies and expansion and screening were repeated. PCR products were purified with exonuclease I and FastAP™ Thermosensitive Alkaline Phosphatase (Thermo Fisher Scientific #EF0651) and the positive clones were finally confirmed and identified by Sanger sequencing (ABI 3500xL Genetic Analyzer). The PCR and sequencing primers are listed below. Numbering of the clones was based on their original location on the screening plates and their prefixes #Ctrl-, #BiAll-, and #MonoAll- referred to their genotypes with intact *PALB2* and with biallelic or monoallelic *PALB2* mutations, respectively. One to two chromosomal regions that were predicted to be the most likely unspecific targets for

the used guide RNAs were also sequenced using primers listed below and no such off-target indels were detected. To identify *PALB2* transcripts, complementary DNA (cDNA) from RNA (extracted with RNeasy Mini kit, Qiagen #74104) was generated using the iScript^{TM} cDNA synthesis kit (Bio-Rad #1708891), and cDNA was PCR-amplified followed by partial purification and sequencing of the amplicons. The used *PALB2* primer pairs reached from the first to seventh exon and are listed below.

Primer pairs used for primary PCR and sequencing to identify gene-edited clones

Primer pair	Sequence 5' - 3'	Target in *PALB2*	Size of PCR product
FRW1	GGCACATGCACAGGACAACC	Exon 4 - Intron 4	600 bp
REV1	AGGCTGAGGCAGGAGGAACA		
FRW2	CCGTGGAGGCTGTCATTCAG	Exon 4	345 bp
REV2	CAATCGACAGGCTAGAAGTTGG		
FRW3	GCTGCACACCCCAACTTGCT	Exon 5 - Intron 5	352 bp
REV3	TTAAACGTGGAAGGCCCAATGC		

Two most potential off-target areas (*) for the guide RNAs used and primers used to PCR-amplify and sequence the region

gRNA	Potential off-target sequences	Location (hg19)	Mismatches to the guide	Frw Primer sequence 5' - 3'	Rev Primer sequence 5' - 3'	PCR product size
PALB2-Ex4-A	GTAGACTGTGGA TTGTTAACAGG	chr15: +74885736	3MMs [1:5:10]	CACGAGTAGCAT TGGGAGCA	GAAGACCTGGGGA TTGTGGG	352 bp
	AGAGGCTGTAGA TTGTTAACAAG	chr2: +192685135	4MMs [1:2:5:10]	CAGCTTTTTCTTC ATGCGGGA	CAGACTGGAAAGG GTCACCT	215 bp
PALB2-Ex4-B	AGTTCTCTATGAT CTGATGCCAG	chr13: -65007673	3MMs [1:6:8]	TGCTCAGCAGGG TTCAAGAAA	CACCTTCAACCACC GCTATG	566 bp
	CGACCAGAATGA TCTGATGCCAG	chr15: +30053445	4MMs [1:3:4:7]	TTACCTGCCATTG CTTGGCT	TGTGGGAAAATAA GTAGCACCCT	186 bp
PALB2-Ex5-A	GTCATAGTGTTTA GTATCACCAG	chrX: -135548931	3MMs [4:5:7]	GTGTGCTGCCACC TCAGAAG	GCTTATGGGCCTCC CTCAAAGA	374 bp
	CACAAATGGTTT AGTATCACAGG	chrX: -92678580	4MMs [1:2:4:8]	TGTGGTATCATCT TTGGTGTGTCA	AGAGGGAAAGATG GTGGAGGG	392 bp
PALB2-Ex5-C	N/A					

* http://crispr.mit.edu/guides/ and https://www.deskgen.com

N/A; potential off-target sequences with less than 4 mismatches and 10 b-long intact 3' area, were not found

Primers used to PCR-amplify and sequence PALB2 transcripts

Name	Sequence 5' - 3'	Target in *PALB2*
PALB2-FRW2	GGAAGCCCCTCAGCTGTG	Exon 1
PALB2delT-LONG3-FRW	GAAAGTAGCCGTGGAGGCTG	Exon 4
PALB2delT-TRUNC1-REV	ATGGAGCCGTGAAAGCATCATC	Exon 5
PALB2 1592delT-REV	TGGAGCCGTGAAAGCATCAT	Exon 5
PALB2-CAN-FRW2	ACGGTTGCGCCTGATGATAATG	Exon 5
PALB2-CAN-cDNA-REV2	TGCCAAGCATCCAGAGCTTTC	Exon 7

Combinations of the primers

Forward	Reverse	Target in *PALB2* cDNA
PALB2-FRW2	PALB2delT-TRUNC1-REV	Exons 1-5
PALB2delT-LONG3-FRW	PALB2 1592delT-REV	Exons 4-5
PALB2delT-LONG3-FRW	PALB2delT-TRUNC1-REV	Exons 4-5
PALB2-CAN-FRW2	PALB2-CAN-cDNA-REV2	Exons 5-7

Transcriptome data analysis

To increase the stringency of the transcriptome data analysis, the lists of differentially expressed genes (DEGs) provided by DESeq2 (RRID:SCR_015687) analysis in Chipster [2] (RRID:SCR_010939) were further filtered before analyses with IPA [3] (RRID:SCR_008653) and STRING [4] (RRID:SCR_005223). Only the genes present on BaseSpace® (basespace.illumina.com, RRID:SCR_011881) and/or Bioconductor/R [5] (RRID:SCR_006442) Tophat2 (RRID:SCR_013035)/Bowtie2 (RRID:SCR_016368) -aligned [6, 7] DESeq2 lists, in addition to the Chipster list, were included in the final list of DEGs. FPKM (fragments per kilobase per million mapped reads) values for each triplicate were obtained by CuffDiff (RRID:SCR_001647) analysis using the CuffLinks algorithm (v2.1.0, RRID:SCR_014597) [8] in Chipster. If the mean FPKM of triplicates was less than 1 in both mutant and control sample the corresponding gene was removed from the final list of DEGs. The final list contained 3577 and 2268 DEGs for #BiAll-93 and #MonoAll-2.13, respectively.

Quantification of senescence cells

Proportion of senescent cells was quantified using Senescence β-Galactosidase Activity Assay Kit (Cell Signaling Technology #35302) using low-passage parental MCF10A cells as a negative/low-senescence control. Senescent cells were sorted in BD FACSAriaIIIu according to size, granularity, and β-galactosidase activity of the cells (*ie*., $FSC^{high}/SSC^{high}/C_{12}FDG^{high}$ cells) as described in [9]. Results were analysed with FlowJo program package (BD Biosciences, RRID:SCR:008520), and C_{12}FDG-treated cells were imaged using Zoe fluorescent cell imaginer (BioRad).

qRT-PCR

To confirm transcriptome sequencing results and to compare gene expression in all *PALB2*-compromised cell lines, independent spheroids were grown, and RNA was extracted and quality-checked as for transcriptome sequencing. RNA integrity numbers of the samples ranged from 9.3 to 9.8. Transcripts of 21 selected genes were quantified with qRT-PCR using customized RT^2 Profiler PCR arrays (Qiagen #330171). The selected genes consisted of up- and down-regulated genes, expression of which was altered both in #BiAll-93 and #MonoAll-2.13 cells or only in #BiAll-93 cells according to the transcriptome data. These genes also included scarcely and abundantly expressed genes and DEGs with moderate to very high expression difference between the *PALB2*-compromised and their control cell lines. To compare *KRT14* expression between DMSO- and LY2109761-treated, esiRNA-eGFP- and esiRNA-KRT14-treated, as well as 2D- and 3D-grown cells, RNA was extracted in the same manner as for customized arrays, and RT^2 qPCR Primer Assay for human KRT14 (Qiagen #330001: NM_000526) was used for qRT-PCR. To measure the efficiency of *PALB2* knock down RNA was isolated from the cells grown as a monolayer and RT^2 qPCR Primer Assay for human PALB2 (Qiagen #330001: NM_024675) was used.

For RT^2 Profiler PCR arrays, 0.5 - 0.8 µg RNA was converted to cDNA using RT^2 First Strand kit (Qiagen #330404) and diluted following the manufacturer's RT^2 Profiler instructions. For *KRT14* expression analysis, 0.5 µg RNA was used for cDNA synthesis using the same kit, and the synthesis reaction was diluted to 1:2.5. The RT^2 Profiler PCR arrays and RT^2 Primer Assays were analyzed using RT^2 SYBR® Green qPCR Mastermix (Qiagen #330504) in CFX96 instrument (Bio-Rad, RRID:SCR_018064). The fold changes based on arithmetic mean and *p*-values based on a Student's t-test of the replicate $2^{\wedge(- \text{Delta CT})}$ values for each gene were calculated with the Qiagen online RT^2 profiler PCR Data Analysis tool (https://dataanalysis.qiagen.com/pcr/arrayanalysis.php) using *GAPDH* and *B2M* as reference genes. qPCR cut off value for customized arrays was set to either 37 or 35, depending on using 0.5 µg or 0.8 µg of RNA. Cut off 35 was used for KRT14 Primer Assays.

Western blot analysis

Cells were lysed into NETN-300 buffer (20 mM Tris pH 7.5, 300 mM NaCl, 1 mM EDTA, and 0.5% NP-40) containing cOmplete™ Mini EDTA-free protease inhibitor tablets (Roche Diagnostics #11836170001) and Phosphatase Inhibitor Cocktail Set V (Merck #524629), and protein concentrations were then measured with the Pierce BCA Protein Assay Kit (Thermo Scientific™ #23227). The cell lysates were denatured in Laemmli buffer containing DTT and equal amounts of protein were separated by SDS-PAGE using 4-15% Mini-PROTEAN® TGX™ gels (Bio-Rad #4561083). The proteins were then transferred to PVDF membrane with the Trans-Blot Turbo transfer system (Bio-Rad #1704156). The membranes were blocked with Blotto B (1% BSA and 1% non-fat milk) and incubated with a primary antibody over night at +4 °C. The membranes were washed with TBS buffer, pH 7.5, containing 0.05% Tween® 20 (Sigma-Aldrich #93773) followed by secondary antibody incubation for 1 h at room temperature. The signal was detected using SuperSignal™ West Pico or Femto chemiluminescent substrate (Thermo Scientific™ #34580 and #34095) and imaged with the Fujifilm LAS-3000 or Azure 600 (Azure Biosystems) gel documentation imaging systems. Band intensities were quantified using Azure Spot (Microsoft, RRID:SCR_011880) software. Amount of protein in each band was quantified against total protein on the lane. Sharpness, brightness, and contrast of the images may have been modified up to ± 10% to improve visibility, but it has not affected quantification of samples. The used antibodies and stains are listed below.

Immunocytochemistry

Monolayer cells were grown overnight on poly-L-lysine-coated Nunc™ Lab-Tek™ 8-well chamber slides (Thermo Scientific™ #154534) and were then treated with 2 µM etoposide (Pfizer #391870) for 5 or 10 h when applicable, fixed with 4% PFA, permeabilized with 0.5% Triton X-100 and blocked with a buffer containing 1% BSA, 0.2% Tween® 20 and 0.3 M glycine in PBS. The samples were first incubated with primary antibodies overnight at +4 °C and then with fluorophore-conjugated secondary antibodies for 1 h at room temperature. Nuclei were stained with mounting medium containing DAPI.

Spheroids grown on top of GFR-BME (Corning® Matrigel® #354230) -coated, Nunc™ Lab-Tek™ 16-well chamber slides (Thermo Scientific™ #178599) for 8 days were fixed with 2% PFA. For immunostaining of nucleus- and cytosol-located proteins, the spheroids were permeabilized with 0.5% Triton X-100. The spheroids were blocked with primary blocking buffer containing 0.1 M glycine, 0.1% BSA and 10% goat serum and secondary blocking buffer also containing 10 µg/ml goat anti-mouse $F(ab')_2$ fragment [10]. The spheroids were then incubated with primary antibodies and with F-actin stain Alexa Fluor 488 Phalloidin at room temperature, followed by PBS-0.1% BSA washes and incubation with fluorophore-conjugated secondary antibodies at room temperature. Finally, the spheroids were further

washed and nuclei in non-permeabilized and permeabilized cells were stained with Hoechst® 33342 and mounting medium containing DAPI, respectively. Each staining was repeated at least three times and at least six images of each sample were randomly captured with the same settings for analyses. The used antibodies and stains are listed below.

Antibodies and stains used in 2D and 3D immunocytochemistry and Western blotting.

**Primary antibodies**	**Identifier/ Reference**	**Species**	**Dilution/ Concentration**
53BP1 antibody	Novus Cat# NB100-305, RRID:AB_10001695	Rabbit polyclonal IgG	1:200 – 1:350
Recombinant Anti-Cytokeratin 14 antibody, EPR17350	Abcam Cat# ab181595, RRID:AB_2811031	Rabbit monoclonal IgG	1:500 - 1:1000
Anti-phospho-histone H2A.X (Ser139) antibody, JBW301	Millipore Cat# 05-636, RRID:AB_309864	Mouse monoclonal IgG1	1:200 – 1:500
PALB2 antibody, M11	Professor Bing Xia, Rutgers Cancer Institute of New Jersey, NJ, USA [11]	Rabbit polyclonal	1:4000
PALB2 antibody, E9R2W	Cell Signaling Technology Cat# 30253, RRID:AB_2895010	Rabbit monoclonal IgG	1:3000
Phospho-SMAD2 antibody (Ser465/467), 138D4	Cell Signaling Technology, Cat# 3108, RRID:AB_490941	Rabbit monoclonal IgG	1:1000 – 1:2500
p21 Waf1/Cip1 antibody, 12D1	Cell Signaling Technology Cat# 2947, RRID:AB_823586	Rabbit monoclonal IgG	1:2500
p53 antibody, DO-1	Santa Cruz Biotechnology Cat# sc-126, RRID:AB_628082	Mouse monoclonal IgG2a	1:1000
RAD51 antibody, 14B4	GeneTex Cat# GTX70230, RRID:AB_372856	Mouse monoclonal IgG2b	1:200
SMAD2 antibody, L16D3	Cell Signaling Technology, Cat# 3103, RRID:AB_490816	Mouse monoclonal IgG1	1:1000
**Secondary antibodies**			
Donkey Anti-Mouse Alexa Fluor® 647 AffiniPure	Jackson ImmunoResearch Labs Cat# 715-605-150, RRID:AB_2340862	Donkey polyclonal IgG (H+L)	1:500
Goat anti-Rabbit Superclonal™ Alexa Fluor® 488	Thermo Fisher Scientific Cat# A27034, RRID:AB_2536097	Goat recombinant polyclonal IgG (H+L)	1:500
Goat anti-Mouse Superclonal™ Alexa Fluor® 555	Thermo Fisher Scientific Cat# A28180, RRID:AB_2536164	Goat recombinant polyclonal IgG (H+L)	1:500
Goat anti-Rabbit Superclonal™ Alexa Fluor® 555	Thermo Fisher Scientific Cat# A27039, RRID:AB_2536100	Goat recombinant polyclonal IgG (H+L)	1:500
Peroxidase AffiniPure Goat anti-Rabbit IgG	Jackson ImmunoResearch Labs Cat# 111-035-144, RRID:AB_2307391	Goat polyclonal IgG (H+L)	1:5000 – 1:25000
Peroxidase AffiniPure Goat anti-Mouse IgG	Jackson ImmunoResearch Labs Cat# 115-035-146, RRID:AB_2307392	Goat polyclonal IgG (H+L)	1:10000
**Other antibodies**			

AffiniPure F(ab')$_2$ Fragment Goat Anti-Mouse	Jackson ImmunoResearch Labs Cat# 115-006-003, RRID:AB_2338466	Goat polyclonal IgG (H+L)	1:100
**Stains**			
Fluoroshield mounting medium with DAPI	Abcam Cat# ab104139	-	-
Hoechst® 33342 solution	Thermo Fisher Scientific Cat# 10150888	-	1 µg/ml
Alexa Fluor® 488 Phalloidin (F-actin stain)	ThermoFisher Scientific Cat# A12379	-	1:100
No-Stain Protein labelling reagent	ThermoFisher Scientific Cat# A44449	-	1:20
SYTOX Green Nucleic Acid Stain	ThermoFisher Scientific Cat# S7020		100 nM

Supplementary References

1. Liu X, Ory V, Chapman S, et al (2012) ROCK inhibitor and feeder cells induce the conditional reprogramming of epithelial cells. Am J Pathol 180:599–607. https://doi.org/10.1016/J.AJPATH.2011.10.036

2. Kallio MA, Tuimala JT, Hupponen T, et al (2011) Chipster: user-friendly analysis software for microarray and other high-throughput data. BMC Genomics 12:507. https://doi.org/10.1186/1471-2164-12-507 [doi]

3. Krämer A, Green J, Pollard J, Tugendreich S (2014) Causal analysis approaches in Ingenuity Pathway Analysis. Bioinformatics 30:523–530. https://doi.org/10.1093/BIOINFORMATICS/BTT703

4. Szklarczyk D, Gable AL, Lyon D, et al (2019) STRING v11: protein-protein association networks with increased coverage, supporting functional discovery in genome-wide experimental datasets. Nucleic Acids Res 47:D607–D613. https://doi.org/10.1093/NAR/GKY1131

5. Love MI, Huber W, Anders S (2014) Moderated estimation of fold change and dispersion for RNA-seq data with DESeq2. Genome Biol 15:. https://doi.org/10.1186/S13059-014-0550-8

6. Kim D, Pertea G, Trapnell C, et al (2013) TopHat2: accurate alignment of transcriptomes in the presence of insertions, deletions and gene fusions. Genome Biol 14:. https://doi.org/10.1186/GB-2013-14-4-R36

7. Langmead B, Salzberg SL (2012) Fast gapped-read alignment with Bowtie 2. Nat Methods 9:357–359. https://doi.org/10.1038/NMETH.1923

8. Trapnell C, Roberts A, Goff L, et al (2012) Differential gene and transcript expression analysis of RNA-seq experiments with TopHat and Cufflinks. Nat Protoc 7:562–578. https://doi.org/10.1038/NPROT.2012.016

9. Goy E, Martin N, Drullion C, et al (2023) Flow Cytometry-based Method for Efficient Sorting of Senescent Cells. Bio Protoc 13:. https://doi.org/10.21769/BIOPROTOC.4612

10. Wang L, Brugge JS, Janes KA (2011) Intersection of FOXO- and RUNX1-mediated gene expression programs in single breast epithelial cells during morphogenesis and tumor progression. Proc Natl Acad Sci U S A 108:803. https://doi.org/10.1073/pnas.1103423108 [doi]

11. Ma J, Cai H, Wu T, et al (2012) PALB2 interacts with KEAP1 to promote NRF2 nuclear accumulation and function. Mol Cell Biol 32:1506–1517. https://doi.org/10.1128/MCB.06271-11 [doi]

Supplementary Fig. 1 MCF10A cell line authenticity report.

ECACC European Collection of Authenticated Cell Cultures Operated by Public Health England

Cell Line Authentication Report for Case Number 12140 – University of Oulu.

Statement

We have now completed DNA analysis of the sample presented for cell line authentication. Analysis has been conducted using the Promega Powerplex 16 HS kit which analyses the differences at 16 distinct hypervariable genetic loci. (Please note that some cell lines may exhibit genetic instability as they proliferate leading to discrepancies within the DNA profiles examined).

Summary

A short tandem repeat (STR) DNA Profile has been generated from the sample provided by Katri Pylkas of the University of Oulu. The sample name is GTL/MCF10A. The profile is shown in the table below together with the STR profile for cell line MCF-10A from the Cellosaurus website (ref. CVCL_0598). The profiles match 100%. This indicates these cell lines were generated from the same source material. Please refer to the detailed results on the second page for further information and interpretation and the notes below on interpreting cell line STR profiles.

Notes on interpretation of cell line STR profiles:

The outcome percentage is calculated using a formula which compares the number of alleles present against the number of alleles shared between the two DNA profiles. The outcome is designated one of the following statements based upon the outcome percentage (more data on the interpretation of cell line STR profiles can be found on the International Cell Line Authentication committee (ICLAC) website using the following link www.iclac.org/resources/match-criteria-worksheet

(1) For two cell lines with STR profiles matching greater than 80% they are considered to have been generated from the same source.
(2) For two cell lines with STR profiles matching between 56-79% they are unlikely to have been generated from the same source but further investigation should be carried out.
(3) For two cell lines with STR profiles matching less than 56% are considered to be unrelated, that is, to have been generated from independent sources.
(4) On occasion, the cell line STR profile may match one of the cell lines on the international list of misidentified cell lines curated by ICLAC. If this is the case this will be indicated in the summary statement. This list can be found at www.iclac.org/wp-content/uploads/Cross-Contaminations-v8_0.pdf

Summary statement prepared by:
Edward Burnett
Culture Collections
Scientific Development Group
Project Manager

E. Burnett

Date: 11th December 2018

Culture Collections, Public Health England, Porton Down, Salisbury, SP4 0JG, UK
T: +44 (0) 1980 612512 F: +44 (0) 1980 611315 E: culturecollections@phe.gov.uk W: www.phe-culturecollections.org.uk
@ECACC @NCTC_3000 @NCPV @NCPF

ECACC European Collection of Authenticated Cell Cultures Operated by Public Health England
NCTC National Collection of Type Cultures Operated by Public Health England
NCPV National Collection of Pathogenic Viruses Operated by Public Health England
NCPF National Collection of Pathogenic Fungi Operated by Public Health England

Laboratory Report

Test Requested	Cell Line Authentication
Case Number	12140
Date Sample Tested	1/12/2018
Date Sample Reported	11/12/2018

Sample Name	Sample/Comparison Profile Source	Sample Reference
GTL/MCF10A	Cellosaurus MCF-10A CVCL_0598	SC_01_D1022995_12140_LW326-18

Table of Allelic Data

STR Locus	Genotypes	
	Test Sample – GTL/MCF10A – University of Oulu	Database Sample – Cellosaurus MCF-10A CVCL_0598
AMEL	X, X	X, X
CSF1PO	10, 12	10, 12
D13S317	8, 9	8, 9
D16S539	11, 12	11, 12
D18S51	18, 19	18, 19
D21S11	29, 30	29, 30
D3S1358	14, 18	14, 18
D5S818	10, 13	10, 13
D7S820	10, 11	10, 11
D8S1179	14, 16	14, 16
FGA	22, 24	22, 24
PENTA D	10, 12	10, 12
PENTA E	13, 14	13, 14
THO1	8, 9.3	8, 9.3
TPOX	9, 11	9, 11
vWA	15, 17	15, 17

Culture Collections, Public Health England, Porton Down, Salisbury, SP4 0JG, UK
T: +44 (0) 1980 612512 F: +44 (0) 1980 611315 E: culturecollections@phe.gov.uk W: www.phe-culturecollections.org.uk
@ECACC @NCTC_3000 @NCPV @NCPF

Supplementary Fig. 2 *PALB2* protein products and transcripts of selected CRISPR/Cas9-edited MCF10A cell lines and their controls.

Validation of M11 and E9R2W PALB2 antibodies using *PALB2* knock down with esiRNA.

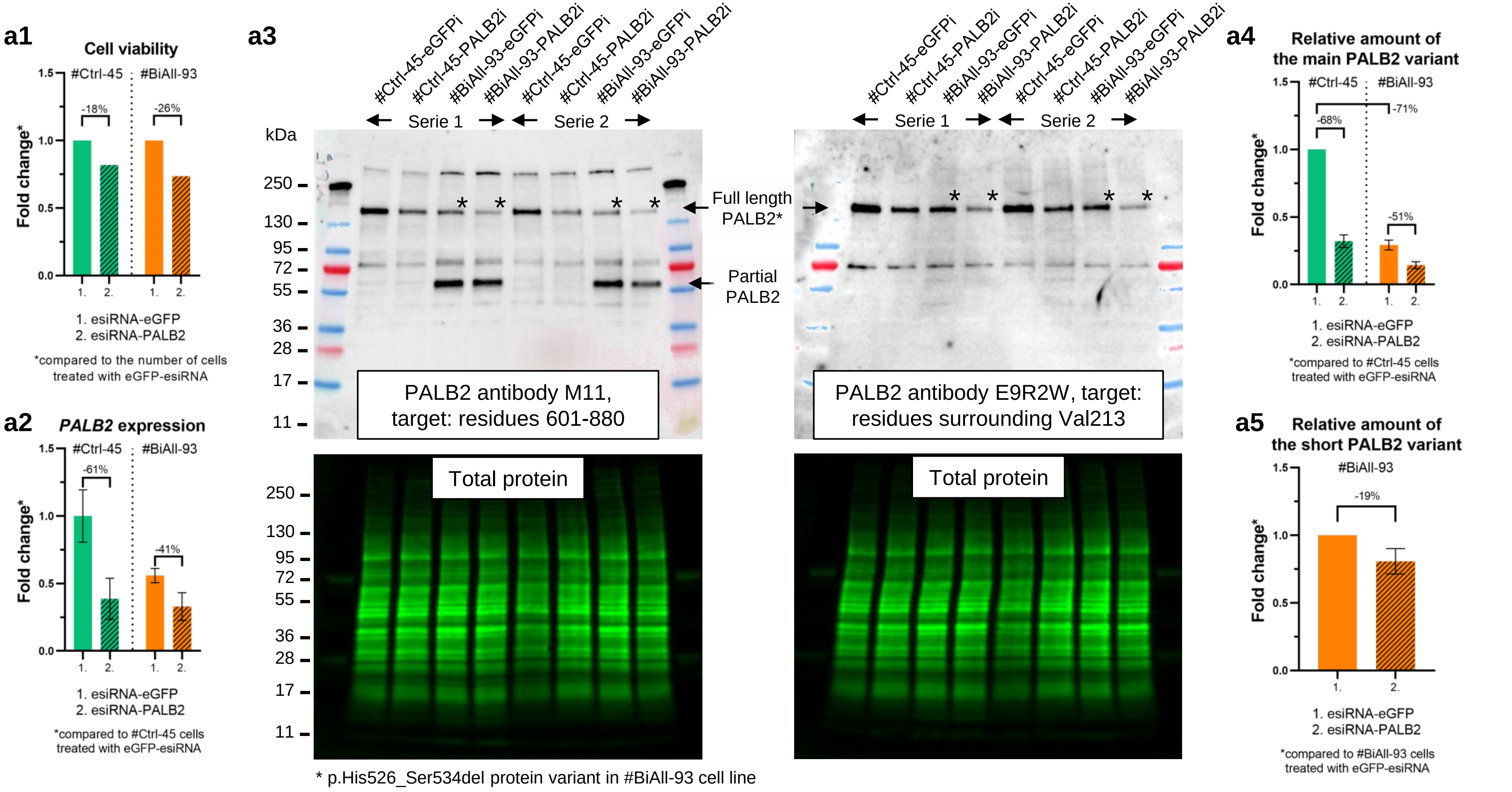

Supplementary Fig. 2 *PALB2* protein products and transcripts of selected CRISPR/Cas9-edited MCF10A cell lines and their controls.

b

Supplementary figures for Fig. 1**:** *PALB2* protein products recognized by M11 and E9R2W antibodies.

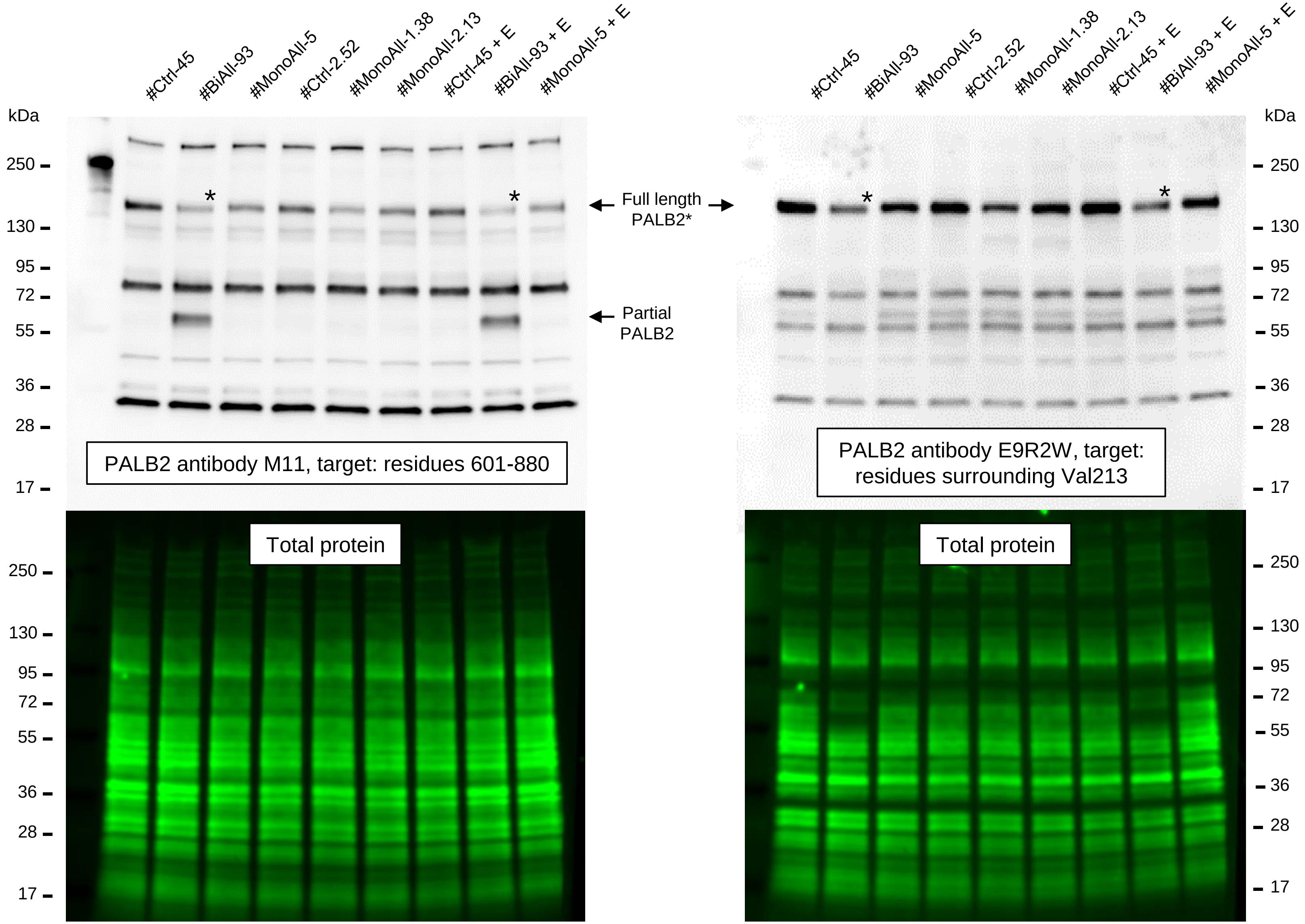

* p.His526_Ser534del protein variant in #BiAll-93 cell line

Supplementary Fig. 2 *PALB2* protein products and transcripts of selected CRISPR/Cas9-edited MCF10A cell lines and their controls.

Mutations at exon 4 and surrounding areas of *PALB2* in #BiAll-93 and #MonoAll-5 cDNAs.

c

#BiAll-93 cDNA: exon 3/4 boundary and corresponding areas in variant transcripts (vtrx) 1-3

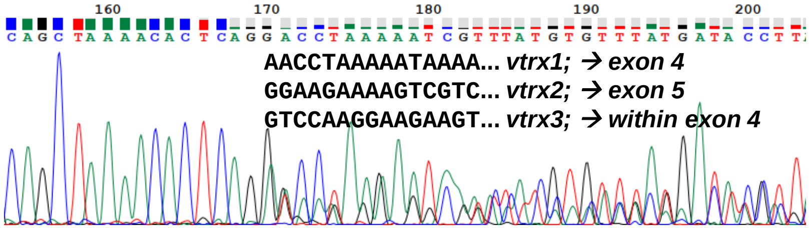

#BiAll-93 cDNA: variant transcripts 2-3

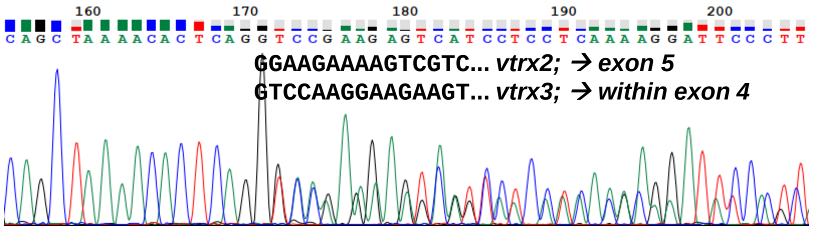

#BiAll-93 cDNA: variant transcript 3

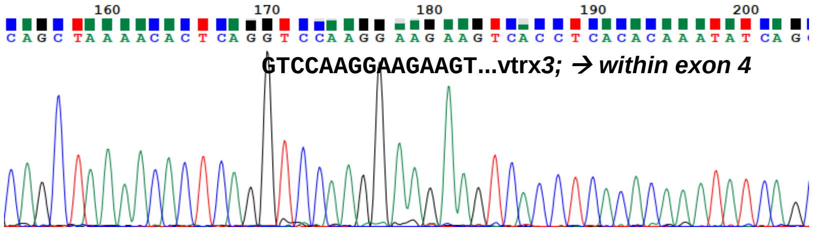

d

#Ctrl-45 cDNA: exon 4

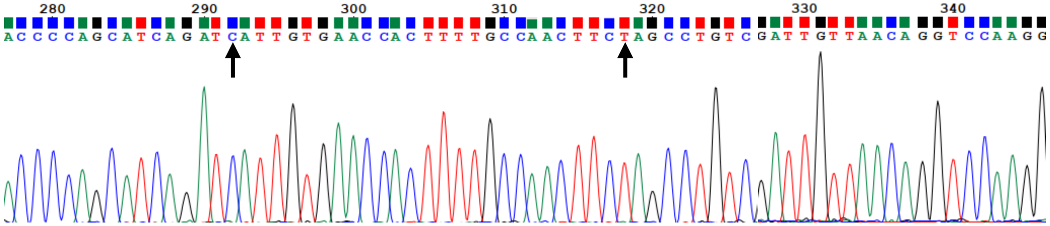

#BiAll-93 cDNA: variant transcript 1; c.1576_1602del

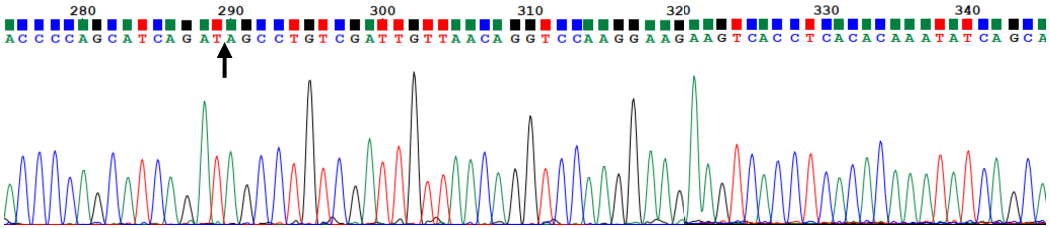

#MonoAll-5 cDNA: wild type transcript (trx) and variant transcript (vtrx) c.1579_1607dup

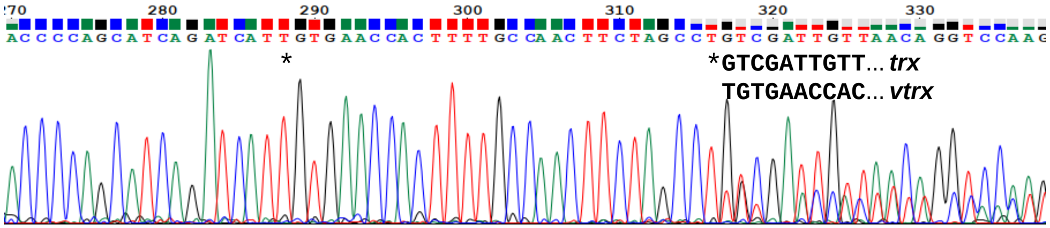

Supplementary Fig. 2 *PALB2* protein products and transcripts of selected CRISPR/Cas9-edited MCF10A cell lines and their controls.

Mutations at exon 5 of *PALB2* in #MonoAll-2.13 and #MonoAll-1.38 cDNAs.

e

#Ctrl-2-52 cDNA: exon 5

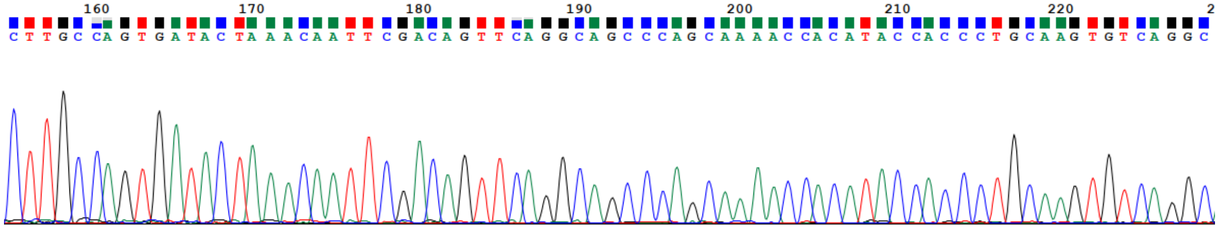

#MonoAll-2.13 cDNA: wild type transcript (trx) and variant transcript (vtrx) c.2315Adup

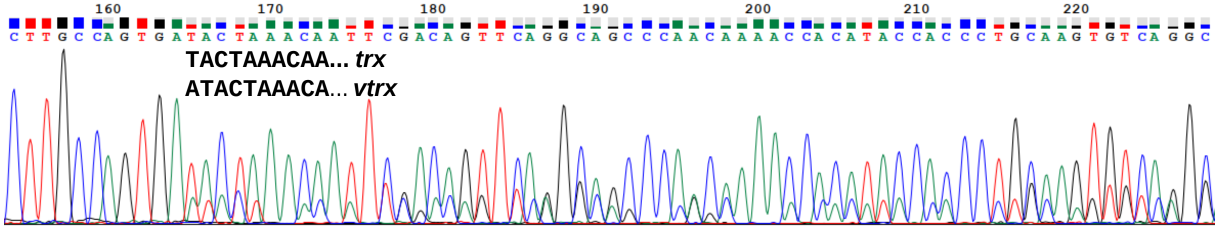

#MonoAll-1.38 cDNA wild type transcript (trx) and variant transcript (vtrx) c.2358_2383del

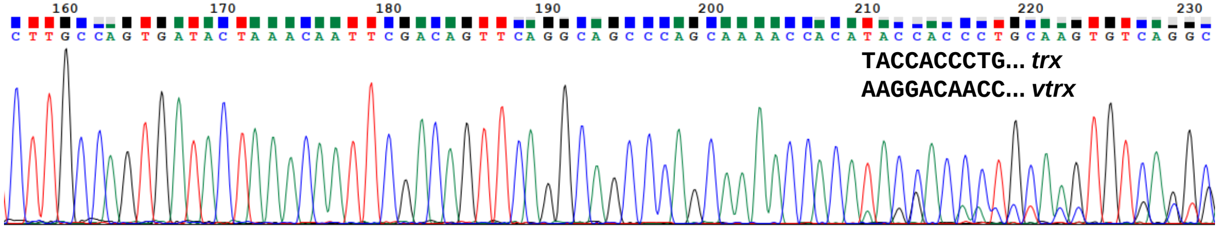

Supplementary Fig. 2 *PALB2* protein products and transcripts of selected CRISPR/Cas9-edited MCF10A cell lines and their controls. **a** Validation of PALB2 antibodies by knocking down *PALB2*. **a1** Viability of #Ctrl-45 and #BiAll-93 cells 48 hours after transfection with 10 nM control eGFP-esiRNA (plain bars) or PALB2-esiRNA (striped bars) shows that the cell lines have tolerated *PALB2* KD well in comparison to control esiRNA. **a2** 10 nM PALB2-esiRNA treatment has reduced *PALB2* expression approximately 60 and 40 per cent in #Ctrl-45 and #BiAll-93 cells, respectively. KD efficiency has been measured by qRT-PCR and 2^(-delta CT) values have been calculated using *GAPDH* and *B2M* as reference genes. **a3** Western blot analyses of #Ctrl-45 and #BiAll-93 cells after treatments with 10 nM control eGFP-esiRNA (eGFPi) or PALB2-esiRNA (PALB2i) using M11 and E9R2W PALB2 antibodies that have been targeted to PALB2 residues 601-880 or surrounding Val213, respectively. Both antibodies have recognized protein bands of same sizes that have visibly faded after knocking down *PALB2* expression and represent full length PALB2 protein in #Ctrl-45 cells and p.His526_Ser534del protein variant (asteriks) in #BiAll-93 cell line. M11 antibody has also recognized a shorter PALB2 protein product, amount of which has also been reduced by PALB2-esiRNA treatment. Each esiRNA-treatment has been carried out twice (series 1 and 2) and results from immunostaining with M11 and E9R2W antibodies have been combined in **a4** and **a5** to quantify PALB2 protein products. Intensity of PALB2 variant and total protein signals have been defined using Azure 600 imager and final results have been calculated with Azure Spot program. **a1, a2, a4, a5** Each bar represents the mean and error bars show the SD of quadruplicates (a4) or duplicates (a2, a5). SD, standard deviation **b** *PALB2* protein products in bi- and monoallelic *PALB2*-mutated cell lines and their control lines. PALB2 has been reduced in the mutated cell lines, and 0.05 µM etoposide (E) treatment for three days does not visibly affect expression of it. The membrane shown on the left has first been immunostained with M11 antibody, then stripped and re-stained with E9R2W antibody. **a, b** Near-complete p.His526_Ser534del protein variant in #BiAll-93 cell line has been marked with an asterisk. **c** Boundary region between exons 3 and 4 and corresponding areas in the #BiAll-93 transcripts. cDNA-derived, partially purified PCR fragments containing a combination of transcripts 1–3 (upmost row), 2–3 (middle row), and 3 alone (lowest row). Transcript 1 has an intact junction from exon 3 to 4, transcript 2 skips exon 4 in-frame joining exon 3 to exon 5, while in transcript 3 exon 3 joins out-of-frame inside exon 4.**d** *PALB2* mutations in exon 4 of *PALB2* transcripts in #BiAll-93 and #MonoAll-5 cells. Nucleotides from C to T indicated with arrows in #Ctrl-45 *PALB2* cDNA (upmost row) are missing from #BiAll-93 transcript 1 (middle row). Nucleotides from T to T indicated with asterisks have been duplicated in the second transcript of #MonoAll-5 *PALB2* (lowest row). **e** Mutations in exon 5 of *PALB2* transcripts. Upmost row demonstrates sequence in the control cell line #Ctrl-2.52 and the middle one shows the duplication of one nucleotide in *PALB2* transcript 2 of #MonoAll-2.13. 26 nucleotides are missing from *PALB2* transcript 2 of #MonoAll-1.38 (lowest row)

Supplementary Fig. 3

Monolayer and spheroid morphology of, and senescence in *PALB2*-mutated and control cells.

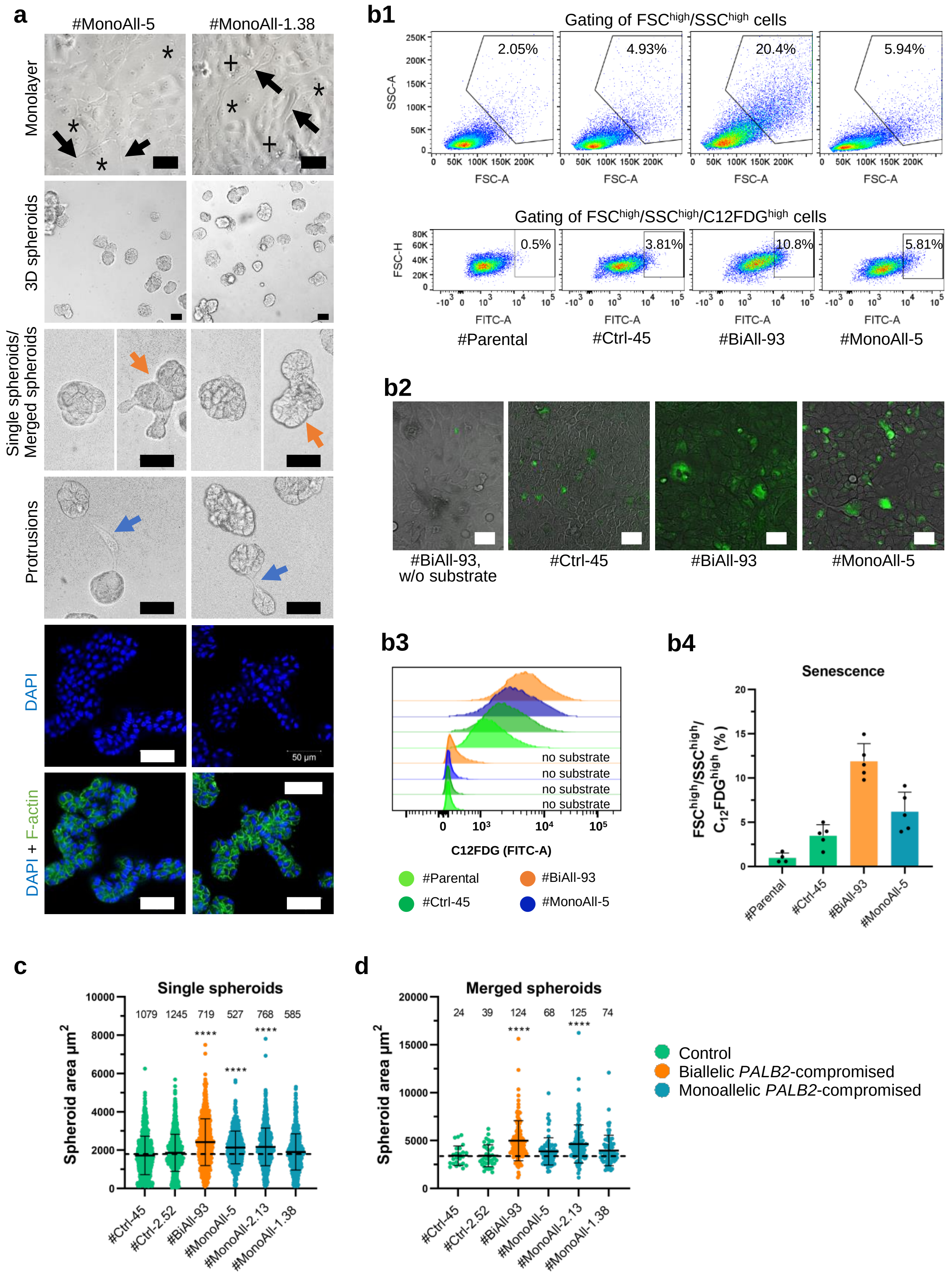

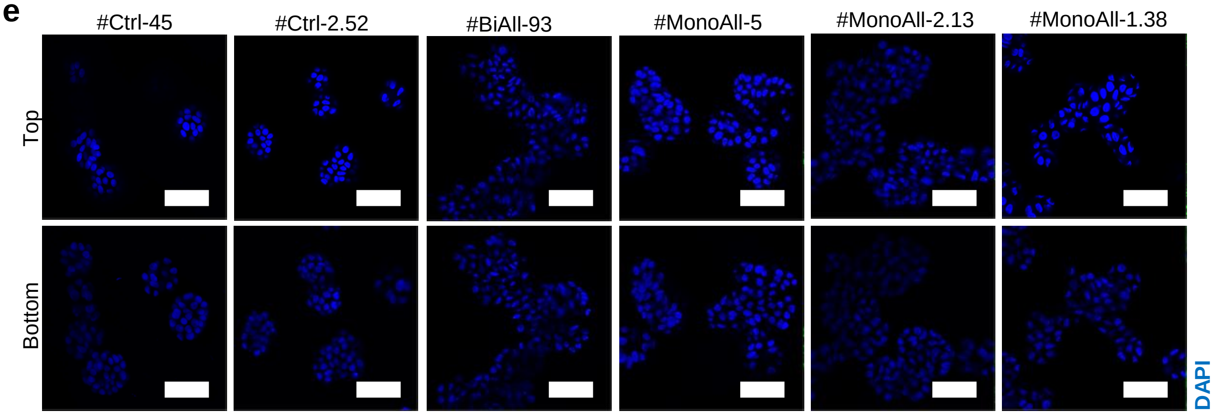

Supplementary Fig. 3 Monolayer and spheroid morphology of #MonoAll-5 and #MonoAll-1.38 follow those of other monoallelic *PALB2*-mutated cell lines, and senescence has increased in *PALB2*-mutated cells. **a** Representative light and fluorescence microscopy images. In monolayer culture (1st row), cells with vacuolized cytosol (plus symbols), cytosolic protrusions (black arrows) and senescence-like large and flat appearance (asterisks) are plentiful. Spheroids derived from #MonoAll-5 and #MonoAll-1.38 cell lines (2nd to 6th row) are disarranged, easily merged (orange arrows), have several cell layers (blue DAPI staining), and have produced invasive protrusions towards each other and the surrounding matrix (blue arrows). Fluorescence images of equatorial cross sections of spheroids stained with DAPI (blue) and Alexa Fluor 488 Phalloidin (green) show nuclei and cell boundaries, respectively. **b** Senescence in *PALB2*-mutated cells. Cells have first been gated according to their size and granularity (FSC^{high}/SSC^{high}) (**b1**, upper panel) and furthermore according to C_{12}FDG fluorescence ($FSC^{high}/SSC^{high}/ C_{12}FDG^{high}$) (**b1**, lower panel). Representative images with numbers stating the proportion of (FSC^{high}/SSC^{high}) and ($FSC^{high}/SSC^{high}/ C_{12}FDG^{high}$) cells in each cell line, respectively, are shown. Low passage MCF10A cells have been used as a low-senescence control. In **b2** representative C_{12}FDG-stained images of #Ctrl-45, #MonoAll-5 and #BiAll-93 cells are shown, and #BiAll-93 cells without β-galactosidase substrate serve as a negative staining control. Half offset diagram with biex scale **(b3)** shows how fluorescence of *PALB2*-mutated cells is distinctively shifted to the right. **b4** Proportion of ($FSC^{high}/SSC^{high}/ C_{12}FDG^{high}$) cells as quantified from five replicates. **a, b2** Sharpness, brightness and contrast of the images have been modified to improve visibility. **c** and **d** Scatter dot plot presentation of quantification of cross-sectional areas (μm^2) of single (**c**) and merged (**d**) spheroids separately in control cell lines, #BiAll-93 and three monoallelic PALB2-deficient cell lines. Horizontal lines designate mean values (±SD) of three independent replicates, and dashed line represents the mean of the two control cell lines. Statistical significance was determined by Kruskal-Wallis test with Dunn's multiple comparison post-test. **e** DAPI-stained images of top and bottom cross sections of control and PALB2-compromised spheroids, corresponding to the equatorial cross section images in Fig. 2a and Supplementary Fig. 3a. **a, b2, e** Scale bars, 50 µm. **c-d** The cell lines were grown as triplicates and 6 to 15 images of different parts of each plate were randomly captured. The number of analyzed images is the same as in Fig. 2. The total number of analyzed spheroids is given on top of each plot (**c, d**). SD, standard deviation; C_{12}FDG, 5-dodecanoylaminofluorescein Di-β-D-galactopyranoside; ****$p < 0.0001$

Supplementary Fig. 4

Principal component analyses of transcriptome data from #BiAll-93 and #MonoAll-2.13 and their control cell lines.

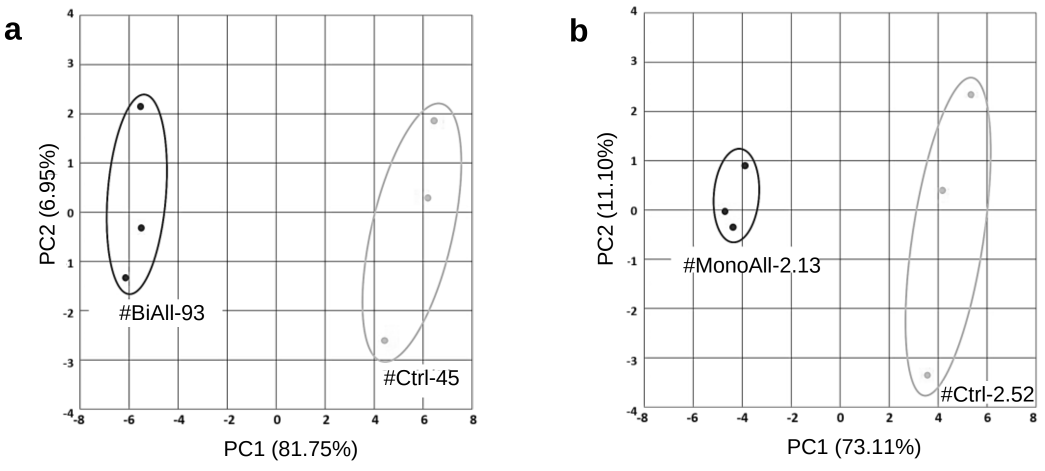

Supplementary Fig. 4 Principal component analyses (PCA) of transcriptome data processed with DeSeq2 differential gene expression analysis. **a** Clustering of #BiAll-93 triplicates separately from #Ctrl-45 ones. **b** Clustering of #MonoAll-2.13 triplicates separately from #Ctrl-2.52 ones

Supplementary Fig. 5

Increased DNA damage and decreased DNA repair in *PALB2*-mutated cells.

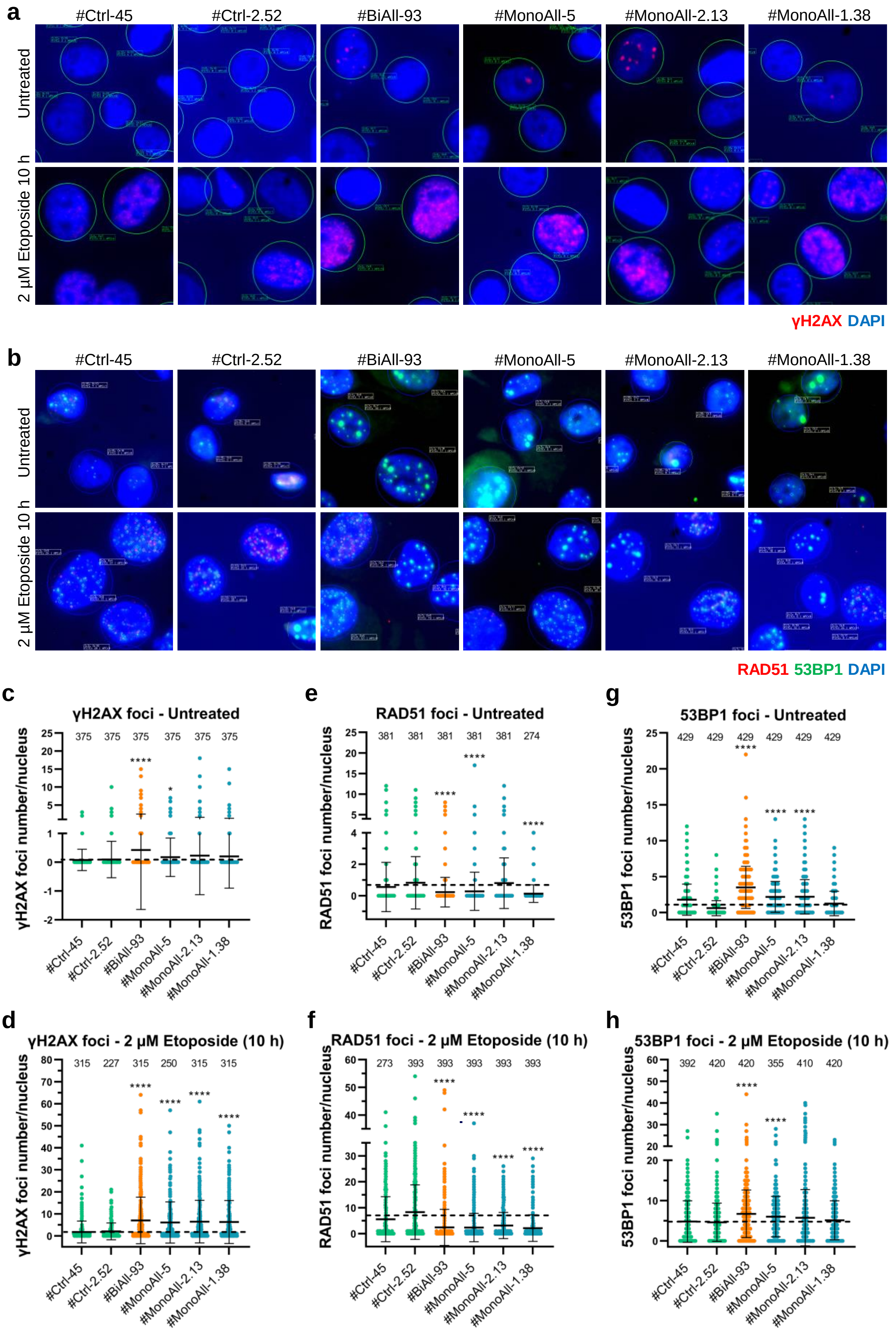

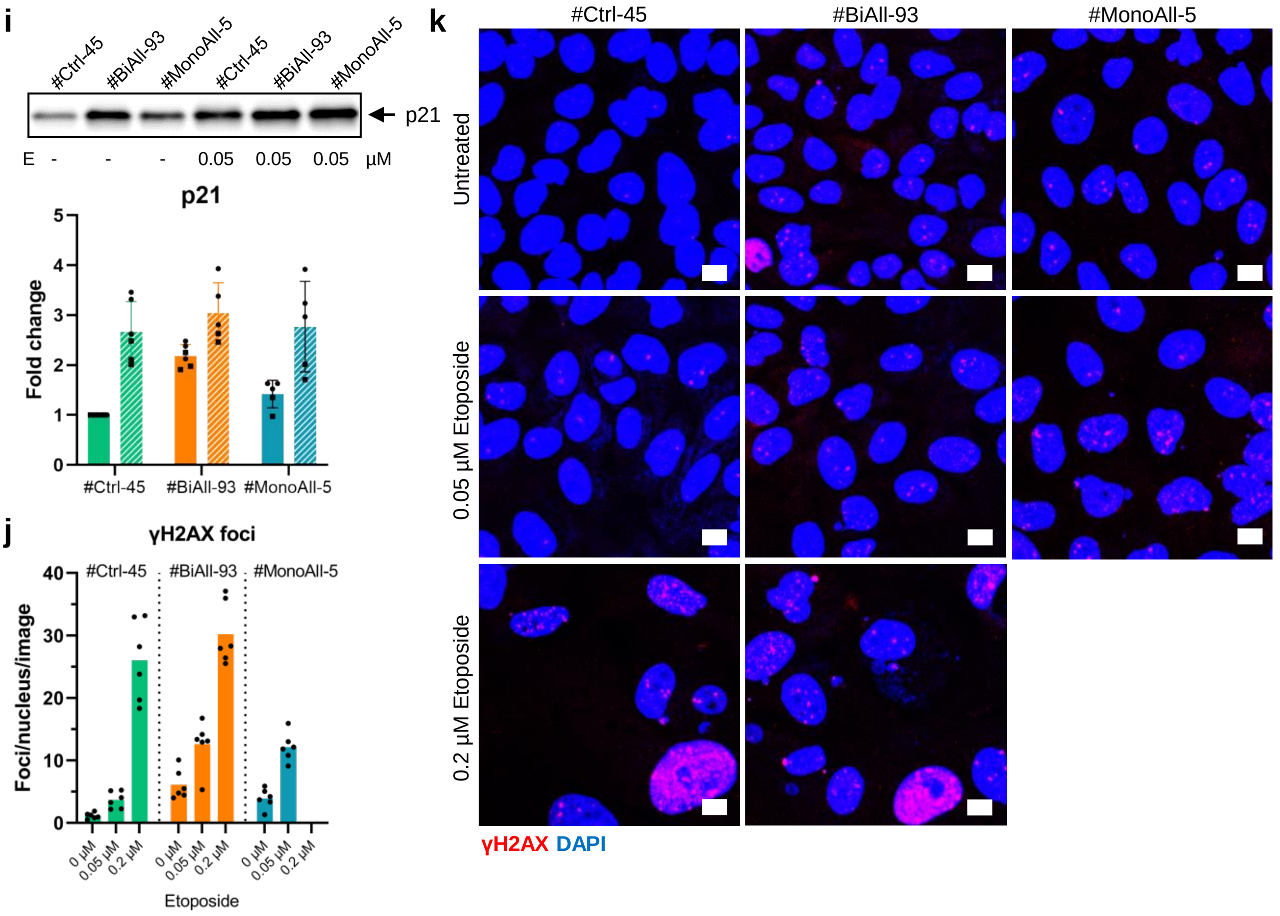

Supplementary Fig. 5 Increased DNA damage and decreased DNA repair in *PALB2*-mutated cells. **a**–**b** Representative images of AKLIDES® automated DNA repair foci analysis platform, showing *γ*H2AX (**a**) and RAD51 combined with 53BP1 (**b**) immunofluorescence staining with and without 10 h 2 µM etoposide treatment. Analyzed nuclei have been circulated and the number of foci in each nucleus has been calculated by the AKLIDES software. All cell lines were analyzed with the same settings. The original images have been cropped to show details of them as examples. The brightness of the images has been increased by 20%. **c**–**h** Number of γH2AX, RAD51 and 53BP1 foci/nucleus in control and *PALB2*-mutated cell lines without (**c, e, g**) and with (**d, f, h**) 10 h 2 µM etoposide treatment. Dots in the scatter dot plots represent the nuclei and the total number of nuclei counted from three or four replicates is shown on top of each plot. The horizontal lines designate the mean values (±SD). Dashed lines represent the mean of #Ctrl-45 and #Ctrl-2.52. Statistical significance was determined by Kruskal-Wallis test with Dunn's multiple comparison post-test. SD, standard deviation; **p* < 0.05, *****p* < 0.0001. **i** A representative image (upper panel) of p21 in #Ctrl-45, #BiAll-93 and #MonoAll-5 cells without (plain bars) and with (hatched bars) 0.05 µM etoposide (E) treatment for three days quantified from Western blot membranes (n = 5-6), and quantification of the membranes (lower panel). The bars depict the mean values (±SD) and the circle and square symbols biological replicates (n = 2). Total protein image for the upper panel is shown in Supplementary Fig 6a. **j** Mean number of *γ*H2AX foci per nucleus per image after 3 days without treatment and treatment with 0.05 or 0.2 µM etoposide. Six images, each including four z-stack slices, were randomly captured of each well, and the number of nuclei and foci were calculated using imageJ. Each dot represents the mean foci number per nucleus in one image, and the bars show the mean of six images in each sample. **k** Representative images of untreated cells and cells treated with 0.05 or 0.2 µM etoposide for 3 days. Each image is a maximum intensity projection combined from four z-stack slices captured across the sample. Brightness and contrast of the images have been enhanced by 80% and 50%, respectively. Scale bars, 10 µm

Supplementary Fig. 6

Complete Western blot membranes or their fragments combined with total protein staining.

Western blot supplementary figures for Fig. 4f and the related Supplementary Fig 5i

a

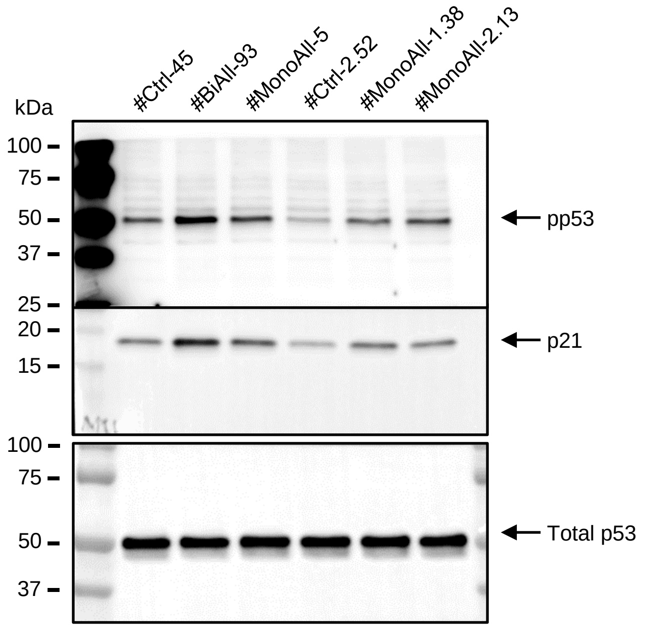

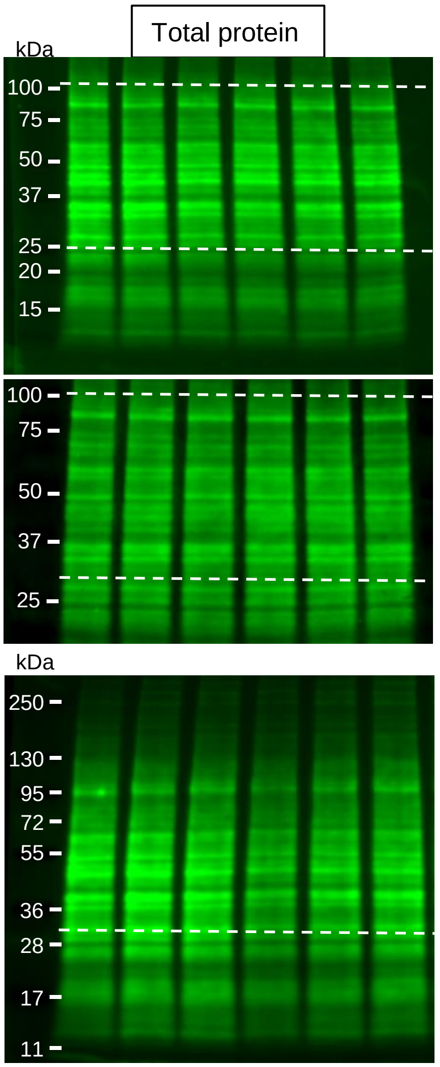

Supplementary figures for Fig. 4f

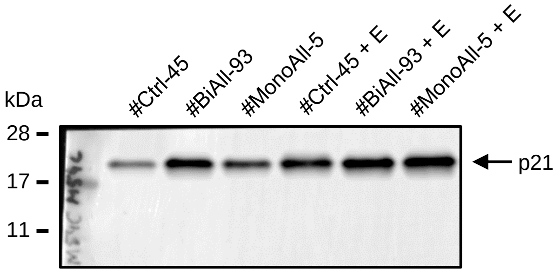

Supplementary figures for Supplementary Fig. 5i, upper panel

Western blot supplementary figures for Fig. 6

b

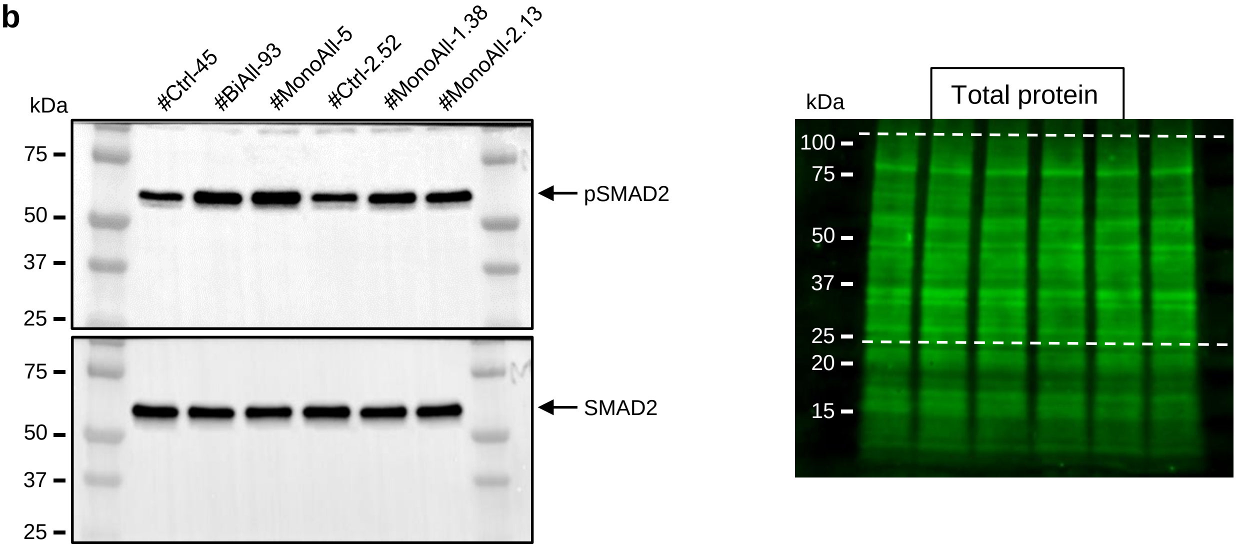

Supplementary figures for Fig. 6d

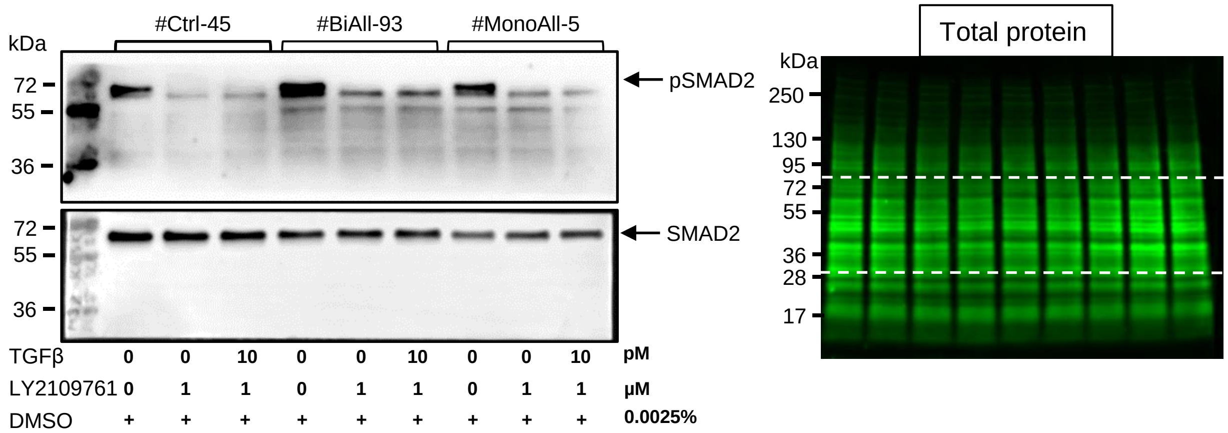

Supplementary figures for Fig. 6f

Western blot supplementary figures for Fig. 7

c

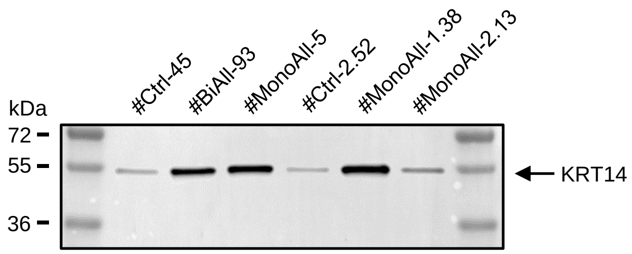

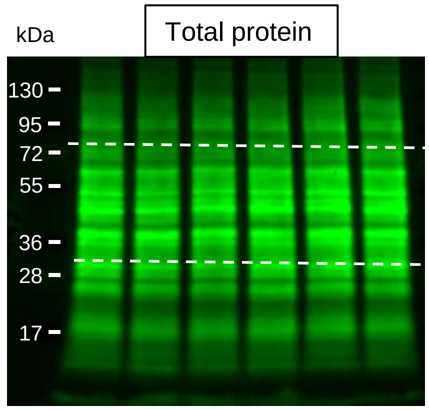

Supplementary figures for Fig. 7a ↑

Supplementary figures for Fig. 7d ↓

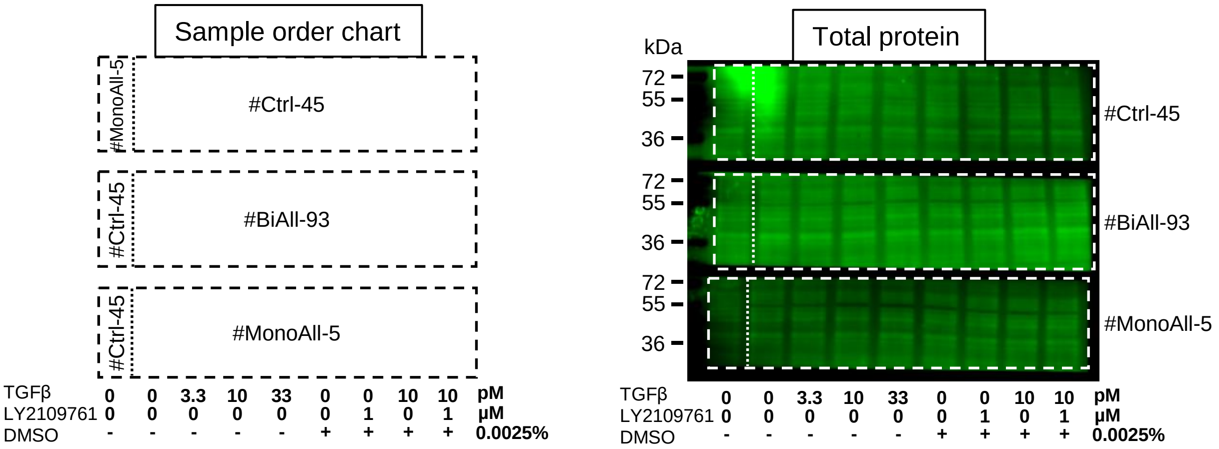

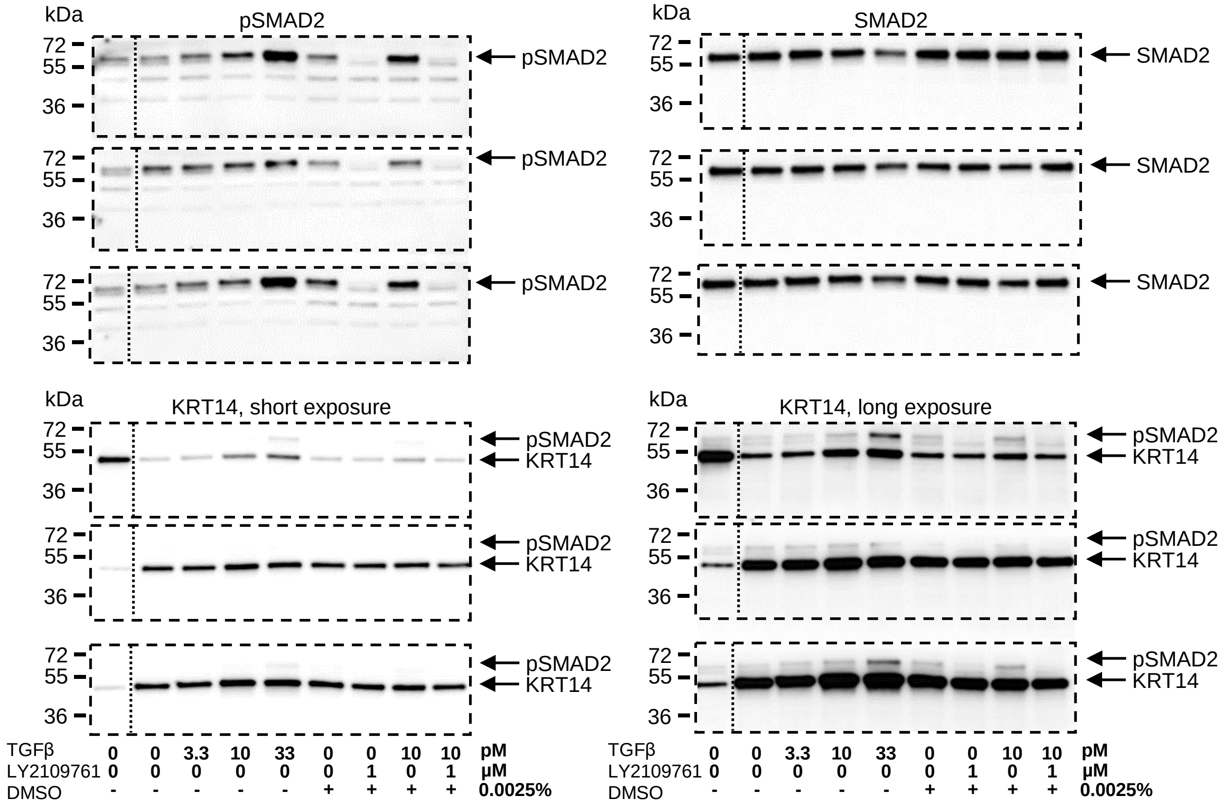

Supplementary Fig. 6 Complete Western blot membranes or their fragments combined with total protein staining. **a** Supplementary figures for Fig. 4 and Supplementary Fig. 5. 4f, upper panel: membranes showing pSer15-p53 (pp53), p21 and total p53 in *PALB2*-mutated and control cell lines and their corresponding total protein staining. Supporting Supplementary Fig 5i, upper panel: response of p21 to 0.05 µM etoposide (E) treatment for three days and corresponding total protein staining. **b** Supplementary figures for Fig. 6. 6d: membranes displaying pSer465/467-SMAD2 (pSMAD2) and SMAD2 in *PALB2*-mutated and control cell lines and corresponding total protein staining. 6f: response of pSMAD2 and SMAD2 to TGFβ receptor inhibitor LY2109761 in #BiAll-93, #MonoAll-5 and #Ctrl-45 cells, and corresponding total protein staining. **c** Supplementary figures for Fig. 7. 7a: a membrane presenting KRT14 in *PALB2*-mutated and control cell lines and corresponding total protein staining. 7d: response of pSMAD2, SMAD2 and KRT14 to TGFβ gradient and its receptor inhibitor LY2109761 in #BiAll-93, #MonoAll-5 and #Ctrl-45 cells. The three membranes as identified in the sample order chart have been treated and imaged together. The first lane of the membranes, separated with a dotted line had a sample different from the other lanes as shown in the sample order chart. **a-c** In most cases membranes have been cut to several pieces to enable various immunostainings simultaneously. Total protein staining has been imaged before cutting a membrane and cutting sites have been marked with a dashed line in total protein images. When available a membrane including molecular weight marker staining is shown

Supplementary Fig. 7
Chromosomal abnormalities in *PALB2*-mutated cells.

a

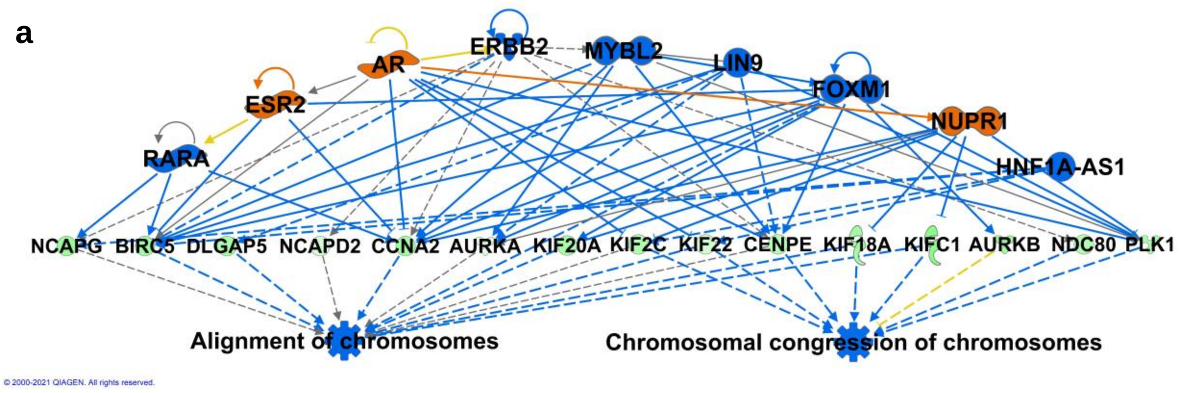

b

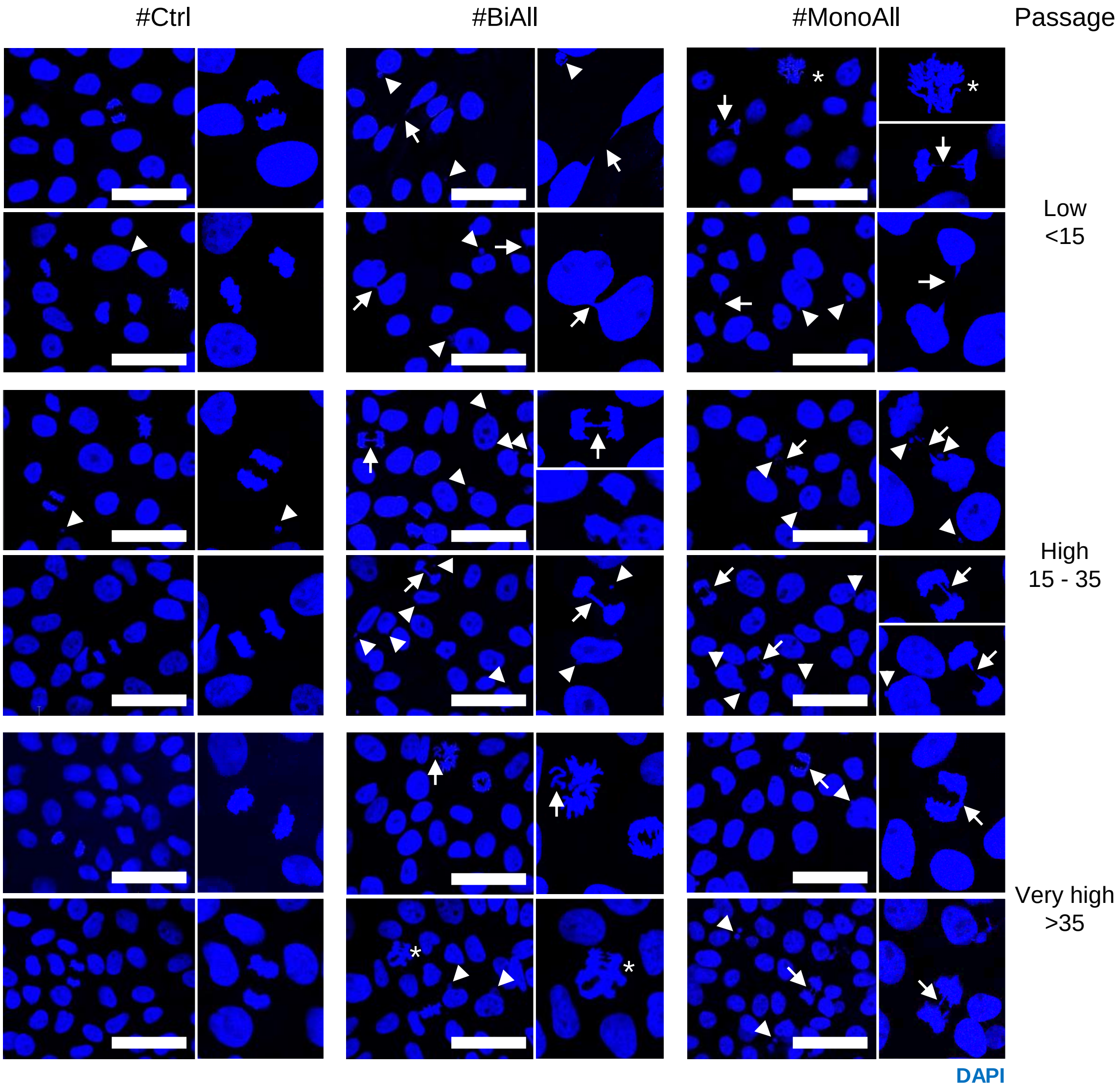

c

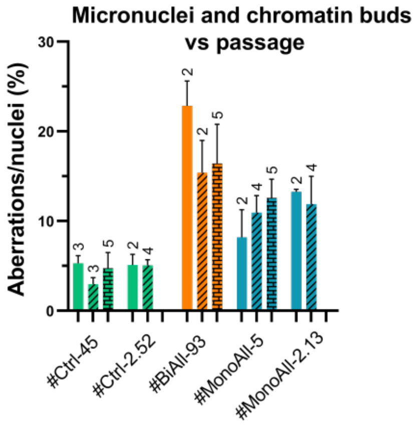

Supplementary Fig. 7 Chromosomal abnormalities in *PALB2*-mutated cells. **a** IPA regulator effect network with the second highest consistency score (8.004) for the biallelic *PALB2* mutant cell line #BiAll-93, showing predicted upstream regulators and phenotypic and functional outcomes related to a set of differentially expressed genes. Orange and blue symbols depict activation and inactivation, respectively, green symbols decreased transcription, orange and blue lines signaling leading to activation and inhibition, respectively, and yellow and grey lines indicate that the findings are inconsistent with state of downstream molecule or that the effect is not predicted. **b** Two representative images from each sample group (control cells and biallelically and monoallelically *PALB2*-mutated cells) demonstrating chromosomal aberrations at different passage phases are shown. Two-fold magnification of mitoses are shown on the right of each image. Nuclear DAPI-staining has been brightened and sharpened to enhance the items. Arrowheads, micronuclei or budding chromatins; arrows, chromatin bridges/lagging and loose chromosomes; asterisks, multipolar mitotic divisions; scale bars, 50 µm. **c** Ratio of number of micronuclei and chromatin buds to total nucleus count in control and PALB2-compromised cell lines from low (<15), high (15-35), and very high (>35) passage (mean + SD). Passage count represents number of cell splits after generation of a CRISPR/Cas9-modified MCF10A cell line. Number of replicates is given on top of each bar, and the total number of counted nuclei in each cell line is the same as in Fig. 5a. Plain, hatched and tiled bars represent samples from low, high and very high passage cells, respectively. SD, standard deviation

Supplementary Fig. 8
Migration and invasion in control and *PALB2*-mutated cells.

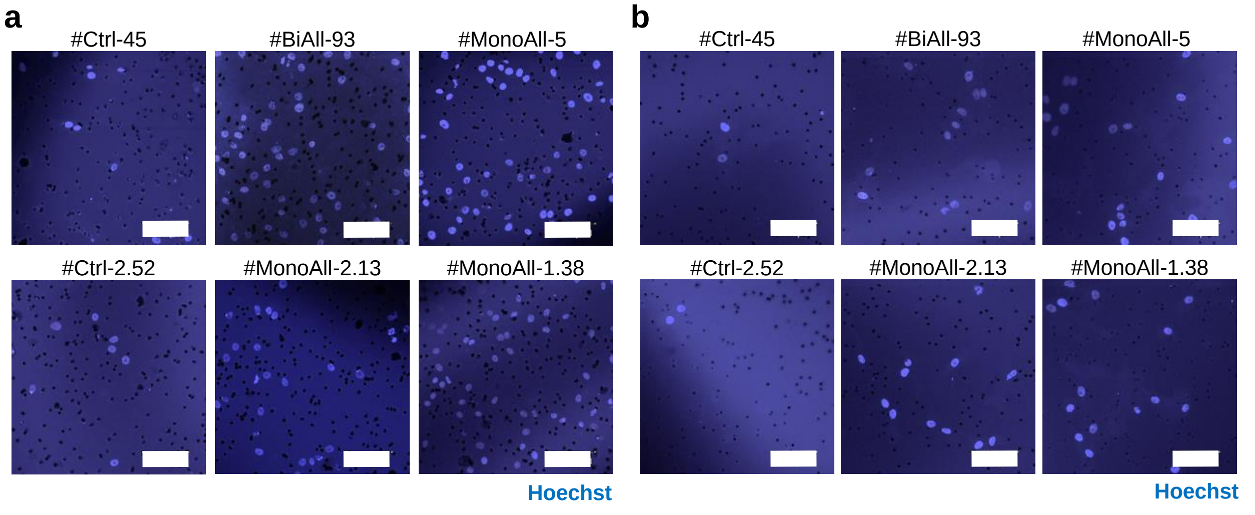

Supplementary Fig. 8 Migration and invasion in control and *PALB2*-mutated cells.
a and **b** Representative images of Transwell® migration (**a**) or Matrigel® -coated invasion (**b**) membranes after removal of non-migrated or -invaded cells and fixation of migrated or invaded cells. Migration and invasion assays were performed three times and ten images of different parts of each membrane were randomly collected. Hoechst-stained nuclei of migrated and invaded cells are seen in light blue. Brightness of the images has been increased and contrast decreased by 20–50% to improve nuclei visibility. Scale bars, 100 µm

Supplementary Fig. 9
IPA Causal Network Analysis of transcriptome data.

a

#BiAll-93

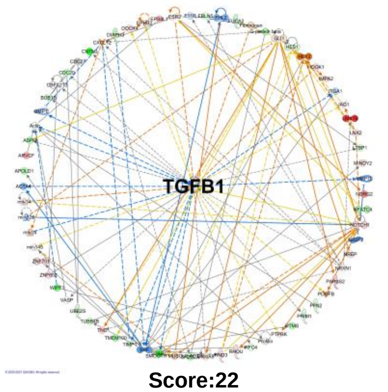

Score:22
Cellular Movement
Cancer
Organismal Injury and Abnormalities

#MonoAll-2.13

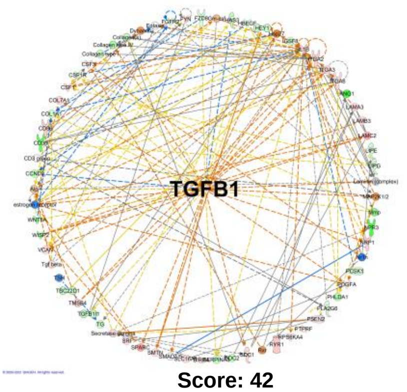

Score: 42
Cellular Movement
Cell-To-Cell Signaling and Interaction
Connective Tissue Disorders

b

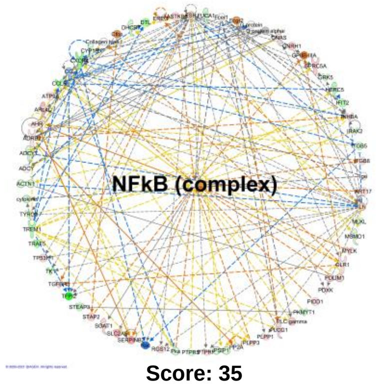

Score: 35
Cell Death and Survival
Cellular Movement
Post-Translational Modification

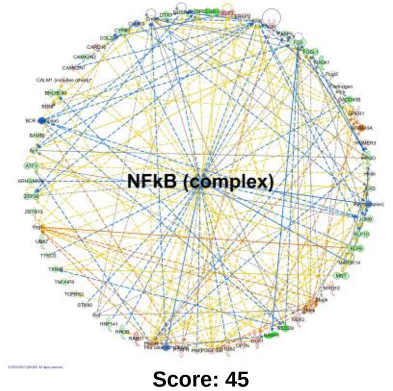

Score: 45
Cell Death and Survival
Neurological Disease
Organismal Injury and Abnormalities

c

Score: 29
Cellular Movement
Immune Cell Trafficking
Organismal Injury and Abnormalities

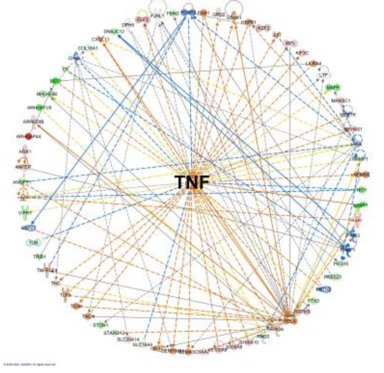

Supplementary Fig. 9 IPA Causal Network Analysis of filtered differentially expressed genes in *PALB2*-mutated cell lines #BiAll-93 and #MonoAll-2.13 presented as radial plots. Causal Network Analysis is an expanded upstream analysis tool in IPA that enables the discovery of novel regulatory mechanisms by utilizing paths that involve more than one link, *i.e.* including intermediate regulators that are not directly connected to dataset targets, to find mechanistic hypotheses to explain the observed expression changes in datasets (Krämer *et al.* 2014). **a–c** Radial plots of causal networks identifying TGFB1 (**a**) or NFkB (**b**) among the most connected regulatory nodes in #BiAll-93 and #MonoAll-2.13, and a network identifying TNF regulatory node (**c**) in #MonoAll-2.13. Causal score (negative exponent of the right-tailed Fisher's exact test result) and the diseases and functions associated with each network are shown. Orange and blue symbols depict activation and inactivation, respectively, green symbols decreased transcription, orange and blue lines signaling leading to activation and inhibition, respectively, and yellow and grey lines indicate that the findings are inconsistent with state of downstream molecule or that the effect is not predicted.

Krämer A, Green J, Pollard J, Tugendreich S. Causal analysis approaches in Ingenuity Pathway Analysis. Bioinformatics (Oxford, England) [Internet]. Bioinformatics; 2014 [cited 2021 Dec 15];30:523–30. Available from: https://pubmed.ncbi.nlm.nih.gov/24336805/

Supplementary Fig. 10

Inhibition of TGFβ signaling partially restores the phenotypes of PALB2-compromised cells.

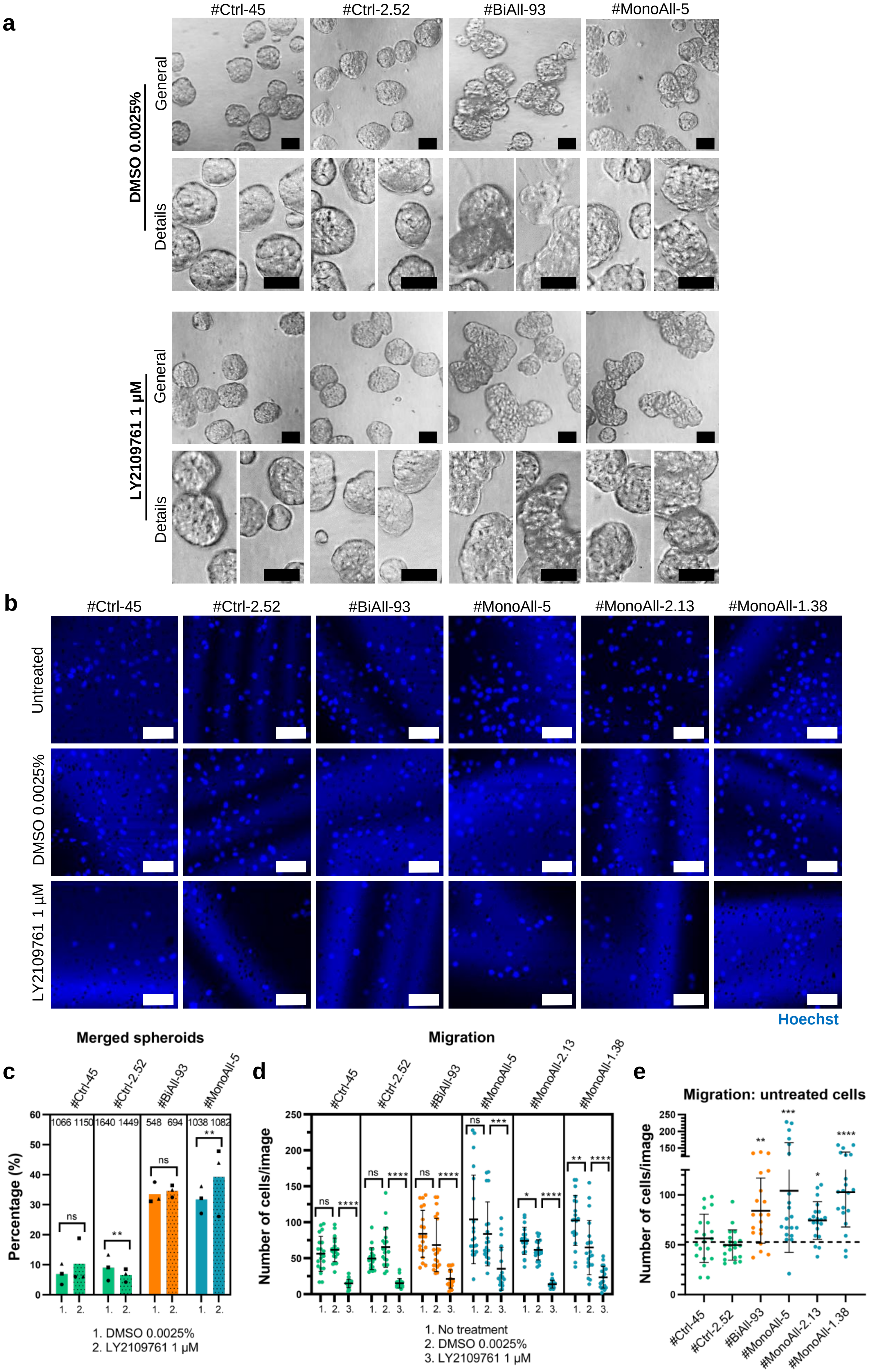

Supplementary Fig. 10 Inhibition of TGFβ signaling partially restores the phenotypes of *PALB2*-mutated cells. **a** Representative low-magnification (general) and high-magnification (details) brightfield images of spheroids treated with 0.0025% DMSO vehicle or 1 µM TGFβ receptor I/II inhibitor LY2109761. Scale bars, 50 µm. **b** Representative images of untreated, 0.0025% DMSO-treated and 1 µM LY2109761-treated Transwell® migration membranes after removal of non-migrated and fixation of migrated cells. Hoechst-stained nuclei of migrated cells are seen in light blue. Brightness of the images has been increased by 20–60%. Scale bars, 100 µm. **c** Proportion (%) of merged spheroids in 3D-cultured cells treated with 0.0025% DMSO or 1 µM LY2109761. Spheroids were grown as triplicates and ten images from different parts of each plate were randomly captured. Each bar represents the mean of the three plates and the total number of analyzed spheroids is given on top of the bar. Statistical significance was determined by pairwise, two-tailed Fisher's exact tests. Circle, plate 1; square, plate 2; triangle, plate 3. **d** Migration of cells without any treatment, and after treatment with 0.0025% DMSO vehicle or 1µM LY2109761 in vehicle. All the assays have been carried out simultaneously. Each dot in the scatter dot plot represents the number of migrated cells per image (area = 0.18 mm^2) in untreated or 0.0025% DMSO-treated cells. Ten images of two replicates (n = 10 x 2) per cell line and treatment were randomly captured. Horizontal lines designate the mean values (± SD). Statistical significance was determined by pairwise, two-tailed Mann-Whitney U tests. **e** Reproduction of the data of the untreated cells in **d** to simplify its comparison to that in Fig. 6a. Dashed line shows the mean of the two control cell lines. **c-e** ns, not significant; **d** SD, standard deviation; *p < 0.05, **p < 0.01, *** p < 0.001, ****p < 0.0001

Supplementary Fig. 11

Effect of excessive TGFβ on DNA aberrations in control and *PALB2*-compromised cells.

a

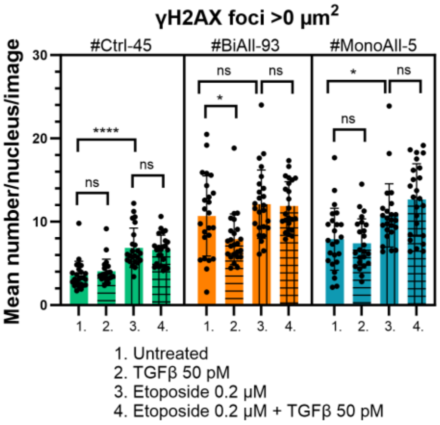

c

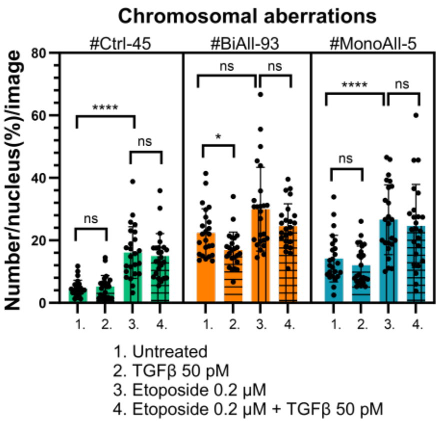

b

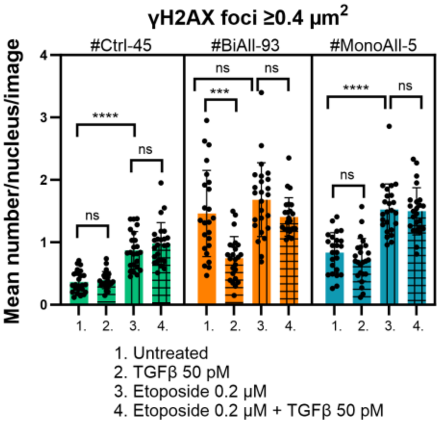

Supplementary Fig. 11 TGFβ does not modify the effects of etoposide but can alleviate basal DNA-damage and cell division errors in biallellically *PALB2*-mutated #BiAll-93 cells. **a-c** Number of all (**a**) and ≥0.4 µm^2 *γ*H2AX foci (**b**) and chromosomal aberrations including micronuclei, lagging chromosomes and chromosome bridges (**c**) in #Ctrl-45, BiAll-93 and #MonoAll-5 cell lines after treatment with 50 pM TGFβ (bars with horisontal lines), 0.2 µM etoposide (bars with vertical lines), or their combination (bars with combined lines) for 48 hours. Six images from four replicates (n = 6 x 4) per cell line and treatment were randomly captured and each dot represents an image. Statistical details from Kruskal-Wallis test with Dunn's multiple comparison post-test are given in Supplementary Table 2f. ns, not significant, *$p < 0.05$, *** $p < 0.001$, ****$p < 0.0001$

Supplementary Fig. 12

KRT14 expression in control and *PALB2*-compromised cells.

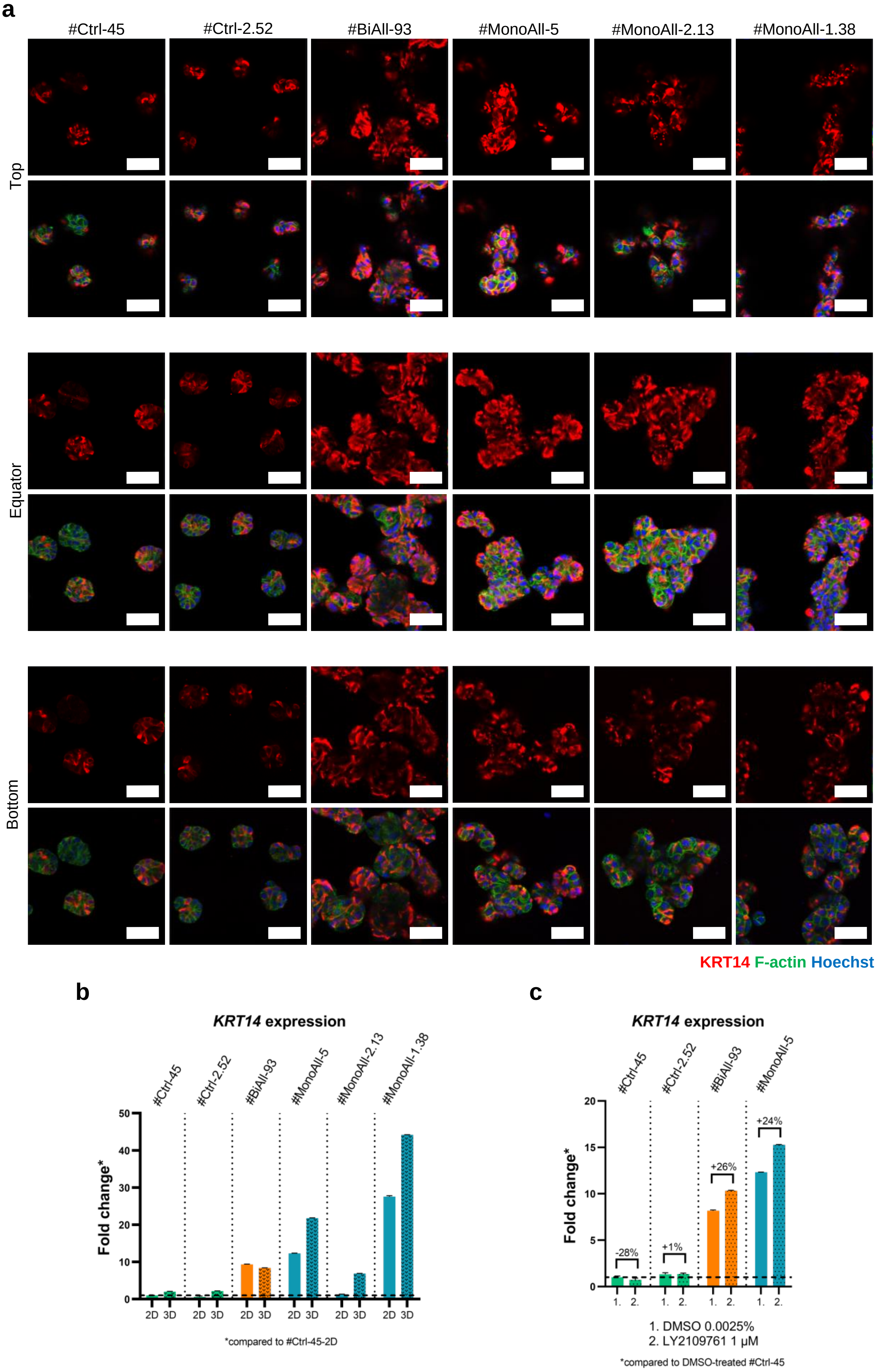

d

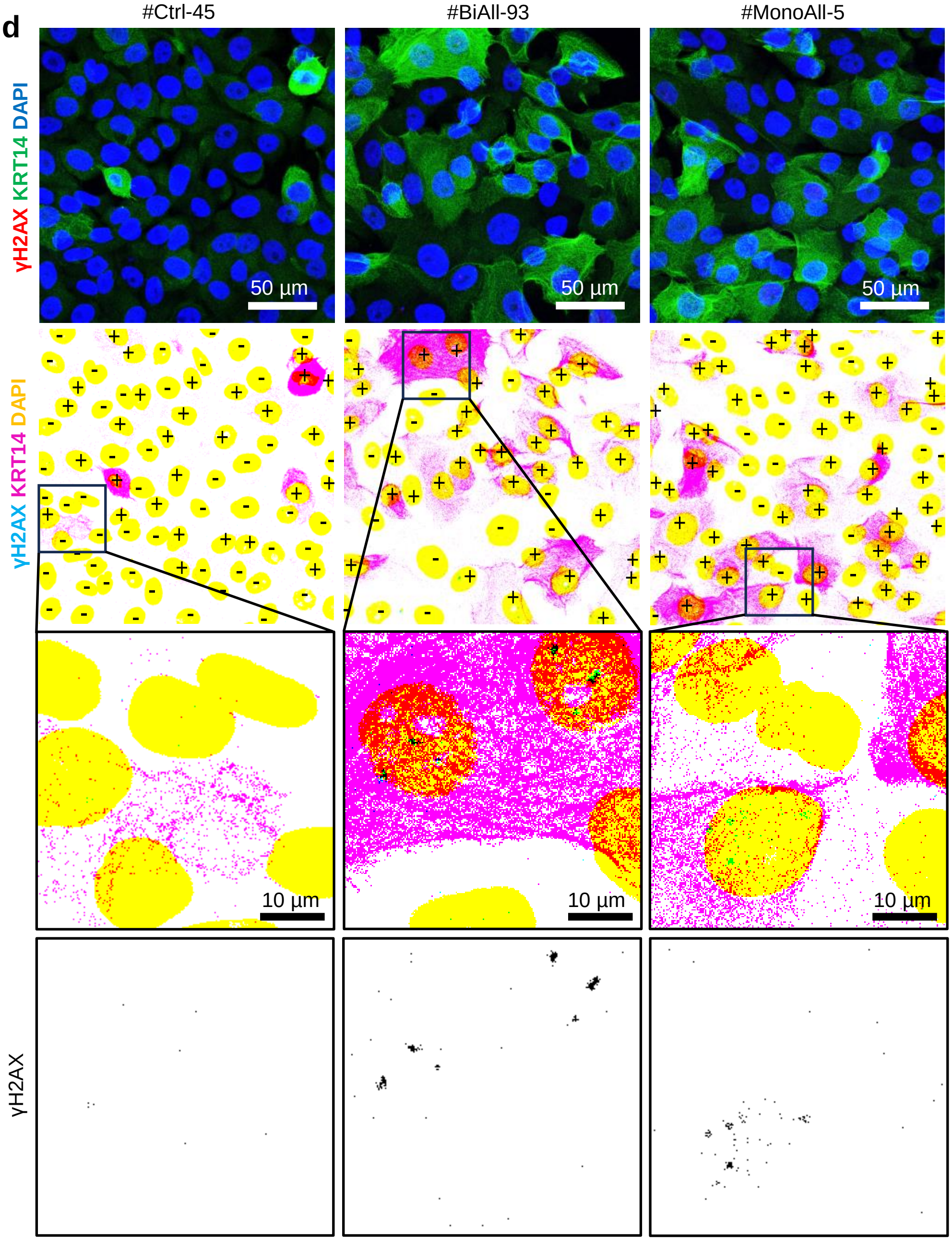
#Ctrl-45
#BiAll-93
#MonoAll-5
γH2AX KRT14 DAPI
50 µm
γH2AX KRT14 DAPI
10 µm
γH2AX

e

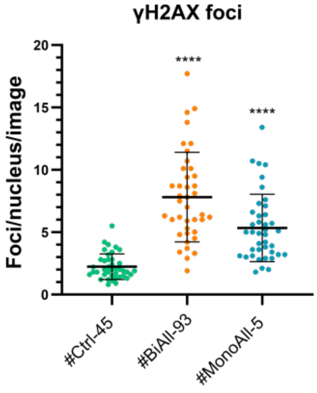
γH2AX foci
Foci/nucleus/image
20
15
10
5
0
****
****
#Ctrl-45
#BiAll-93
#MonoAll-5

f

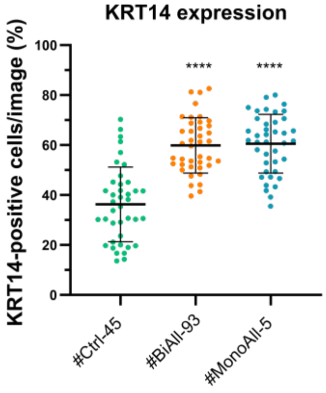
KRT14 expression
KRT14-positive cells/image (%)
100
80
60
40
20
0
****
****
#Ctrl-45
#BiAll-93
#MonoAll-5

Supplementary Fig. 12 *KRT14* expression in control and *PALB2*-mutated cells and spheroids. **a** Top, equatorial and bottom cross section images of spheroids showing DAPI (nuclei, blue), phalloidin (F-actin, green) and KRT14 (red) staining, corresponding to the maximum intensity projection images in Fig. 7c. Brightness of the images has been increased by 50%. Scale bars, 50 µm. **b** *KRT14* expression measured by qRT-PCR in 2D- (plain bars) and 3D-cultured (patterned bars) cells. **c** *KRT14* expression measured by qRT-PCR in 0.0025% DMSO-treated (plain bars) and 1 µm LY2109761-treated (dotted bars) spheroids. *KRT14* expression change (%) in LY2109761-treated spheroids compared to DMSO-treated spheroids within each cell line is shown. **b** and **c** PCR reactions were performed as triplicates and 2^(-delta CT) values were calculated using *GAPDH* and *B2M* as reference genes. Each bar represents the expression level (mean of the triplicates) as fold change compared to 2D-cultured or DMSO-treated #Ctrl-45 (= 1, horizontal dashed line), respectively. Error bars show the SD of delta CT from the triplicates. **d** Representative immunocytochemistry images (top row), corresponding to cropped images in Fig. 7e, showing DAPI (nuclei, blue), γH2AX (red) and KRT14 (green) staining, as well as combined binary images (second row) showing RGB channels in yellow (nuclei), light blue (γH2AX) and magenta (KRT14). KRT14-positive (+) and –negative (-) cells have been defined as shown. Magnifications of selected areas (third row) and corresponding binary images (bottom row) showing only the red channel (γH2AX) are also shown. Brightness and contrast of the images in the top row have been increased by 60% and 20%, respectively. **e** Mean number of γH2AX foci per nucleus per image in control and PALB2-compromised cells in this experiment. Statistical significance was determined by Kruskal-Wallis test with Dunn's multiple comparison post-test. **f** Proportion (%) of cells with *KRT14* expression per image. Statistical significance was determined by Brown-Forsythe and Welch ANOVA with Dunnett's T3 multiple comparison post-test. **e, f** Ten images, each including four z-stack slices, were randomly captured of four separate wells per cell line (n = 10 x 4), and the number of γH2AX foci and KRT14-positive and -negative cells were calculated using imageJ. Each dot in the scatter dot plots represents one image, and the lines designate mean values (±SD, standard deviation). Statistical details are given in Supplementary Table 2g. ****p < 0.0001

Supplementary Fig. 13
Knockdown of *KRT14* partially restores the phenotypes of *PALB2*-compromised cells.

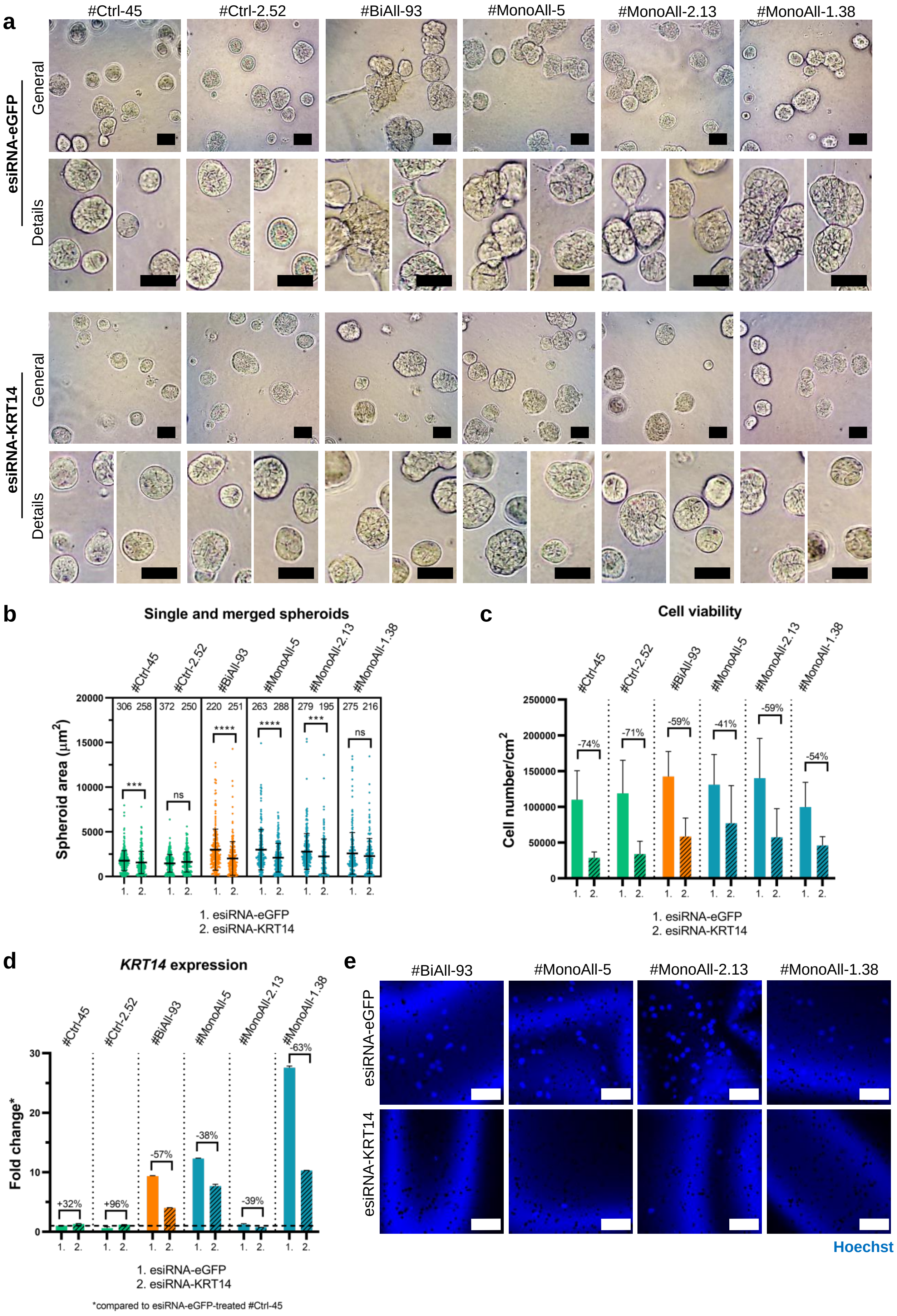

Supplementary Fig. 13 Knockdown of *KRT14* partially restores the phenotypes of *PALB2*-mutated cells. **a** Representative low-magnification (general) and high-magnification (details) brightfield images of control and PALB2-compromised spheroids treated with control esiRNA-eGFP or esiRNA-KRT14. Scale bars, 50 µm. **b** Cross-sectional areas (μm^2) of single and merged spheroids in control and PALB2-compromised cell lines. Horizontal lines designate mean values (±SD). Statistical significance was determined by two-tailed, pairwise Mann-Whitney U tests. ns, not significant; ***$p < 0.001$, and ****$p < 0.0001$. **c** Cell viability measured as number of living cells/cm^2 48 hours after transfection with 10 nM control eGFP-esiRNA (plain bars) or KRT14-esiRNA (striped bars) in control and PALB2-compromised cell lines. The reduction in cell viability (-%) due to KRT14-esiRNA treatment in each cell line is shown. The cells were counted using Countess II automated cell counter (Thermo Fisher). Bars represent the mean of four plates (±SD). The initial seeding density 24 hours before transfection was 16 000 cells/cm^2, except for cell line #BiAll-93, in case of which 21 000 cells/cm^2 were seeded, due to lower proliferation rate compared to the other cell lines. **d** esiRNA-KRT14 knockdown efficiency presented as *KRT14* expression measured by qRT-PCR in esiRNA-eGFP-treated (plain bars) and esiRNA-KRT14-treated (striped bars) cells. PCR reactions were performed as duplicates and 2^(-delta CT) values were calculated using *GAPDH* and *B2M* as reference genes. Each bar represents the expression level (mean of the duplicates) as fold change compared to esiRNA-eGFP-treated #Ctrl-45 (=1, horizontal dashed line). Error bars show the SD of delta CT from the duplicates. *KRT14* expression change (%) in esiRNA-KRT14-treated cells compared to esiRNA-eGFP-treated cells within each cell line is also shown. **b** and **c** SD, standard deviation. **e** Representative images of esiRNA-eGFP- and esiRNA-KRT14-treated Transwell® migration membranes after removal of non-migrated and fixation of migrated cells. Hoechst-stained nuclei of migrated and invaded cells are seen in light blue. Brightness of the images has been increased by 20–60%. Scale bars, 100 µm

Supplementary Table 3 Expression alterations of selected genes in *PALB2*-compromised cell lines assayed by RT2 qPCR and their comparison to transcriptome sequencing results analysed by Chipster.

	**Fold changes in RT-qPCR:**<br>- **upper number = *PALB2*-compromised cell line vs. combined #Ctrl-45 & #Ctrl-2.52**<br>- **lower number = #BiAll-93 and #MonoAll-5 vs #Ctrl-45 and other monoallelic cell lines vs. #Ctrl-2.52**				**Fold changes in RNAseq***	
**Gene**	**#BiAll-93**	**#MonoAll-5**	**#MonoAll-2.13**	**#MonoAll-1.38**	**#BiAll-93 vs #Ctrl-45**	**#MonoAll-2.13 vs #Ctrl-2.52**
BMP2	4.5<br>4.9	2.0<br>2.1	NS**<br>-1.3	1.4<br>1.3	5.6***	NS
BST2	-331.8<br>-369.3	-98.8<br>-109.9	-86.2<br>-77.5	-80.0<br>-71.9	-93.7	-32.7
CCNB2	-11.1<br>-13.8	NS<br>-1.7	NS<br>-1.2	NS<br>1.4	-2.5	NS
CDK1	-12.7<br>-19.8	NS<br>-2.0	NS<br>-1.1	NS<br>2.0	-2.5	NS
E2F1	-1.8<br>-2.2	NS<br>NS	-2.8<br>-2.2	1.9<br>2.4	-2.0	NS
ELF3	4.1<br>5.0	1.8<br>2.1	6.1<br>5.0	-2.6<br>-3.1	2.9	2.6
ESRP1	-29.9<br>-26.7	-10.2<br>-9.1	NS<br>1.0	-9.1<br>-10.1	-4.3	NS
EYA2	7.8<br>6.2	3.5<br>NS	4.6<br>5.9	6.7<br>8.5	5.2	4.8
FGFR3	2.0<br>3.1	NS<br>2.7	NS<br>-1.3	NS<br>-1.0	2.8	1.5
GDA	79.9<br>931.2	NS<br>3.3	55.3<br>4.8	NS<br>-6.7	141.0	4.3
HIST1H3F	-24.0<br>-27.0	-1.8<br>-2.0	-3.5<br>-3.1	NS<br>NS	-4.5	-1.7
HIST1H4C	-2.0<br>-2.8	NS<br>-1.8	NS<br>NS	NS<br>NS	-1.9	NS
JAK2	NS<br>1.1	NS<br>1.1	1.8<br>1.5	-1.5<br>-1.8	2.1	NS
JAM3	86.9<br>74.2	51.2<br>43.7	73.7<br>86.3	40.2<br>47.0	37.0	69.1
KRT14	4.0<br>4.1	10.4<br>10.8	3.3<br>3.2	21.1<br>20.4	5.4	4.3
PARVB	41.7<br>17.4	21.3<br>8.9	32.4<br>77.6	22.6<br>54.1	7.9	29.0
PLAC8	6.3<br>4.3	5.6<br>3.9	1.9<br>2.7	2.9<br>4.2	3.5	NS
PLK1	-12.6<br>-14.0	-1.3<br>-1.5	-1.5<br>-1.4	1.6<br>-1.5	-2.5	NS
PPP2R2C	6.0<br>4.5	1.7<br>1.3	NS<br>NS	1.6<br>2.2	1.7	1.9
SPRR1B	7.6<br>11.9	3.0<br>4.7	NS<br>-1.5	5.5<br>3.5	7.3	NS
UCHL1	-5.0<br>-6.9	-10.3<br>-14.0	-12.9<br>-9.5	-15.1<br>-11.1	-3.5	-2.0

*RNAseq log2-fold change results turned into fold regulation.

**NS, not significant – *p*- and *q*-value is >0.05 for the fold change in RT-qPCR and RNAseq, respectively.

***Numerical values for fold changes are not directly comparable between the methods since their sensitivity differs.
